# MicroRNA-mediated responses to long-term magnesium-deficiency in *Citrus sinensis* roots revealed by Illumina sequencing

**DOI:** 10.1186/s12864-017-3999-5

**Published:** 2017-08-24

**Authors:** Wei-Wei Liang, Jing-Hao Huang, Chun-Ping Li, Lin-Tong Yang, Xin Ye, Dan Lin, Li-Song Chen

**Affiliations:** 1 Institute of Plant Nutritional Physiology and Molecular Biology, College of Resources and Environment, Fujian Agriculture and Forestry University, Fuzhou, 350002 China; 2 Pomological Institute, Fujian Academy of Agricultural Sciences, Fuzhou, 350013 China; 3 Fujian Provincial Key Laboratory of Soil Environmental Health and Regulation, College of Resources and Environment, Fujian Agriculture and Forestry University, Fuzhou, 350002 China; 4 The Higher Educational Key Laboratory of Fujian Province for Soil Ecosystem Health and Regulation, Fujian Agriculture and Forestry University, Fuzhou, 350002 China

**Keywords:** *Citrus sinensis*, Illumina sequencing, Magnesium-deficiency, miRNA, Root

## Abstract

**Background:**

Magnesium (Mg)-deficiency occurs most frequently in strongly acidic, sandy soils. *Citrus* are grown mainly on acidic and strong acidic soils. Mg-deficiency causes poor fruit quality and low fruit yield in some *Citrus* orchards. For the first time, we investigated Mg-deficiency-responsive miRNAs in ‘Xuegan’ (*Citrus sinensis*) roots using Illumina sequencing in order to obtain some miRNAs presumably responsible for *Citrus* Mg-deficiency tolerance.

**Results:**

We obtained 101 (69) miRNAs with increased (decreased) expression from Mg-starved roots. Our results suggested that the adaptation of *Citrus* roots to Mg-deficiency was related to the several aspects: (*a*) inhibiting root respiration and related gene expression via inducing *miR158* and *miR2919*; (*b*) enhancing antioxidant system by down-regulating related miRNAs (*miR780*, *miR6190*, *miR1044*, *miR5261* and *miR1151*) and the adaptation to low-phosphorus (*miR6190*); (*c*) activating transport-related genes by altering the expression of *miR6190*, *miR6485*, *miR1044*, *miR5029* and *miR3437*; (*d*) elevating protein ubiquitination due to decreased expression levels of *miR1044*, *miR5261*, *miR1151* and *miR5029*; (*e*) maintaining root growth by regulating *miR5261*, *miR6485* and *miR158* expression; and (*f*) triggering DNA repair (transcription regulation) by regulating *miR5176* and *miR6485* (*miR6028*, *miR6190*, *miR6485*, *miR5621, miR160* and *miR7708*) expression. Mg-deficiency-responsive miRNAs involved in root signal transduction also had functions in *Citrus* Mg-deficiency tolerance.

**Conclusions:**

We obtained several novel Mg-deficiency-responsive miRNAs (i.e., miR5261, miR158, miR6190, miR6485, miR1151 and miR1044) possibly contributing to Mg-deficiency tolerance. These results revealed some novel clues on the miRNA-mediated adaptation to nutrient deficiencies in higher plants.

**Electronic supplementary material:**

The online version of this article (doi:10.1186/s12864-017-3999-5) contains supplementary material, which is available to authorized users.

## Background

Magnesium (Mg)-deficiency, a common problem in many agricultural crops, occurs most frequently in strongly acidic, sandy soils, where Mg is very prone to leaching [1]. *Citrus* are grown mainly on acidic and strong acidic soils and Mg-deficiency is responsible for the poor fruit quality and the reduction in fruit yield in some *Citrus* orchards [2]. According to our investigation in 2011, over 90% and 77% of *Citrus grandis* orchard soils from Pinghe county, Fujian province had a pH less than 5.0 and a soil exchange Mg content less than the optimum range, respectively [3]. What’s worse, crop Mg-deficiency, which is becoming more and more popular due to soil acidification and improper farmer practices such as intensive crop production systems and highly fortified rotation, has been considered to be an urgent agricultural problem [3, 4]. Although Mg is one of the most important nutrients in higher plants and plays essential roles in numerous cellular processes such as chlorophyll biosynthesis, gas exchanges [2, 5–7], conformational stabilization of proteins, nucleic acids, cell walls and membranes [8], partitioning and utilization of photoassimilates [7, 9], activation of enzymes [9, 10] and reactive oxygen species (ROS) generation [9]. Despite the important roles of Mg in higher plants, Mg has been less paid attention by agronomists and botanists relative to the other nutrients and is considered to be “the forgotten element” [4, 11]. Therefore, it is very important to elucidate the molecular mechanisms on Mg-deficiency impairments and tolerance in higher plants. To our knowledge, such data are rare [10, 12, 13].

Evidence demonstrates that microRNA (miRNA)-mediated posttranscriptional regulation of gene expression plays a role in plant adaptive responses to deficiencies of phosphorus (P), potassium (K), nitrogen (N), sulfur (S), manganese (Mn), boron (B), zinc (Zn) and iron (Fe) [14–20]. Numerous differentially expressed miRNAs have been isolated from P-starved *Arabidopsis*, white lupin, *Medicago truncatula*, common bean, rice, barley, tomato and soybean [21–25]. The roles of P-deficiency-induced up-regulation of plant *miR399* and *miR827* in the maintenance of P homeostasis via inhibiting their targets *ubiquitin-conjugating enzyme E2 24* (*UBC24*) and *N limitation adaptation* (*NLA*), respectively have been well characterized [14, 23, 26, 27].

Nitrogen-deficiency-induced alterations of miRNA profiles have been reported on several higher plants including maize, *Arabidopsis*, soybean, common bean [20, 24, 25, 28, 29]. Several N-deficiency-responsive miRNAs have been characterized in some details. For example, root modulation under N-deficiency was coordinated by miR160, miR167 and miR171 and root growth was promoted by down-regulating *miR167* expression and up-regulating *miR160* and *miR171* expression [24, 28]. N-deficiency-induced down-regulation of *miR169* has been demonstrated to be an adaptive strategy of plants to N-starvation via N-uptake and remobilization [24, 30].

Despite the vital roles of K in higher plants, little is known about K-deficiency-responsive miRNAs. In a study, Yan et al. [31] examined K-deficiency-induced alterations in expression of *miR444a* and its targets (i.e., *MADS-57*, *MADS-27b*, *MADS-27a* and *MADS-23*) in rice roots, and found that *miR444a* was slightly down-regulated and *MADS-23* was greatly up-regulated.

In addition, many differentially expressed miRNAs have been identified in B-deprived *C. sinensis* roots and leaves [17, 18], Cu-starved [32] and Fe-deficient [33] *Arabidopsis*, S-deprived *Brassica rapus* [34], Mn-limited *Phaseolus vulgaris* [25] and Zn-deficient *Sorghum bicolor* [35].

Although the effects of nutrient deficiencies on miRNA expression in higher plants have been explored by some workers, most of these studies have been paid to herbaceous plants. Little is known about Mg-deficiency-induced alterations of miRNA expression in woody plants. Previously, we examined Mg-deficiency-responsive miRNAs in *C. sinensis* leaves revealed by Illumina sequencing and identified 71 down- and 75 up-regulated miRNAs, implying the potential roles of miRNAs in *Citrus* Mg-deficiency tolerance [36]. On this basis, we used Illumina sequencing to sequence two small RNA libraries from Mg-sufficient (control) and -deficient *C. sinensis* roots in order to distinguish the differences in Mg-deficiency-induced alterations of miRNA profiles between *C. sinensis* roots and leaves and to obtain some miRNAs presumably responsible for *Citrus* Mg-deficiency tolerance.

## Results

### Root dry weight (DW) and root and leaf Mg

**Fig. 1:**
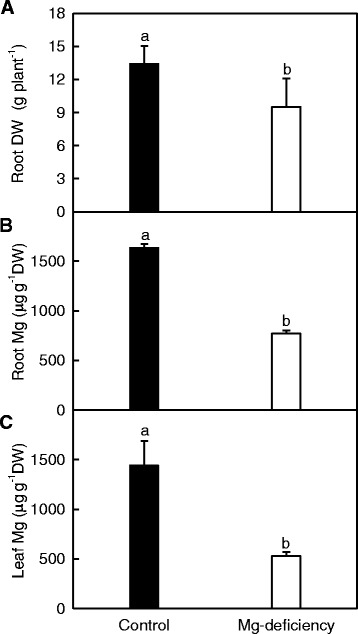
Root DW (**a**), root (**b**) and leaf (**c**) Mg concentrations in response to Mg-deficiency. Bars represent mean ± SD (*n* = 5 for root and leaf Mg and 9 for root DW). Different letters above the *bars* indicate a significant difference at *P* < 0.05

Root DW and root and leaf Mg levels were lower in 0 mM Mg-treated seedlings than in 1 mM Mg-treated ones, and Mg level in leaves from 0 mM Mg-treated seedlings was much less than the sufficient range (Fig. 1) [37]. Based on these data and our previous reports [6, 12], these seedlings submitted to 0 and 1 mM Mg were regarded as Mg-deficient and -sufficient (control), respectively.

### Illumina sequencing and miRNA annotation

**Fig. 2:**
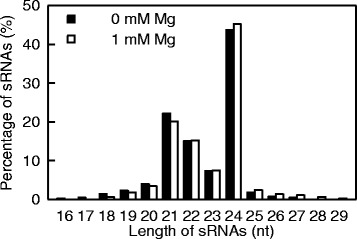
Small RNA length distribution from Mg-deficient and -sufficient *C. sinensis* roots

Using high-throughput sequencing, we got 20,726,716 (22,139,574) raw reads from sRNA library constructed from control (Mg-deficient) roots. After the adaptors, low quality tags and contaminants being removed, the control and Mg-deficient root sRNA libraries generated 20,325,777 (5,561,214) and 21,783,568 (6,124,980) clear reads (unique reads), respectively (Table 1). As shown in Fig. 2, the majority of the clear reads fell within the range of 18–25 nt. The most abundant clear reads were 24 nt length, followed by 21, 22, 23 and 20 nt length. This agrees with the previous data obtained on leaves, roots [17, 18] and fruits [38] of *C. sinensis*, and fruits and flowers of *Citrus trifoliata* [39]. Therefore, these data obtained via high-throughput sequencing of sRNA libraries are reliable. Mg-deficiency increased and decreased the abundances of 24 and 21 nt reads, respectively.

**Table 1:** Summary of sRNA sequencing data from Mg-sufficient and -deficient *Citrus sinensis* roots

	Mg-sufficiency		Mg-deficiency	
	Unique sRNAs	Total sRNAs	Unique sRNAs	Total sRNAs
Raw reads		20,726,716		22,139,574
Clear reads	5,561,214(100%)	20,325,777(100%)	6,124,980(100%)	21,783,568(100%)
Mapped to genomic	3,077,845(55.34%)	13,624,836(67.03%)	3,378,231(55.15%)	14,510,776(66.61%)
ᅟExon antisense	47,462(0.85%)	176,617(0.87%)	50,014(0.82%)	177,516(0.81%)
ᅟExon sense	105,888(1.90%)	358,233(1.76%)	117,753(1.92%)	372,942(1.71%)
ᅟIntron antisense	65,331(1.17%)	281,377(1.38%)	71,109(1.16%)	294,537(1.35%)
ᅟIntron sense	88,455(1.59%)	520,695(2.56%)	94,926(1.55%)	557,578(2.55%)
ᅟmiRNA	54,043(0.97%)	3,125,403(15.37%)	53,522(0.87%)	3,364,650(15.45%)
ᅟrRNA	125,351(2.25%)	2,205,674(10.85%)	157,937(2.58%)	2,558,877(11.74%)
ᅟrepeat	1384(0.02%)	3652(0.02%)	1587(0.03%)	3946(0.02%)
ᅟsnRNA	2722(0.05%)	9423(0.05%)	3188(0.05%)	10,142(0.05%)
ᅟsnoRNA	1667(0.03%)	6123(0.03%)	1833(0.03%)	6354(0.03%)
ᅟtRNA	18,177(0.33%)	767,772(3.78%)	22,291(0.36%)	660,793(3.03%)
ᅟUnannotated sRNAs	5,050,734(90.82%)	12,870,808(63.32%)	5,550,820(90.63%)	13,776,233(63.24%)

Here, 13,624,836 clean reads (3,077,845 unique reads) from Mg-sufficient roots and 14,510,776 clear reads (3,378,231 unique reads) from Mg-deficient roots were mapped to *C. sinensis* genome (JGIversion1.1, http://phytozome.jgi.doe.gov/pz/portal.html#!info?alias=Org_Csinensis) using SOAP [40]. Thereafter, we used the unannotated 5,050,734 and 5,550,820 unique reads from Mg-sufficient and -deficient roots, respectively to predict novel miRNAs (Table 1).

### Identification and prediction of root miRNAs

As shown in Additional file 1, we identified 733 known miRNAs in *C. sinensis* roots. To avert false results due to the use of low abundant miRNAs, these known miRNAs with a transcript per million (TPM) value <10 in both Mg-sufficient and -deficient roots were not utilized for further analysis [17, 41]. The remained 300 miRNAs with a TPM value ≥10 in Mg-sufficient and/or -deficient roots were utilized for Mg-deficiency-responsive miRNA analysis (Additional file 2). As shown in Additional file 3, we obtained 71 up- and 54 down-regulated known miRNAs from Mg-deficient roots.

As shown in Additional files 4, 5, and 6, we identified 181 novel miRNAs in both Mg-sufficient and -deficient roots, and 30 up- and 15 down-regulated novel miRNAs in Mg-deficient roots with a TPM value ≥ ten in Mg-deficient and/or -sufficient roots.

### Validation of sequencing data by stem-loop qRT-PCR

**Fig. 3:**
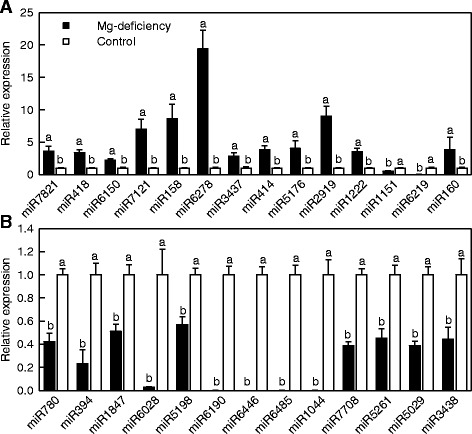
Relative expression levels of selected Mg-deficiency-responsive known miRNAs in Mg-deficient and control roots revealed by qRT-PCR. *Bars* represent mean ± SD (*n* = 3). For the same miRNA, different *letters above the bars* indicate a significant difference at *P* < 0.05. All the values were expressed relative to the control roots

The expression levels of 27 Mg-deficiency-responsive miRNAs were assayed by stem-loop qRT-PCR. Except for *miR1222*, the expression patterns of all miRNAs obtained by stem-loop qRT-PCR and Illumiona sequencing were similar (Fig. 3 and Table 2). Thus, the results produced by Illumiona sequencing were reliable.

**Table 2:** qRT-PCR analysis of predicted target genes for selected Mg-deficiency-responsive known miRNAs in *C. sinensis* roots

miRNA	Fold change of miRNA	Accession	Homology	Target genes	Potential roles	Relative change of target genes
miR158	9.23711361**	**orange1.1g022993m**	**AT5G62740.1**	**SPFH/Band 7/PHB domain-containing membrane-associated protein family**	**Stress response**	0.73**
miR1222	−13.23073355**	orange1.1g037429m	AT4G27220.1	NB-ARC domain-containing disease resistance protein	Disease resistance protein	ND
miR2919	6.01101607**	**orange1.1g002089m**	**AT3G14940.1**	**Phosphoenolpyruvate carboxylase 3**	**Carbohydrate metabolism**	0.53**
miR3437	3.97352136**	**orange1.1g040557m**	**AT1G56140.1**	**Leucine-rich repeat transmembrane protein kinase**	**Transmembrane signal transduction**	0.65**
miR7821	4.13197145**	orange1.1g010745m	AT1G29760.1	Putative adipose-regulatory protein (Seipin)	Triacylglycerol accumulation and LD proliferation	0.95
miR394	−5.44692358**	orange1.1g000114m	AT1G20960.1	U5 small nuclear ribonucleoprotein helicase, putative	mRNA processing	1.48
miR414	3.01957377**	orange1.1g004767m	AT1G17980.1	Poly(A) polymerase 1	mRNA processing	0.76
		**orange1.1g006232m**	**AT1G17980.1**	**Poly(A) polymerase 1**	**mRNA processing**	0.72**
miR418	2.16768709**	orange1.1g003146m	AT1G20780.1	Senescence-associated E3 ubiquitin ligase 1	Ubl conjugation pathway	0.78
miR6150	8.95879131**	orange1.1g009434m	AT5G62810.1	Peroxin 14	Protein import into peroxisome matrix, docking	3.91**
		orange1.1g009573m	AT5G62810.1	Peroxin 14	Protein import into peroxisome matrix, docking	2.36*
		orange1.1g018459m	AT3G28715.1	ATPase, V0/A0 complex, subunit C/D	ATP hydrolysis coupled proton transport	0.82
miR6278	8.85634619**	orange1.1g005896m	AT3G14470.1	NB-ARC domain-containing disease resistance protein	Disease resistance protein	1.63**
		orange1.1g030696m	AT5G17840.1	DnaJ/Hsp40 cysteine-rich domain superfamily protein	Stress response	1.56**
miR1847	−2.21361107**	**orange1.1g026316m**	**AT5G35530.1**	**Ribosomal protein S3 family protein**	**Translation**	2.76**
		**orange1.1g026835m**	**AT5G35530.1**	**Ribosomal protein S3 family protein**	**Translation**	1.84*
		orange1.1g029201m	AT2G31610.1	Ribosomal protein S3 family protein	Translation	1.91
miR6028	−2.29912898**	orange1.1g005923m	AT2G33580.1	LysM-containing receptor-like kinase 5	Transmembrane signal transduction	0.67**
		orange1.1g034040m	AT5G42990.1	Ubiquitin-conjugating enzyme 18	Protein ubiquitination	ND
		orange1.1g021729m	AT4G29100.1	Basic helix-loop-helix (bHLH) DNA-binding superfamily protein	Transcription factor	ND
		orange1.1g026539m	AT1G79020.1	Enhancer of polycomb-like transcription factor protein	Transcription regulation	ND
		orange1.1g045123m	AT4G35800.1	RNA polymerase II large subunit	mRNA synthesis	ND
		orange1.1g003175m	AT4G14700.1	Origin recognition complex 1	DNA synthesis and replication	0.57**
		**orange1.1g006076m**	**AT3G46790.1**	**Tetratricopeptide repeat (TPR)-like superfamily protein**		6.01**
		**orange1.1g029970m**	**AT3G49940.1**	**LOB domain-containing protein 38**		6.05**
		**orange1.1g028357m**	**AT2G45850.2**	**AT-hook motif nuclear-localized protein 9 (AHL9)**	**Transcription factor**	6.12**
miR5176	4.58746604**	orange1.1g005789m	AT4G09140.1	MUTL-homologue 1	DNA mismatch repair	3.21**
		orange1.1g008397m	AT4G09140.1	MUTL-homologue 1	DNA mismatch repair	2.87**
		orange1.1g010846m	AT4G09140.1	MUTL-homologue 1	DNA mismatch repair	3.13**
		orange1.1g012406m	AT4G09140.1	MUTL-homologue 1	DNA mismatch repair	5.39*
miR7121	4.74373348**	**orange1.1g005267m**	**AT1G71400.1**	**Receptor like protein 12**	**Hormone-mediated signaling pathway**	0.66**
		**orange1.1g005542m**	**AT1G71400.1**	**Receptor like protein 12**	**Hormone-mediated signaling pathway**	0.77**
		orange1.1g008628m	AT1G71400.1	Receptor like protein 12	Hormone-mediated signaling pathway	1.76**
		orange1.1g002167m	AT5G27060.1	Receptor like protein 53	Hormone-mediated signaling pathway	5.01**
		orange1.1g012980m	AT5G53390.1	O-acyltransferase (WSD1-like) family protein	Lipid and fatty-acid metabolism	0.86
		**orange1.1g013532m**	**AT5G53390.1**	**O-acyltransferase (WSD1-like) family protein**	**Lipid and fatty-acid metabolism**	0.72**
		**orange1.1g027358m**	**AT5G03080.1**	**Phosphatidic acid phosphatase (PAP2) family protein**	**Dephosphorylation**	0.53**
		**orange1.1g027353m**	**AT5G03080.1**	**Phosphatidic acid phosphatase (PAP2) family protein**	**Dephosphorylation**	0.56**
miR6190	−3.61190068**	**orange1.1g029300m**	**AT5G64200.1**	**Ortholog of human splicing factor SC35**	**SR protein**	2.65**
		**orange1.1g017284m**	**AT5G34850.1**	**Purple acid phosphatase 26**	**Phosphate ion homeostasis**	1.82**
		**orange1.1g002842m**	**AT4G01810.1**	**Sec23/Sec24 protein transport family protein**	**Intracellular protein transport**	1.64**
miR6446	−3.77151631**	**orange1.1g016909m**	**AT5G09300.1**	**Thiamin diphosphate-binding fold (THDP-binding) superfamily protein**	**Lipid and fatty-acid metabolism**	3.23**
		**orange1.1g023827m**	**AT5G09300.1**	**Thiamin diphosphate-binding fold (THDP-binding) superfamily protein**	**Lipid and fatty-acid metabolism**	2.56**
		**orange1.1g001557m**	**AT5G20280.1**	**Sucrose phosphate synthase 1F**	**C-compound and carbohydrate metabolism**	3.51**
		**orange1.1g002665m**	**AT5G20280.1**	**Sucrose phosphate synthase 1F**	**C-compound and carbohydrate metabolism**	3.93**
miR6485	−4.69704327**	**orange1.1g001969m**	**AT5G20730.2**	**Transcriptional factor B3 family protein / auxin-responsive factor AUX/IAA-related**	**Transcription factor**	1.41*
		**orange1.1g011274m**	**AT3G22810.1**	**Plant protein of unknown function (DUF828) with plant pleckstrin homology-like region**		1.68**
		orange1.1g031218m	AT1G07400.1	HSP20-like chaperones superfamily protein	Stress response	0.88
		orange1.1g009779m	AT1G08960.1	Cation exchanger 11	Transport	0.57**
		orange1.1g029454m	AT5G51160.1	Ankyrin repeat family protein		0.20**
		**orange1.1g013633m**	**AT1G28560.1**	**SnRNA activating complex family protein**	**Auxin signaling pathway**	2.27**
		**orange1.1g017698m**	**AT1G28560.1**	**SnRNA activating complex family protein**	**Auxin signaling pathway**	1.33**
		orange1.1g042988m	AT5G62850.1	Nodulin MtN3 family protein	Transport	0.59**
		**orange1.1g007868m**	**AT1G72650.2**	**Myb family transcription factor TRFL6**	**Transcription factor**	4.39**
		orange1.1g046667m	AT2G38940.1	Phosphate transporter 1;4	Phosphate transport	0.91
		**orange1.1g001289m**	**AT1G14610.1**	**Valyl-tRNA synthetase / valine-tRNA ligase (VALRS)**	**Protein biosynthesis**	1.75**
		**orange1.1g001303m**	**AT1G14610.1**	**Valyl-tRNA synthetase / valine-tRNA ligase (VALRS)**	**Protein biosynthesis**	1.99**
		orange1.1g001757m	AT1G14610.1	Valyl-tRNA synthetase / valine-tRNA ligase (VALRS)	Protein biosynthesis	1.11
		orange1.1g024117m	AT2G47920.1	Kinase interacting (KIP1-like) family protein		0.21**
		**orange1.1g036588m**	**AT4G20140.1**	**Leucine-rich repeat transmembrane protein kinase**	**Transmembrane signal transduction**	4.54*
		**orange1.1g003591m**	**AT5G05680.1**	**Nuclear pore complex protein NUP88**	**mRNA transport, protein transport**	1.59*
miR1044	−5.19771615**	**orange1.1g001378m**	**AT1G10170.1**	**NF-X-like 1**	**Protein ubiquitination**	2.00**
		**orange1.1g001376m**	**AT1G10170.1**	**NF-X-like 1**	**Protein ubiquitination**	2.61*
		**orange1.1g047796m**	**AT2G38380.1**	**Peroxidase superfamily protein**	**Stress response**	1.97**
		**orange1.1g042193m**	**AT5G03340.1**	**ATPase, AAA-type, CDC48 protein**	**Cell cycle, cell division, protein transport, transport**	4.02*
		orange1.1g019546m	AT2G40340.1	Integrase-type DNA-binding superfamily protein	Abscisic acid signaling pathway	0.60**
miR5198	−5.36879795**	**orange1.1g002063m**	**AT1G72180.1**	**Leucine-rich receptor-like protein kinase family protein**	**Transmembrane signal transduction**	1.84**
miR5029	−5.99278869**	orange1.1g012168m	AT5G53450.1	OBP3-responsive gene 1		0.03**
		**orange1.1g026587m**	**AT4G31300.3**	**Proteasome subunit beta type-6 (PBA1)**	**Protein ubiquitination**	2.26**
		**orange1.1g029964m**	**AT4G31300.3**	**Proteasome subunit beta type-6 (PBA1)**	**Protein ubiquitination**	2.21**
		**orange1.1g030788m**	**AT4G31300.3**	**Proteasome subunit beta type-6 (PBA1)**	**Protein ubiquitination**	2.91**
		**orange1.1g014625m**	**AT3G23510.1**	**Cyclopropane-fatty-acyl-phospholipid synthase**	**Lipid and fatty-acid metabolism**	2.44**
		**orange1.1g018123m**	**AT3G44160.1**	**Outer membrane OMP85 family protein**	**Transmembrane transport**	1.43*
miR5261	−6.08070339**	**orange1.1g018132m**	**AT3G56930.1**	**DHHC-type zinc finger family protein**		1.94**
		**orange1.1g010695m**	**AT3G12640.1**	**RNA binding (RRM/RBD/RNP motifs) family protein**	**mRNA processing**	2.40**
		**orange1.1g011967m**	**AT3G12640.1**	**RNA binding (RRM/RBD/RNP motifs) family protein**	**mRNA processing**	2.80**
		**orange1.1g031636m**	**AT1G67620.1**	**Lojap-related protein**		2.41**
		**orange1.1g033883m**	**AT1G67620.1**	**Lojap-related protein**		2.03**
		**orange1.1g004959m**	**AT5G66850.1**	**Mitogen-activated protein kinase kinase kinase 5**	**Intracellular signalling**	1.97*
		**orange1.1g043928m**	**AT2G36110.1**	**Polynucleotidyl transferase, ribonuclease H-like superfamily protein**	**3′-5′ exonuclease activity**	3.32**
		**orange1.1g037980m**	**AT2G36110.1**	**Polynucleotidyl transferase, ribonuclease H-like superfamily protein**	**3′-5′ exonuclease activity**	2.17**
		**orange1.1g004713m**	**AT5G54260.1**	**DNA repair and meiosis protein (Mre11)**	**DNA damage, DNA repair, meiosis**	4.47**
		**orange1.1g010785m**	**AT3G26020.2**	**Protein phosphatase 2A regulatory B subunit family protein**	**Intracellular signalling**	2.01*
		**orange1.1g000012m**	**AT1G55860.2**	**Ubiquitin-protein ligase 1 (E3 ubiquitin-protein ligase UPL1)**	**Protein ubiquitination**	2.05**
		**orange1.1g000013m**	**AT1G55860.2**	**Ubiquitin-protein ligase 1 (E3 ubiquitin-protein ligase UPL1)**	**Protein ubiquitination**	2.82**
		**orange1.1g029528m**	**AT5G01520.1**	**RING/U-box superfamily protein**	**Protein ubiquitination**	4.05*
		**orange1.1g029508m**	**AT1G22360.1**	**UDP-glucosyl transferase 85A2**	**Flavonoid biosynthetic process**	1.99**
miR3438	−9.31063262**	orange1.1g000163m	AT1G55325.2	RNA polymerase II transcription mediators		2.29
miR1151	−10.44661802**	**orange1.1g018149m**	**AT5G49610.1**	**F-box family protein**	**Protein ubiquitination**	1.82*
		**orange1.1g018125m**	**AT5G49610.1**	**F-box family protein**	**Protein ubiquitination**	2.69*
		**orange1.1g023739m**	**AT2G41870.1**	**Remorin family protein**		4.00**
		**orange1.1g027436m**	**AT2G41870.1**	**Remorin family protein**		2.11**
		**orange1.1g023033m**	**AT2G36690.1**	**2-oxoglutarate (2OG) and Fe(II)-dependent oxygenase superfamily protein**	**Oxidoreductase**	2.03*
		**orange1.1g026453m**	**AT1G17020.1**	**Senescence-related gene 1**	**Oxidoreductase**	2.31**
		**orange1.1g020233m**	**AT2G36690.1**	**2-oxoglutarate (2OG) and Fe(II)-dependent oxygenase superfamily protein**		3.73*
		orange1.1g037473m	AT5G07480.1	KAR-UP oxidoreductase 1	Oxidoreductase	1.92
miR6219	−11.91465055**	**orange1.1g010903m**	**AT5G15130.1**	**WRKY DNA-binding protein 72**	**Transcription factor**	1.65**
miR7708	−8.18079862**	**orange1.1g023136m**	**AT1G06890.1**	**Nodulin MtN21 /EamA-like transporter family protein**		2.14**
miR780	−12.80878923**	**orange1.1g044623m**	**AT5G17230.2**	**Phytoene synthase**	**Carotenoid biosynthesis**	2.15*
		orange1.1g030826m	AT2G26560.1	Phospholipase A 2A	Lipid degradation	ND
		**orange1.1g004573m**	**AT4G27220.1**	**NB-ARC domain-containing disease resistance protein**	**Disease resistance**	1.58**
miR160	10.33203655**	orange1.1g005482m	AT4G30080.1	Auxin response factor 16	Auxin signaling pathway	4.54*
		orange1.1g004896m	AT2G28350.1	Auxin response factor 10	Auxin signaling pathway	22.94**
		orange1.1g005075m	AT4G30080.1	Auxin response factor 16	Auxin signaling pathway	4.01**
		orange1.1g008078m	AT1G77850.1	Auxin response factor 17	Auxin signaling pathway	2.90*

The relative changes of target genes are the ratio of Mg-deficient to -sufficient roots. The value for relative change of target gene was a mean of three biological replicates with two technical replicates; Target genes that had the expected changes in mRNA levels were marked in bold; * and ** indicate a significant difference at *P* < 0.05 and *P* < 0.01, respectively. *ND*, not detected

### Prediction and GO analysis of targets for Mg-deficiency-responsive miRNAs

**Fig. 4:**
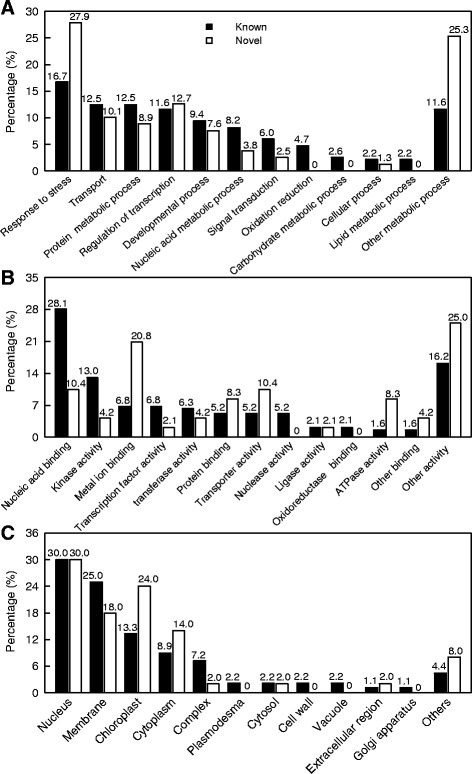
GO categories of the predicted target genes for 46 (15) Mg-deficiency-responsive known (novel) miRNAs in *Citrus sinensis* roots. MiRNAs target genes were grouped based on biological process (**a**), molecular function (**b**) and cellular component (**c**)

Here, we predicted 239 and 130 target genes from the 46 known and 15 novel Mg-deficiency-responsive-miRNAs, respectively (Additional files 7 and 8). As shown in Fig. 4a, the targets for known (novel) Mg-deficiency-responsive miRNAs were associated with 12 (nine) biological processes. The most abundant three GO terms were response to stress, transport and protein process for known miRNA targets and response to stress, regulation of transcription and transport for novel miRNA targets, respectively. On the basis of the molecular function, the highest percentages of three groups for known and novel miRNA targets were nucleic acid binding, other activity and kinase activity, and other activity, metal ion binding and transporter activity, respectively (Fig. 4b). As shown in Fig. 4c, the targets for known (novel) Mg-deficiency-responsive miRNAs were related to 12 (eight) cellular components. The most abundant component for known and novel miRNAs was nucleus.

### Validation of target genes by qRT-PCR

As shown in Table 2, 105 targets for 11 up- and 16 down-regulated miRNAs were validated by qRT-PCR. As expected, we found that 65 (61.9%) targets and their corresponding *miRNAs* displayed opposite trends in expression profiles in Mg-deprived roots, suggesting that miRNAs might play a role in gene regulation by cleaving mRNAs. However, 34 (32.4%) targets displayed the same expression profiles with their corresponding *miRNAs* in Mg-deficient roots or were not significantly affected by Mg-deficiency. The remaining 6 (0.06%) targets were not detected in roots. It is worth mentioning that 4 target genes (i.e., range1.1g005482m, orange1.1g004896m, orange1.1g005075m and orange1.1g008078m) belonging to auxin responsive factor (ARF) family have been validated by us in *C. sinensis* [42], suggesting that the target prediction was accurate.

## Discussion

Little is known about the possible roles of miRNAs in plant Mg homeostasis [36, 43]. Here, we first investigated the Mg-deficiency-induced alterations of miRNA profiles in *Citrus* roots and obtained 101 up- and 69 down-regulated miRNAs (Additional files 3 and 6), demonstrating that miRNAs might be involved in Mg-deficiency responses. We obtained similar amount of miRNAs (71 miRNAs) with decreased expression, but less amount of miRNAs (75 miRNAs) with increased expression from Mg-deficient *C. sinensis* leaves compared with Mg-deficient *C. sinensis* roots [36]. Moreover, most of these miRNAs were isolated only from Mg-deprived roots or leaves, only 30 Mg-deficiency-responsive miRNAs were shared by the two. Among the 30 overlapping miRNAs, only 15 miRNAs displayed similar expression trends in Mg-deprived roots and leaves (Table 3). Thus, great differences existed in Mg-deficiency-induced alterations of miRNA profiles between roots and leaves. This agrees with our report that the physiological and biochemical responses to long-term Mg-deficiency greatly differed between *C. sinensis* roots and leaves [7].

**Table 3:** List of Mg-deficiency-responsive known miRNAs shared by both *C. sinensis* roots and leaves

MiRNA	Fold change	
	Leaves	Roots
miR6108	−10.67424131**	13.767751**
miR1851	12.01270484**	11.47852696**
miR917	11.96734516**	11.42532082**
miR5525	−10.87750643**	10.81431809**
miR158	−6.05735341**	9.23711361**
miR1077	11.84568538**	8.75152564**
miR779	−8.10749886**	8.40390779**
miR1168	6.26630003**	7.67556689**
miR7730	12.71420043**	7.66997733**
miR1512	4.98806257**	7.2149053**
miR1440	5.4977661**	6.69472111**
miR5782	−10.28908522**	5.9080234**
miR3520	4.1203203**	5.61646655**
miR5830	−4.18762998**	5.11294928**
miR395	10.30345436**	4.61153607**
miR5210	−3.03780053**	4.38945452**
miR3437	−4.38720917**	3.97352136**
miR5304	4.5270768**	3.11469877**
miR7485	2.08344945**	1.63183983**
miR5818	−4.95063483**	1.50697816**
miR1222	−2.88894979**	−13.23073355**
miR6425	−5.81634371**	−10.19869057**
miR3438	7.67399457**	−9.31063262**
miR7708	−10.0710064**	−8.18079862**
miR5290	4.73213099**	−7.59506455**
miR6247	−9.56671537**	−6.67041637**
miR2616	6.25578869**	−4.06995485**
miR5286	6.7663942**	−3.78538232**
miR6426	4.30631516**	−1.65860955**
miR812	7.83035956**	−1.64980601**

Data from Additional file 3 and Ma et al. [36]; **indicates a significant difference at *P* < 0.01

We observed that *miR158* was induced in Mg-deprived roots (Table 2). Similar results have been obtained on Mg-deficient *C. sinensis* leaves [36], P-deficient tomato roots and leaves [22], and B-starved *C. sinensis* roots and leaves [17, 18]. As expected, its target gene *SPFH* (stomatins, prohibitins, flotillins and HflK/C)*/Band 7/PHB domain-containing membrane-associated protein family* (AT5G62740) was repressed in Mg-deprived roots. Wang et al. [44] found that *Arabidopsis phb3–3* mutants were less sensitive to salt-stress-induced inhibition of primary root growth. Thus, the down-regulation of AT5G62740 might contribute to *Citrus* Mg-deficiency tolerance via alleviating Mg-deficiency-induced inhibition of root growth (Fig. 1a). Gehl et al. [45] observed that the basal tissue respiration rate in *stomatin-like protein 1* (*slp1*) knockout *Arabidopsis* roots was reduced by 30% compared with wild-type. In addition, *miR2919* expression was induced and its target: *phosphoenolpyruvate carboxylase 3* (*PEPC3*) was inhibited in Mg-deprived *C. sinensis* roots (Table 2). Therefore, root respiration might be decreased in Mg-starved *C. sinensis* roots. This agrees with our reports that the abundances of pyruvate decarboxylase (gi|255,579,310) and phosphoglycerate kinase (gi|332,195,235) in glycolysis and the activities of key enzymes in glycolysis and tricarboxylic acid (TCA) cycle were reduced in Mg-deprived *C. sinensis* roots accompanied by decreased accumulation of carbohydrates and lower respiration [7, 12].

Both root *miR6278* and its targets: *NB-ARC domain-containing disease resistance protein* involved in disease resistance and *DnaJ/Hsp40 cysteine-rich domain superfamily protein* (AT3G14470.1) were induced by Mg-deficiency. In addition, *NB-ARC domain-containing disease resistance protein* (AT4G27220.1) targeted by miR780 was induced in Mg-depprived roots (Table 2). Similarly, the abundances of Grp94 (HSP; gi|23,477,636) and disease resistance protein (gi|227,438,123) was increased in Mg-starved *C. sinensis* roots [12]. Thus, disease resistance might be elevated in these roots with increased levels of Ca and K [46], which contribute to plant disease resistance [47, 48].

MiR414 mainly targets transcriptional regulators including MYB, bZIP family transcription factors, WRKY and scarecrow and might have key roles in plant growth and development [49]. As expected, *miR414* was up-regulated and its target gene: *poly(A) polymerase 1* was inhibited in Mg-deprived roots (Table 2). This was also supported by our reports that the abundances of transcription factor homolog BTF3-like protein (gi|33,945,882), spliceosome RNA helicase BAT1 (gi|226,528,292) and RNA polymerase β chain (gi|90,403,817) were lowered in Mg-deficient *C. sinensis* roots [12].

We found that *miR1847* was inhibited in Mg-deprived roots (Table 2). This agrees with the results obtained on B-deficient roots [17] and disagrees with the data obtained on B-deficient leaves [18]. As expected, its target genes: *ribosomal protein S3 family proteins* were up-regulated in these roots. In addition, *VALRS* targeted by miR6485 were induced or was little affected in Mg-starved roots (Table 2). Thus, protein biosynthesis might not be lowered in Mg-starved roots, as shown by unchanged concentration of total soluble proteins in Mg-deprived *C. sinensis* roots [7, 12]. Also, the reduced dilution due to the decrease in root DW (Fig. 1a) might account for the unchanged protein level.

*MiR5176* was induced in Mg-deprived roots (Table 2), as found on B-starved *C. sinensis* roots [17]. DNA mismatch repair (MMR) system is required for the correction of DNA biosynthetic errors [50]. MUTL-homologue 1 (MLH1) participates in DNA MMR, correcting DNA damage and insertion-deletion loops arising from DNA replication [51]. *MLH1* targeted by miR5176 were induced rather than inhibited in Mg-deprived roots (Table 2). Thus, MMR system might be up-regulated in these roots, thus enhancing *Citrus* Mg-deficiency tolerance via correcting DNA biosynthetic errors. Similarly, *DNA repair and meiosis protein* (*Mre11*) targeted by miR5261 was induced in Mg-deprived roots (Table 2).

Plant leucine-rich repeat receptor-like kinase proteins play crucial roles in abiotic stresses [52]. *MiR5198* and its target gene: *leucine-rich receptor-like protein kinase* (*LR-RLK*) *family protein* were repressed and induced in Mg-starved roots, respectively (Table 2). Similarly, *leucine-rich repeat receptor-like protein kinase* (ACN59310.1) was up-regulated in Mg-starved *C. reticulata* roots [13]. Thus, *miR5198* might be involved in *Citrus* Mg-deficiency responses.

Root *miR780* was repressed by Mg-deficiency (Table 2), as found on B-starved *C. sinensis* roots [17]. As expected, its targets: *NB-ARC domain-containing disease resistance protein* and *phytoene synthase* (*PSY*) were up-regulated in Mg-starved roots (Table 2). Various transgenic plants over-expressing bacterial or plant gene encoding PSY, a major rate-limiting carotenoid (Car) enzyme, displayed increased Car level [53, 54]. Therefore, Car biosynthesis might be enhanced in Mg-starved *C. sinensis* roots, thus increasing their antioxidant ability.

We observed that *miR7121* and its target gene: *phosphatidic acid phosphatase* (*PAP2*) *family protein* was up- and down-regulated in Mg-starved roots, respectively (Table 2). Nakano et al. [55] demonstrated that the inhibition of *PAP2* expression or function conferred resistance to *Ralstonia solanacearum* via rapidly triggering plant defenses in *Nicotiana benthamiana*. Thus, the down-regulation of *PAP2 family protein* might contribute to plant disease-resistance.

As shown in Table 2, *miR6190* was down-regulated and its target genes [i.e., *purple acid phosphatase 26* (*PPAP26*), *Sec23/Sec24 protein transport family protein* and *ortholog of human splicing factor SC35*, also known as s*erine/arginine-rich (SR) splicing factor SC35*] were up-regulated in Mg-deficient roots. SR proteins are required for regulating alternative splicing. In higher plants, great alterations in alternative splicing due to various abiotic stresses demonstrate the roles of SR proteins in the adaptation to environmental stress [56]. Induction of acid phosphatases (APases) by P-starvation is a well-documented mechanism of plant P-deficiency tolerance. Hurley et al. [57] demonstrated that AtPPAP26 was the major contributor to P-deficiency-inducible APase activity. In addition, AtPPAP26 also showed alkaline peroxidase (POD) activity. Mg-deficiency-induced up-regulation of root *PPAP26* (Table 2) agrees with the report that *AtPPAP26* was induced in P-deficient *Arabidopsis* roots, shoots and suspension cells [58] because *C. sinensis* leaf, stem and root P levels were reduced by Mg-deficiency [46]. Coat protein complex II (COPII) vesicles play an essential role for the export of secretory cargo from the endoplasmic reticulum (ER) to the Golgi complex in all eukaryotes [59]. Mg-deficiency-induced up-regulation of root gene encoding Sec23/Sec24 protein transport family protein (Table 2), a subset of the COPII components, agrees with our report that the abundance of Sec23/Sec24 protein transport family protein was elevated in B-deficient roots [60].

In *Arabidopsis*, miRNA160 negatively regulates the repressor auxin response factor (ARF) family: *ARF17* [61], *ARF16* [62] and *ARF10* [63]. The repression of these genes by miR160 is required for seed germination and the normal development of roots, stems and leaves. Li et al. [64] demonstrated that soybean miR160a negatively regulated the progress of leaf senescence via repressing its targets: *ARF10*, *ARF16* and *ARF17*. We found that *miR160* was induced in Mg-deficient roots (Table 2), as obtained on P-starved *Lupinus albus* roots [65] and N-deficient maize roots [66]. Therefore, the induction of root *miR160* by Mg-deprivation might be an adaptive response. Unexpectedly, its targets: *ARF10*, *ARF16* and *ARF17* were up-regulated in Mg-deprived *C. sinensis* roots (Table 2). Endogenous target mimics (eTMs) can impede the interaction between miRNAs and their authentic targets via binding to miRNAs [67, 68]. Lin et al. [69] demonstrated that eTMs repressed miR160-mediated cleavage of *ARF10*, *ARF16* and *ARF17* during longan somatic embryogenesis. No negative correlations were observed among the levels of *miR160* and *ARF10*, *ARF16* and *ARF17* transcripts in longan vegetative and generative tissues. Thus, the correlations between *miR160* and its targets in *C. sinensis* roots can be explained in this way.

Root *miR6485* was repressed and its several target genes were up-regulated by Mg-deprivation (Table 2). Li et al. [70] reported that ARF7 (AT5G20730) is necessary for both auxin signaling and ethylene responses in *Arabidopsis* roots. Okushima et al. [71] observed that lateral root formation was badly damaged in *Arabidopsis arf7 arf19* double knockout mutant, concluding that ARFs directly activated LATERAL ORGAN BOUNDARIES DOMAIN/ASYMMETRIC LEAVES2-LIKE (LBD/ASL) genes, thus regulating lateral root formation. Thus, the induction of root *transcriptional factor B3 family protein/auxin-responsive factor AUX/IAA-related* by Mg-deficiency might play a part in Mg-deficiency tolerance via maintaining lateral root formation. Similarly, *SnRNA activating complex family protein* (*SDR2*), which is associated with auxin-activated signaling pathway, was induced in Mg-deprived roots (Table 2). Ohtani et al. [72] reported that *srd2* mutation repressed the expression of PIN-FORMED proteins, which might account for the failure to generate an auxin gradient, thus leading to different abnormalities in root morphogenesis in *Arabidopsis* mutant. Nuclear pore complex protein NUP88 is necessary for systemic acquired resistance and R protein-mediated defense [73]. The induction of root *NUP88* by Mg-deficiency (Table 2) agrees with the above inference that that disease-resistance was improved in Mg-deficient roots.

Root *miR1044* was repressed and its target genes [i.e., *NF-X-like 1* (*NFXL1*), *POD superfamily protein* and *ATPase, AAA-type, CDC48 protein*] were induced by Mg-deprivation except for *integrase-type DNA-binding superfamily protein* (Table 2). Lisso et al. [74] observed that *AtNFXL1* was induced in roots under salt and osmotic stress, and that both *AtNFXL1*-antisense plants and *atnfxl1–1* knock-out mutants had lower growth and survival rates than wild-type plants when exposed to salt or osmotic stress. CDC48, a member of AAA-ATPase family proteins that provides energy for plant development via regulating ATPase, is required for plant cell division, expansion and differentiation [75]. Wang et al. [76] suggested that the induction of *PpCDC48II* by low temperature played a key role in cold-induced freezing tolerance of *Physcomitrella patens* cells.

*MiR5261* and its target genes were repressed and induced in Mg-starved roots, respectively (Table 2). The induction of root *RNA binding (RRM/RBD/RNP motifs) family protein* by Mg-deficiency agrees with our report that the abundance of RNA binding (RRM/RBD/RNP motifs) family protein was elevated in B-deficient roots [60]. A typical mitogen-activated protein kinase (MAPK) cascade is composed of three sequentially activated protein kinases, namely MAPK, MAPK kinase (MAPKK) and MAPKK kinase (MAPKKK). Stress-tolerance of some plants such as *Arabidopsis*, tobacco and cereals has been enhanced by genetically altering the abundances and/or the activities of some MAPK components [77, 78]. The induction of root *protein phosphatase 2A (PP2A) regulatory B subunit family protein* by Mg-deficiency (Table 2) agrees with the report that wheat root *PP2AbB"-α* was up-regulated when exposed to various abiotic stresses. Transgenic wheat lines over-expressing *TaPP2AbB"-α* displayed better lateral root development, especially under NaCl and mannitol stresses [79].

Ubiquitination-proteasomal pathway has been shown to function in plant senescence and in stress response by facilitating the degradation of bulk proteins for N recycling [80]. Transgenic tobacco lines over-expressing a maize gene encoding E3 ubiquitin ligase (UPL) displayed increased drought tolerance accompanied by higher activities of superoxide dismutase (SOD) and catalase, more accumulation of proline and less accumulation of malondialdehyde (MDA) and ROS when exposed to drought stress [81]. Over-expression of *TaFBA1* encoding F-box protein conferred drought and oxidative stress tolerance in tobacco plants via up-regulating the activities of SOD, catalase, ascorbate peroxidase (APX) and POD, and lowering the levels of ROS and MDA [82, 83]. Thus, up-regulation of *UPL1* and *RING/U-box superfamily protein* targeted by miR5261, *NFXL1* targeted by miR1044, *F-box family protein* targeted by miR1151 and *proteasome subunit beta type-6* (*PBA1*) targeted by miR5029 in Mg-starved roots (Table 2) might confer stress-tolerance, thus contributing to Mg-deficiency tolerance in *Citrus* plants. Similarly, the expression levels of *UPL5* (XP 003594229.1) and *F-box family protein* (XP 003612153.1) in *C. reticulata* roots [13] and the abundances of putative proteasome subunit alpha type (gi|255,584,432) in *C. sinensis* roots [12] were elevated by Mg-deficiency.

## Conclusions

**Fig. 5:**
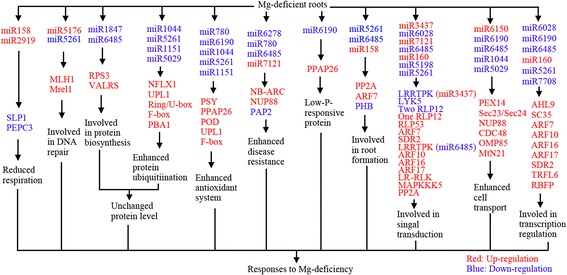
A potential scheme for responses of *C. sinensis* roots miRNAs to Mg-deficiency. LRRTPK: Leucine-rich repeat transmembrane protein kinase; LYK5: LysM-containing receptor-like kinase 5; PEX14: Peroxin 14; RBFP: RNA binding (RRM/RBD/RNP motifs) family protein; RLP: Receptor like protein; RPS3: Ribosomal protein S3 family protein; WRKY72: WRKY DNA-binding protein 72

For the first time, we used Illumina sequencing to identify 71 known and 30 novel miRNAs with increased expressed, and 54 known and 15 novel miRNAs with decreased expression in Mg-deficient *C. sinensis* roots, demonstrating that miRNAs might be involved in *Citrus* Mg-deficiency tolerance. Through integrating our findings with the previous data, we put forward a potential scheme for the responses of miRNAs to Mg-deficiency in *Citrus* roots (Fig. 5). Here, we obtained several novel Mg-deficiency-responsive miRNAs (i.e., miR5261, miR158, miR6190, miR6485, miR1151 and miR1044) possibly responsible for *Citrus* Mg-deficiency tolerance. Our findings results not only increased our knowledge on the functions of plant miRNAs under nutrient deficiencies, but also established foundation to improve Mg-deficiency tolerance via manipulating the actions of miRNAs.

## Methods

### *Citrus sinensis* Seedling culture and long-term Mg-deficient treatments

Seedling culture and long-term Mg-deficient treatments were carried out as described previously [12]. In short, 15-week-old ‘Xuegan’ [*Citrus sinensis* (L.) Osbeck] seedlings, which were grown in 6 L pots (two seedlings per pot) filled with clean river sand in a greenhouse under natural photoperiod at Fujian Agriculture and Forestry University, Fuzhou, were supplied every other day until dripping with nutrient solution at a Mg concentration of 0 mM (Mg-deficiency) or 1 mM (Mg-sufficiency, control) from MgSO_4_. S at the nutrient solution was kept at a constant level by adding equivalent moles of Na_2_SO_4_ in replace of MgSO_4._ After 16 weeks, ~ 5-mm-long root apices from new white fibrous roots were harvested and immediately frozen in liquid N_2_, then stored at −80 °C until extraction. The seedlings not being sampled were used for the measurements of root DW, leaf and root Mg.

### Root DW and root and leaf Mg

For each treatment, roots from nine seedlings (one seedling per pot) were taken. Root DW was measured after being dried at 70 °C to a constant weight (~ 48 h).

Fibrous roots and ~7-week-old leaves (midribs and petioles removed) were harvested and then dried at 70 °C to a constant weight. Dried roots and leaves were ground to pass a 40 mesh sieve, finally digested with 1 N HCl [84]. Mg concentration in the solution was measured by atomic absorption spectroscopy.

### Root sRNAs library construction, high-throughput sequencing, annotation and miRNA identification

Equal amounts of frozen root apices from five seedlings (one seedling per pot) were pooled as a biological replicate. There was one biological replicate for each treatment. Approximately 0.1 g mixed frozen Mg-deficient or control root apices were used to extract total RNA with TRIzol reagent (Invitrogen, Carlsbad, CA). Construction of sRNA libraries was performed as described by Lu et al. [17]. Illumina sequencing was carried out with a Solexa sequencer at the Beijing Genomics Institute (BGI), Shenzhen, China.

Both sRNA annotation and miRNA identification were made as described previously [17, 18]. After raw data being analyzed with a software developed by BGI, clean reads were then utilized to assay length distribution. Finally, the clear reads were mapped to *C. sinensis* genome (JGIversion 1.1, http://phytozome.jgi.doe.gov/pz/portal.html#!info?alias=Org_Csinensis) using SOAP, only perfectly mapped sequences were retained and analyzed further. rRNAs, tRNAs, snRNAs and snoRNAs were removed from the sRNAs sequences through BLASTn search using NCBI Genebank database (http://www.ncbi.nlm.nih.gov/blast/Blast.cgi/) and Rfam (12.0) database (http://www.sanger.ac.uk/resources/databases/rfam.html) (*e* = 0.01). The remaining sequences were aligned with known plant miRNAs from miRBase 21 (http://www.mirbase.org/). Only the perfectly matched sequences were considered to be conserved miRNAs. Reads not being annotated were used for the prediction of novel miRNAs using Mireap (http://sourceforge.net/projects/mireap/), a software developed by BGI. Also, both DNAMAN 8 (http://www.lynnon.com/pc/framepc.html) and MTide (http://bis.zju.edu.cn/MTide) [85] were used for the prediction of novel miRNAs. Only these miRNA candidates being simultaneously predicted by the three softwares were regarded to be novel miRNAs.

### Differentially expressed miRNAs and target prediction

Both the fold change between Mg-deficient and -sufficient roots and the *P*-value were calculated from the normalized expression of TPM [86]. A miRNA was regarded to be differentially expressed when it had both a *P*-value <0.01 and a log2-fold change >1.5 [17]. Target prediction of miRNAs was carried out by RNAhybrid according to the rules proposed by Schwab et al. [87] and Allen et al. [88].

### Categories of the potential targets predicted from Mg-deficiency-responsive miRNAs

All target genes predicted from Mg-deficiency-responsive miRNAs were mapped to GO terms in the database (http://www.geneontology.org/), and gene numbers for each term was calculated. All these targets were grouped into three categories: biological process, molecular function, cellular component [17].

### Validation of Mg-deficiency-responsive miRNAs by stem-loop qRT-PCR and of target genes by qRT-PCR

Stem-loop qRT-PCR analysis of miRNAs was carried out as described previously [18]. Stem-loop primers for reverse transcription and primers for qRT-PCR were summarized in Additional file 9. qRT-PCR analysis of target genes was carried out with an ABI 7500 Real Time System as described by Lu et al. [17]. The sequences of the F and R primers used were given in Additional file 10. Equal amounts of frozen root apices from five seedlings (one seedling per pot) were pooled as a biological replicate. For each treatment, there were three biological replicates and two technical replicates. Relative miRNA expression was calculated using ddCt algorithm. *Actin* (AEK97331.1) was used as an internal standard and the roots from Mg-sufficient seedlings were used as reference sample, which was set to 1.

### Experimental design and data analysis

For each treatment, there were 20 pot seedlings in a completely randomized design. Experiments were carried out with 3 replicates except for high-throughput sequencing (*n* = 1), root and leaf Mg (*n* = 5), and root DW (*n* = 9). Unpaired *t*-test was performed for the significant test between two means (Mg-sufficiency and -deficiency).

## Additional files

Additional file 1: List of known miRNAs in *C. sinensis* roots. (DOC 995 kb)

Additional file 2: List of known miRNAs in *C. sinensis* roots after removing these miRNAs with normalized read-count less than 10 TPM in the two miRNA libraries constructed from Mg-sufficient and -deficient roots. (DOC 447 kb)

Additional file 3: List of Mg-deficiency-responsive known miRNAs in *C. sinensis* roots. (DOC 205 kb)

Additional file 4: List of novel miRNAs in *C. sinensis* roots. (DOC 288 kb)

Additional file 5: List of novel miRNAs in *C. sinensis* roots after removing these miRNAs with normalized read-count less than 10 TPM in the two miRNA libraries constructed from Mg-sufficient and -deficient roots. (DOC 160 kb)

Additional file 6: List of Mg-deficiency-responsive novel miRNAs in *C. sinensis* roots. (DOC 105 kb)

Additional file 7: List of target genes for parts of known miRNAs in *C. sinensis* roots. (DOC 262 kb)

Additional file 8: List of target genes for parts of novel miRNAs in *C. sinensis* roots. (DOCX 25 kb)

Additional file 9: Stem loop primer sequences for qRT-PCR analysis of miRNAs. (DOCX 17 kb)

Additional file 10: Specific primer pairs used for qRT-PCR expression analysis of selected miRNA target genes. (DOCX 31 kb)

## Abbreviations

AHL9: AT-hook motif nuclear-localized protein 9
APase: Acid phosphatase
APX: Ascorbate peroxidase
ARF: Auxin responsive factor
Car: Carotenoid
CDC48: A member of AAA-ATPase family proteins
COPII: Coat protein complex II
ER: endoplasmic reticulum
eTMs: Endogenous target mimics
LR-RLK: Leucine-rich receptor-like protein kinase
MAPK: Mitogen-activated protein kinase
MAPKK: MAPK kinase
MAPKKK: MAPKK kinase
MDA: Malondialdehyde
Mg: Magnesium
MLH1: MUTL-homologue 1
MMR: DNA mismatch repair
NUP88: Nuclear pore complex protein
NFXL1: NF-X-like 1
NLA: Nitrogen limitation adaptation
PAP: Phosphatidic acid phosphatase
PBA1: Proteasome subunit beta type-6
PEPC: Phosphoenolpyruvate carboxylase
PHB: Prohibitin
PLA2A: Phospholipase A 2A
POD: Peroxidase
PP2A: Protein phosphatase 2A
PPAP: Purple acid phosphatase
PSY: Phytoene synthase
ROS: Reactive oxygen species
SC35: Ortholog of human splicing factor
SDR2: SnRNA activating complex family protein
SLP1: Stomatin-like protein 1
SOD: Superoxide dismutase
SPFH: Stomatins, prohibitins, flotillins and HflK/C
TPM: Transcript per million
UCB24: Ubiquitin-conjugating enzyme E2 24
UPL1: Ubiquitin-protein ligase 1 (E3 ubiquitin-protein ligase UPL1)
VALRS: Valyl-tRNA synthetase / valine-tRNA ligase.
