# Supplementary material for: MicroRNA-mediated responses to long-term magnesium-deficiency in Citrus sinensis roots revealed by Illumina sequencing
Source: BMC Genomics. 2017 Aug 24;18:657. doi: 10.1186/s12864-017-3999-5 (PMC5571589; doi:10.1186/s12864-017-3999-5)
Supplement: Supplementary file 1 — List of known miRNAs in C. sinensis roots. (DOC 995 kb) [file 12864_2017_3999_MOESM1_ESM.doc]

**Additional file 1** List of known miRNAs in *C. sinensis* roots.

| miRNA | Sequence | Expressed | | Normalized read count | | Fold change |
| --- | --- | --- | --- | --- | --- | --- |
|  |  | Control | Mg-  deficiency | Control | Mg-  deficiency |  |
| **Up-regulated miRNAs** | |  |  |  |  |  |
| miR6108 | AGGTGAGAAGGGTGATCT | 0 | 2835 | 0.01 | 139.478 | 13.767751** |
| miR8032 | AGTGTGGAGTGGGAGTGTGAGTAGG | 0 | 2618 | 0.01 | 128.802 | 13.65286737** |
| miR5243 | TGGGCAGAGTATTCGGTGAGC | 0 | 2542 | 0.01 | 125.063 | 13.61036626** |
| miR3981 | AGTATTCAAGATCGTCTCAT | 0 | 1448 | 0.01 | 71.2396 | 12.7984637** |
| miR2870 | ACTAACAGTTTGGTGGACGACAAA | 0 | 881 | 0.01 | 43.344 | 12.08161658** |
| miR8128 | TCAGCGGGGAAAGACTAATCG | 0 | 870 | 0.01 | 42.8028 | 12.06348946** |
| miR5513 | AAACAAAGGAAACAGACA | 0 | 703 | 0.01 | 34.5866 | 11.75599748** |
| miR5141 | AGACCAGACGTGATGAGCAGATAA | 0 | 683 | 0.01 | 33.6027 | 11.71436144** |
| miR1851 | TATGGGATGGCATTTGGC | 0 | 580 | 0.01 | 28.5352 | 11.47852696** |
| miR917 | TTTGCACGGTTATTTTTGAA | 0 | 559 | 0.01 | 27.502 | 11.42532082** |
| miR5062 | TGAACCTCTGGAGAAGAAGCCCCT | 0 | 425 | 0.01 | 20.9094 | 11.02993595** |
| miR7725 | AAACGAGATGGGACGAGAT | 0 | 407 | 0.01 | 20.0238 | 10.96750007** |
| miR8138 | TAAAGATGGGAACAAAACAA | 0 | 404 | 0.01 | 19.8762 | 10.95682625** |
| miR5338 | TGAAGCTCAGTTGGTAGGTTT | 0 | 397 | 0.01 | 19.5318 | 10.9316092** |
| miR5525 | TCAATCCTTGTGGAGACGATCTGA | 0 | 366 | 0.01 | 18.0067 | 10.81431809** |
| miR1078 | CTTGATTGATTGTTGGAT | 1 | 1594 | 0.0459 | 78.4226 | 10.7385596** |
| miR2878 | TACATGTTAAAATTTTGTAGTGAT | 0 | 328 | 0.01 | 16.1371 | 10.65616562** |
| miR8130 | GGGTTCAATTGTGGAAGGCT | 0 | 308 | 0.01 | 15.1532 | 10.56540677** |
| miR160 | TGCCTGGCTCCCTGTATGCCA | 0 | 262 | 0.01 | 12.89 | 10.33203655** |
| miR2631 | TGACACCTACGATGGCACACC | 0 | 267 | 0.01 | 13.136 | 10.35931032** |
| miR1853 | TAATTCGGGTTATGTTCGGATTGT | 0 | 258 | 0.01 | 12.6932 | 10.30984011** |
| miR2876 | AATGGTGGCTGCGACTGTTTA | 0 | 250 | 0.01 | 12.2997 | 10.26440741** |
| miR1432 | ACATGGAGAGGACACCGAC | 0 | 231 | 0.01 | 11.3649 | 10.15036927** |
| miR4410 | TATGTTGTCCGTCATGATCGTGAA | 0 | 226 | 0.01 | 11.1189 | 10.11879835** |
| miR7488 | TTTTGATGTAGCAGGGGAAACAA | 0 | 207 | 0.01 | 10.1841 | 9.99210278** |
| miR7722 | GAGGGCATCGGGATGGGG | 0 | 201 | 0.01 | 9.8889 | 9.94966624** |
| miR6213 | CCAGATTGTAAGATGGTC | 0 | 198 | 0.01 | 9.7413 | 9.92797051** |
| miR6463 | TGGATGACATGTTGGTAGCAGACC | 0 | 179 | 0.01 | 8.8066 | 9.78244133** |
| miR5182 | TGATTAGTTGGAACACGTC | 0 | 156 | 0.01 | 7.675 | 9.58402294** |
| miR6154 | TGGGATGTCGTGGAGAAAGGC | 0 | 156 | 0.01 | 7.675 | 9.58402294** |
| miR5277 | AGGTCTGTTTCTGAAGATGAAGGA | 0 | 149 | 0.01 | 7.3306 | 9.51778748** |
| miR6461 | TAGCTGGAGTTGATGGATC | 0 | 128 | 0.01 | 6.2974 | 9.2986125** |
| miR158 | TCTTAAATGTAGACAAAGCA | 1 | 563 | 0.0459 | 27.6988 | 9.23711361** |
| miR1067 | ACAGACTGAAGTTGTTGCA | 0 | 116 | 0.01 | 5.707 | 9.15658875** |
| miR2675 | GAGGCGATTTGTAGGGATT | 0 | 113 | 0.01 | 5.5594 | 9.11878538** |
| miR2088 | ATGCACTAGATTACCTTGAC | 0 | 106 | 0.01 | 5.2151 | 9.02655111** |
| miR6150 | CTTGTTTGATGGTATTTGCT | 9 | 4179 | 0.4132 | 205.601 | 8.95879131** |
| miR7760 | TCGGCTTTGGTCGGAGTGATGGGC | 0 | 101 | 0.01 | 4.9691 | 8.95684077** |
| miR1045 | AAGTGATGGCATTTTGACTT | 0 | 100 | 0.01 | 4.9199 | 8.94248518** |
| miR6278 | TCATTGTACACAAGCTGAG | 5 | 2162 | 0.2295 | 106.367 | 8.85634619** |
| miR5056 | AGGAAGAACAGGTAATAA | 0 | 91 | 0.01 | 4.4771 | 8.80642073** |
| miR7494 | AGCTATAGTAGACAAGTTTAACAA | 0 | 91 | 0.01 | 4.4771 | 8.80642073** |
| miR5037 | ATGAGAACTTTGAGGCCG | 0 | 89 | 0.01 | 4.3787 | 8.7743588** |
| miR1077 | TTGAAGTGTTCGGATCGCGGC | 78 | 31368 | 3.5807 | 1543.26 | 8.75152564** |
| miR5768 | AATACAATATGATCTTCGGATACC | 0 | 86 | 0.01 | 4.2311 | 8.72488897** |
| miR901 | GGTTAGAAGTGGCGGCTGTTA | 0 | 82 | 0.01 | 4.0343 | 8.65617456** |
| miR8006 | TACTTTTTGGAACTGAAGGGGCAC | 0 | 80 | 0.01 | 3.9359 | 8.62054976** |
| miR5230 | CAAATCTTGGATCGAACTGGA | 0 | 77 | 0.01 | 3.7883 | 8.56540677** |
| miR7837 | TGGGTGGGAGGTCGAGCT | 0 | 76 | 0.01 | 3.7391 | 8.54654725** |
| miR5051 | TTTGGACATTGACTGGGA | 0 | 74 | 0.01 | 3.6407 | 8.50807205** |
| miR7816 | AATTTGTTTGATCACTGTA | 0 | 74 | 0.01 | 3.6407 | 8.50807205** |
| miR2626 | AACGTTCGGCTGACTTTAGGGTTT | 0 | 73 | 0.01 | 3.5915 | 8.48844271** |
| miR779 | TCTGCTCATAGATTGTCTGCTCAT | 1 | 316 | 0.0459 | 15.5468 | 8.40390779** |
| miR1156 | TTGGACCCTCGAAGTACGTGA | 0 | 66 | 0.01 | 3.2471 | 8.34300801** |
| miR7983 | TAAAGCTTTAGGACATTGGTGA | 0 | 65 | 0.01 | 3.1979 | 8.32098102** |
| miR2597 | TTTGGCTTCTTCGTCGGGTTTGA | 0 | 64 | 0.01 | 3.1487 | 8.2986125** |
| miR2868 | TTGGTTTTGTGTGTGCAT | 0 | 61 | 0.01 | 3.0011 | 8.22934758** |
| miR1153 | AGTGCGTGCTGGCAACTACGG | 0 | 58 | 0.01 | 2.8535 | 8.15658875** |
| miR5084 | AACAGCTACTGCAGAGGTATCCTG | 0 | 57 | 0.01 | 2.8043 | 8.13149688** |
| miR3625 | AGGAGATCTACTGGAAGC | 0 | 54 | 0.01 | 2.6567 | 8.05349151** |
| miR7726 | TTGATGATGTTAGACGCGCGGT | 0 | 54 | 0.01 | 2.6567 | 8.05349151** |
| miR5777 | CTCAGAATTGTTGGATGCAC | 0 | 49 | 0.01 | 2.4107 | 7.91330832** |
| miR7500 | GATCGGTTTTCGGGTTAATCGGG | 0 | 49 | 0.01 | 2.4107 | 7.91330832** |
| miR5824 | AGTCAAGATAAGAGAGTAAGGCGT | 0 | 47 | 0.01 | 2.3123 | 7.85318478** |
| miR6176 | GAACTGTTAGATGCGGCCGGCGT | 0 | 47 | 0.01 | 2.3123 | 7.85318478** |
| miR5270 | GTAGGAGGAGTAGTTGAGGTGAT | 0 | 45 | 0.01 | 2.2139 | 7.79044625** |
| miR7727 | TCAAGATCGATTGGAATAAGGGC | 1 | 203 | 0.0459 | 9.9873 | 7.76545674** |
| miR5555 | ATAAGGAGAATAATATGCACTTTG | 0 | 43 | 0.01 | 2.1155 | 7.72485488** |
| miR1168 | TGTGGACAAGGCCAAGGAA | 13 | 2480 | 0.5968 | 122.013 | 7.67556689** |
| miR7730 | ATGAACACGACACGATTGAAGTTAT | 4 | 760 | 0.1836 | 37.3909 | 7.66997733** |
| miR6107 | AAGCGGACAATATCTTGGTAGGC | 0 | 41 | 0.01 | 2.0171 | 7.6561388** |
| miR6279 | AAGCAAGAATTCTCAAGACC | 0 | 39 | 0.01 | 1.9187 | 7.58398535** |
| miR6442 | TGAACGGCTTGAAGTGAC | 0 | 39 | 0.01 | 1.9187 | 7.58398535** |
| miR435 | TTATCCGGTTGGAGATTGC | 0 | 37 | 0.01 | 1.8203 | 7.50803243** |
| miR7733 | GCTGACGTTGGCGAGGCGAGAAC | 0 | 36 | 0.01 | 1.7712 | 7.46858332** |
| miR5171 | ACTTAATATTGGACGGGGA | 0 | 34 | 0.01 | 1.6728 | 7.38612116** |
| miR7785 | GTAGGTGGGTAGAGAGAGAAGGC | 3 | 457 | 0.1377 | 22.4838 | 7.35121352** |
| miR3631 | ATGTTGGATGATGGCAAAA | 1 | 143 | 0.0459 | 7.0354 | 7.25999449** |
| miR5381 | AAGATCTGTGGCAGCCCAA | 0 | 31 | 0.01 | 1.5252 | 7.25285463** |
| miR7997 | ATTTGATCGGGACCTATCAAAAAT | 0 | 31 | 0.01 | 1.5252 | 7.25285463** |
| miR1512 | ATAAATGGAAATTGGGATCAATGA | 5 | 693 | 0.2295 | 34.0946 | 7.2149053** |
| miR5760 | TGTTATTAAGGATGATTTGTAGGA | 0 | 30 | 0.01 | 1.476 | 7.20554891** |
| miR5504 | AGTGGACGGGAGGAATGGCAATG | 0 | 28 | 0.01 | 1.3776 | 7.10601324** |
| miR4345 | AATAGACGGAACTTACTAAGAT | 52 | 6630 | 2.3871 | 326.187 | 7.09429559** |
| miR2641 | TTTGATCCTTGTAGTTGAT | 0 | 27 | 0.01 | 1.3284 | 7.05354582** |
| miR4368 | AAGACGGGACTTACTATCAGTAA | 0 | 26 | 0.01 | 1.2792 | 6.99909803** |
| miR4408 | TAAGGACATTGGTGAAGGGTTGA | 0 | 25 | 0.01 | 1.23 | 6.94251451** |
| miR5486 | AAGGGATGCATATTCTACTCA | 0 | 25 | 0.01 | 1.23 | 6.94251451** |
| miR6188 | CGTGCGATGATGATCCCGGCGA | 0 | 25 | 0.01 | 1.23 | 6.94251451** |
| miR1450 | TTCAAATTGGATCGGATCAGGTTA | 0 | 24 | 0.01 | 1.1808 | 6.88362082** |
| miR2660 | TAAAGACATTCAGCATATAACTCA | 0 | 24 | 0.01 | 1.1808 | 6.88362082** |
| miR3440 | TGAGATTGAGGGTCAAGGGAAGGT | 0 | 24 | 0.01 | 1.1808 | 6.88362082** |
| miR4398 | TGTAGCGGAGAGAGAAAGAGGAA | 0 | 24 | 0.01 | 1.1808 | 6.88362082** |
| miR1122 | TAAGACACATCGTATTTGGAC | 0 | 23 | 0.01 | 1.1316 | 6.82222027** |
| miR1441 | ACGGATGTCGGATGAAATGGTTGT | 0 | 23 | 0.01 | 1.1316 | 6.82222027** |
| miR4382 | ATGATTAACTGAGTTGCATGGAGT | 0 | 23 | 0.01 | 1.1316 | 6.82222027** |
| miR7774 | ACTTTGGTATGTGAGGATGCAACA | 0 | 23 | 0.01 | 1.1316 | 6.82222027** |
| miR7995 | TGACAAGAGACGAAGTTGACATT | 0 | 23 | 0.01 | 1.1316 | 6.82222027** |
| miR5558 | TGAGACTTTAGAATTAGAAATGGC | 23 | 2417 | 1.0558 | 118.913 | 6.81542606** |
| miR5137 | AGAGGAGAAAACGATGGAGCT | 0 | 22 | 0.01 | 1.0824 | 6.75808993** |
| miR906 | CAGTTGGTGGGGTGATGAC | 0 | 22 | 0.01 | 1.0824 | 6.75808993** |
| miR1440 | TTTAGGAGAGATTGGCTATTAGAG | 9 | 870 | 0.4132 | 42.8028 | 6.69472111** |
| miR2667 | TACCTTGATTGACGGCTCTCC | 0 | 21 | 0.01 | 1.0332 | 6.69097574** |
| miR2093 | GGCATTAATGTGGAAGAAG | 1 | 96 | 0.0459 | 4.7231 | 6.68509612** |
| miR5149 | GAGAGGCTTGTGACGATTTGGG | 4 | 348 | 0.1836 | 17.1211 | 6.54306553** |
| miR5635 | TTTTAGGATTGTAACGGTG | 4 | 320 | 0.1836 | 15.7436 | 6.4220556** |
| miR2928 | AAGAAGAGGACATTGATG | 7 | 481 | 0.3213 | 23.6645 | 6.20265965** |
| miR8744 | TAAAGAGTGGGCAAAATGGT | 8 | 540 | 0.3672 | 26.5673 | 6.17694175** |
| miR4401 | ACAAGTATTTAAAGTAGCTGCATT | 3 | 199 | 0.1377 | 9.7905 | 6.15178208** |
| miR5633 | TTGAACATCAGAAATCAGTGC | 2 | 127 | 0.0918 | 6.2482 | 6.08880267** |
| miR1091 | CGGCATGTGAGGGAAGAGTTG | 6 | 364 | 0.2754 | 17.9083 | 6.02295602** |
| miR2919 | AATGGGAGGGGGGGGCAAGAA | 6 | 361 | 0.2754 | 17.7607 | 6.01101607** |
| miR5782 | TAGCGGAAGGAGAAGTCG | 280 | 15688 | 12.8537 | 771.828 | 5.9080234** |
| miR3520 | AGGTGATCGGTGAATAATTATCCT | 27 | 1236 | 1.2395 | 60.8095 | 5.61646655** |
| miR7770 | CCTTGAAACCTGTACCAGTAGACA | 2 | 90 | 0.0918 | 4.4279 | 5.59198468** |
| miR8048 | AGATGACATGCTACTGAATGA | 2 | 82 | 0.0918 | 4.0343 | 5.45768041** |
| miR7745 | AGTAAGGCATTTAGAAAGGAT | 15 | 609 | 0.6886 | 29.962 | 5.44332394** |
| miR2593 | TAAATGATTGAACTAGACAT | 4 | 162 | 0.1836 | 7.9702 | 5.43997796** |
| miR7499 | ATATGATTTTCGGTTTTTGGTAT | 5 | 180 | 0.2295 | 8.8558 | 5.27005658** |
| miR6271 | TCAAGGTTGTAGAGATAAAAT | 2 | 72 | 0.0918 | 3.5423 | 5.27004844** |
| miR5536 | AAGGTAGTGACTATGTACGGTAGT | 2 | 69 | 0.0918 | 3.3947 | 5.20864612** |
| miR5507 | AATGAGAATGGACCGGAG | 9 | 308 | 0.4132 | 15.1532 | 5.19663842** |
| miR7125 | CTAGTGGAATAAGTTCAAT | 1 | 34 | 0.0459 | 1.6728 | 5.187627** |
| miR824 | TAGAATTTTTGAGAAGGCA | 1 | 34 | 0.0459 | 1.6728 | 5.187627** |
| miR5830 | ATGAGAGGAGGTGATGTGACATCA | 7 | 226 | 0.3213 | 11.1189 | 5.11294928** |
| miR5014 | TTGTACAAATTTATTTTGTAC | 3 | 81 | 0.1377 | 3.9851 | 4.85501546** |
| miR6439 | CAAATAAAGAAGCAACGCC | 7 | 178 | 0.3213 | 8.7574 | 4.76850972** |
| miR3933 | AGAAGAAAATGAAGAACACGG | 6 | 152 | 0.2754 | 7.4782 | 4.76309059** |
| miR7121 | TCCTCTTGGGATCGACACTCGT | 13 | 325 | 0.5968 | 15.9895 | 4.74373348** |
| miR4250 | TCCAAAGCTGAAGACATCA | 7 | 171 | 0.3213 | 8.413 | 4.71062746** |
| miR6269 | TGTGAATAAGTGATTGTCTGA | 2 | 47 | 0.0918 | 2.3123 | 4.65469062** |
| miR395 | TAACTGAACTGCCTGTAGGGA | 21 | 479 | 0.964 | 23.5661 | 4.61153607** |
| miR6214 | TGACGACGACGACGACGACGACA | 18 | 410 | 0.8263 | 20.1714 | 4.60950174** |
| miR7707 | TTTGAGTCGAAGATGGCTGAATG | 37 | 830 | 1.6985 | 40.8348 | 4.58746604** |
| miR5262 | TCTGAATCAGTAGACTCAATC | 6 | 133 | 0.2754 | 6.5434 | 4.57044** |
| miR7484 | TTTGTAGAAGATCAAGAGTGCAA | 2 | 44 | 0.0918 | 2.1647 | 4.55952914** |
| miR5176 | TGTGATGATGTGGCATTGACCGAT | 34 | 737 | 1.5608 | 36.2594 | 4.53799746** |
| miR6162 | AAAAAAGATGTAGGCATTGGA | 4 | 81 | 0.1836 | 3.9851 | 4.43997796** |
| miR5210 | ATAAGTGCGTTTGGAATTAAGGTT | 18 | 352 | 0.8263 | 17.3179 | 4.38945452** |
| miR5340 | TGATGATGATGGTGAATTTGATAA | 2 | 38 | 0.0918 | 1.8695 | 4.34801451** |
| miR7724 | TGAACACTGGACTGCGGCCAACCA | 2 | 36 | 0.0918 | 1.7712 | 4.27008916** |
| miR7821 | AGATGGGCAAGGGCATTTGCA | 64 | 1047 | 2.938 | 51.5109 | 4.13197145** |
| miR3437 | AAAAATACAAGGACTAAACGGAT | 73 | 1070 | 3.3511 | 52.6425 | 3.97352136** |
| miR4366 | CATCACTTGTAGAGATTGTTGGA | 5 | 73 | 0.2295 | 3.5915 | 3.96802046** |
| miR5251 | ATAGATGTAGTTGGTCTTT | 3 | 40 | 0.1377 | 1.9679 | 3.83705645** |
| miR1869 | TGAGAATAATAGGCGATGGATA | 13 | 167 | 0.5968 | 8.2162 | 3.78315186** |
| miR1136 | TGTGTGCGGTGTGGTGTATCTA | 2 | 25 | 0.0918 | 1.23 | 3.74402035** |
| miR822 | TGCGAGGAGGCATTTGGACAG | 8 | 100 | 0.3672 | 4.9199 | 3.74399103** |
| miR6430 | TGATGATAATTGGACATTGCACAA | 8 | 94 | 0.3672 | 4.6247 | 3.65472182** |
| miR2643 | TTTGGAGATGAGAAATTATGGTAGA | 31 | 350 | 1.4231 | 17.2195 | 3.5969343** |
| miR3638 | GAACAAGCAGAAAGAGGACACC | 36 | 390 | 1.6526 | 19.1875 | 3.53735727** |
| miR1875 | AGAATGGAGTTGAAGCAACAGAC | 5 | 54 | 0.2295 | 2.6567 | 3.53306927** |
| miR6476 | TCATGTGGAGAATGAAATAATGA | 12 | 127 | 0.5509 | 6.2482 | 3.50357827** |
| miR2087 | GAGAGGAAAGAACCGGCTCA | 8 | 84 | 0.3672 | 4.1327 | 3.49244668** |
| miR6427 | GTGGAGAATGAAATTATGAAGA | 25 | 254 | 1.1477 | 12.4964 | 3.44469505** |
| miR859 | TGATATTTACAAATCAGACTAGTA | 9 | 87 | 0.4132 | 4.2803 | 3.37279976** |
| miR5542 | TTTGAGAAGGTGATGTGACAT | 77 | 735 | 3.5348 | 36.161 | 3.35473408** |
| miR3949 | TGATGTTGAGCAAAAGATTGATAA | 5 | 47 | 0.2295 | 2.3123 | 3.33276253** |
| miR5013 | TTTGTGACAGTCAGAGTGCCTT | 7 | 61 | 0.3213 | 3.0011 | 3.22349851** |
| miR5304 | AAGATGAGGATGATAGCATTGGA | 73 | 590 | 3.3511 | 29.0272 | 3.11469877** |
| miR414 | TCATCATCATCATCGTCGTCGTC | 30 | 227 | 1.3772 | 11.1681 | 3.01957377** |
| miR1919 | ACGTAGAGTCAATCGTGACGG | 9 | 66 | 0.4132 | 3.2471 | 2.97423966** |
| miR8044 | TTTCATATATGGTTGGAGGT | 4 | 26 | 0.1836 | 1.2792 | 2.80060388** |
| miR1103 | GAAAAAGACGGTGACATTCTTGT | 6 | 38 | 0.2754 | 1.8695 | 2.76305201** |
| miR7508 | CAAGAGAGAGTAGATCGGGAGAG | 34 | 211 | 1.5608 | 10.3809 | 2.73357394** |
| miR6170 | CAAGAAAAAGTAGAGATGGGCAT | 37 | 217 | 1.6985 | 10.6761 | 2.6520516** |
| miR863 | TTGAGAGGAAGCATAGACATATT | 14 | 79 | 0.6427 | 3.8867 | 2.59632838** |
| miR1511 | CCTAGCTCTGATACCATGAAGAA | 4 | 22 | 0.1836 | 1.0824 | 2.55959578** |
| miR1169 | TCTCCAGTCTAAAGCAACATCACA | 6 | 32 | 0.2754 | 1.5744 | 2.51520166** |
| miR5258 | TTAAGTGACAAGGAGGATTT | 4 | 21 | 0.1836 | 1.0332 | 2.49248158** |
| miR5256 | AAGGTTATGTAAGATTCAA | 6 | 30 | 0.2754 | 1.476 | 2.42209226** |
| miR8024 | TTTAAAATTTAAAGGACGTCAACT | 6 | 29 | 0.2754 | 1.4268 | 2.37318266** |
| miR3705 | GAGGTGGTTATGAGCGGAC | 35 | 169 | 1.6067 | 8.3146 | 2.37154628** |
| miR5081 | TATTTTGTAGAAATTGATATGGTA | 6 | 27 | 0.2754 | 1.3284 | 2.27008916** |
| miR7834 | AAAAAATCTCGGACATTAT | 20 | 90 | 0.9181 | 4.4279 | 2.26989943** |
| miR418 | TTTGTGATGATTGAAATGAGG | 52 | 218 | 2.3871 | 10.7253 | 2.16768709** |
| miR1100 | TCCACGGAAGAACCCCAACTC | 65 | 264 | 2.9839 | 12.9884 | 2.12195263** |
| miR4342 | TATGACTTGAATGTAGGGAATGGT | 7 | 28 | 0.3213 | 1.3776 | 2.10016416** |
| miR6261 | AAGTGTATCAATGGTAGAAGCAGC | 7 | 28 | 0.3213 | 1.3776 | 2.10016416** |
| miR8153 | TGCACTGTAGACTGTAGCGGAGC | 326 | 1259 | 14.9654 | 61.9411 | 2.04926616** |
| miR2119 | CAAAGGGAGGTGGTGGATTAA | 80 | 304 | 3.6725 | 14.9564 | 2.02592856** |
| miR7497 | ATTGTGGACTGTCATATATGGTTA | 64 | 229 | 2.938 | 11.2665 | 1.9391331** |
| miR5720 | TTGTGATTTGGGTTGGACAGC | 24 | 84 | 1.1017 | 4.1327 | 1.90735322** |
| miR6429 | TAGTAGAAGTGGAATTGATAG | 26 | 90 | 1.1936 | 4.4279 | 1.8913032** |
| miR7507 | AAGGTGGGAGTAGGCAATAATGGG | 10 | 32 | 0.4591 | 1.5744 | 1.77792179** |
| miR6257 | CTTAACTGTAATGGATTAGGGCAT | 20 | 63 | 0.9181 | 3.0995 | 1.7553123** |
| miR5179 | TCTTGCTCAAGACCGCGCAAT | 16 | 50 | 0.7345 | 2.4599 | 1.74376527** |
| miR946 | TGTGTGTAGAGAGAGAGGGTTAGT | 23 | 71 | 1.0558 | 3.4931 | 1.72617137** |
| miR7485 | AAAGACCATCTTTGATTCGTTTGA | 120 | 347 | 5.5087 | 17.0719 | 1.63183983** |
| miR5578 | CTATGTGGCATGAGGAGATTAGT | 38 | 106 | 1.7444 | 5.2151 | 1.57996402** |
| miR2610 | AAAGTTGTAGACTTGTATGGCAT | 17 | 47 | 0.7804 | 2.3123 | 1.5670429** |
| miR916 | AACCAAGGTCATCGGTTCGATACT | 38 | 102 | 1.7444 | 5.0183 | 1.52446782** |
| miR5246 | TTCAGACAGCTTTGATTGTTC | 12 | 32 | 0.5509 | 1.5744 | 1.51493976** |
| miR5818 | TCGAACTGAGAGGCACAGGTT | 362 | 960 | 16.618 | 47.2307 | 1.50697816** |
| miR8000 | ACACCGAAGAAGACAGAGAAGAA | 22 | 55 | 1.0099 | 2.7059 | 1.42189608** |
| miR7545 | TTGAAGAAATTAGAGTGCT | 138 | 326 | 6.3351 | 16.0387 | 1.34011791** |
| miR1139 | AGAGTAACACACATATATCA | 23 | 54 | 1.0558 | 2.6567 | 1.33129875** |
| miR6464 | ATAATTGATTGTTGGATATTT | 1157 | 2609 | 53.1134 | 128.359 | 1.27303891** |
| miR5274 | ATATGAGGAGTTTGTTGAAATGCC | 24 | 51 | 1.1017 | 2.5091 | 1.18743855** |
| miR7504 | AGGGAAAAATCTGATCTGTGCATT | 45 | 95 | 2.0658 | 4.6739 | 1.17792628** |
| miR8151 | TGGATACCAGTAGATCCCAT | 16 | 33 | 0.7345 | 1.6236 | 1.14436185** |
| miR5240 | TTAAAATATTGTGGTATTTA | 15 | 30 | 0.6886 | 1.476 | 1.09995464** |
| miR5561 | CATAAGAGAGAGAGCATAGACAA | 46 | 91 | 2.1117 | 4.4771 | 1.08415965** |
| miR7700 | TGAAGTTTGGGACAGCTTGCA | 58 | 113 | 2.6626 | 5.5594 | 1.06209348** |
| miR5303 | ACATATTATGAAGAGTTGAGCA | 12 | 23 | 0.5509 | 1.1316 | 1.03850171** |
| miR829 | AGCTCTGATACCAAATGATGCGA | 20 | 38 | 0.9181 | 1.8695 | 1.02592926** |
| **Down-regulated miRNAs** | |  |  |  |  |  |
| miR6231 | ATATTAGTGGAGTATGGACAT | 7994 | 0 | 366.974 | 0.01 | -15.1633895** |
| miR3710 | CGGGACCTGCACGGGCCACCA | 5218 | 0 | 239.538 | 0.01 | -14.547968** |
| miR1222 | CTGAAAGATCATTGGTGACA | 2094 | 0 | 96.1275 | 0.01 | -13.2307336** |
| miR6177 | TAGCATGGACAGAAGAGCATA | 1774 | 0 | 81.4375 | 0.01 | -12.991477** |
| miR780 | TTCTTCTGAAGAACTGGCAT | 1563 | 0 | 71.7513 | 0.01 | -12.8087892** |
| miR5629 | TTAGGATTTAACGGACGTTA | 1043 | 0 | 47.8801 | 0.01 | -12.2252107** |
| miR6151 | AGAGTGTGAGCAATTGGAGAG | 912 | 0 | 41.8664 | 0.01 | -12.0315773** |
| miR6219 | ATCAGGGACGAAAGTTGGG | 841 | 0 | 38.6071 | 0.01 | -11.9146506** |
| miR7714 | CTAAATATGTATGCACGGAGAGC | 619 | 0 | 28.4159 | 0.01 | -11.4724825** |
| miR6229 | ATATCTCACTTGAGCGTCGGAGG | 390 | 0 | 17.9034 | 0.01 | -10.8060179** |
| miR5794 | TGAGGAACACTAGTGGCAT | 365 | 0 | 16.7557 | 0.01 | -10.7104363** |
| miR6469 | CTGGCAAACAGGATCGTTTA | 336 | 0 | 15.4245 | 0.01 | -10.5910081** |
| miR2592 | ACAACAGCGACATCAAGAATATC | 1652 | 1 | 75.837 | 0.0492 | -10.590028** |
| miR1151 | ACTGGTTGTGGACACGGA | 304 | 0 | 13.9555 | 0.01 | -10.446618** |
| miR5829 | ATCAGGACTTAGGGATGGTAA | 290 | 0 | 13.3128 | 0.01 | -10.3785983** |
| miR5668 | AGAATCGGAATTATTGACAGC | 261 | 0 | 11.9815 | 0.01 | -10.2265929** |
| miR6425 | TTGCTTCCGTGGACATAGGCA | 256 | 0 | 11.752 | 0.01 | -10.1986906** |
| miR7693 | GACTCTCGCATCGATGAAGAACGTA | 4997 | 4 | 229.393 | 0.1968 | -10.186876** |
| miR6289 | TCCTTTGAAGGTGTTGGCTGA | 247 | 0 | 11.3388 | 0.01 | -10.1470523** |
| miR169 | AGGCAGTCTCCTTGGCTAAC | 228 | 0 | 10.4666 | 0.01 | -10.0315772** |
| miR5502 | CTACGGATCGGATACGGATTC | 221 | 0 | 10.1453 | 0.01 | -9.98659581** |
| miR5023 | TTTGGTAGTGATAAAGGCGGC | 214 | 0 | 9.8239 | 0.01 | -9.94015202** |
| miR1109 | TAGTGGGAGATTGTTGGGAA | 199 | 0 | 9.1353 | 0.01 | -9.8353083** |
| miR771 | GATGCCCTCAGATGTTCTG | 195 | 0 | 8.9517 | 0.01 | -9.80601794** |
| miR1025 | TCCACAACAGAAGCTGAATATACA | 193 | 0 | 8.8599 | 0.01 | -9.79114662** |
| miR5200 | TGTAGATTCTCTGTAGAGGGCTT | 189 | 0 | 8.6763 | 0.01 | -9.76093617** |
| miR1221 | TGATGGTGTGCAGTGGATATCAAA | 172 | 0 | 7.8959 | 0.01 | -9.62495988** |
| miR1536 | AAGCTGGAGAATGTGTTTA | 168 | 0 | 7.7122 | 0.01 | -9.59099862** |
| miR7767 | TCCCACAGTTGAGGCTCTCCC | 161 | 0 | 7.3909 | 0.01 | -9.52960624** |
| miR832 | TCTGGGAATCGGGAATCGAAAGAC | 149 | 0 | 6.84 | 0.01 | -9.41785252** |
| miR3438 | TCGATGCTTCATCTCGGACAC | 4764 | 7 | 218.697 | 0.3444 | -9.31063262** |
| miR6465 | TTAAAGCTGAAGGGGTTAGTAGAT | 129 | 0 | 5.9219 | 0.01 | -9.20991634** |
| miR5294 | GCTAGAATCGGAATGAGTTAGCAT | 125 | 0 | 5.7383 | 0.01 | -9.16447959** |
| miR5651 | TTGTGCGGATCAAATTGTTAGAC | 118 | 0 | 5.4169 | 0.01 | -9.08132367** |
| miR6199 | CGACAGAGACTTCTTAAGTGATGG | 103 | 0 | 4.7283 | 0.01 | -8.88517778** |
| miR5085 | AATTGAGACATTTTTTGTGGCATG | 101 | 0 | 4.6365 | 0.01 | -8.85689235** |
| miR1167 | GGGGATGTGATGAGTGGAAA | 98 | 0 | 4.4988 | 0.01 | -8.8133964** |
| miR5378 | CATCTGAAGAGATAGAGAGAATGA | 91 | 0 | 4.1775 | 0.01 | -8.70649601** |
| miR5053 | TCGGACTGTAGTCGCCGGCCT | 88 | 0 | 4.0397 | 0.01 | -8.65810437** |
| miR2644 | ACACTTAGATTGATGGGGT | 84 | 0 | 3.8561 | 0.01 | -8.59099868** |
| miR2620 | TTCTGATACGGCACCGCTCTC | 82 | 0 | 3.7643 | 0.01 | -8.5562378** |
| miR5247 | AATGAGCAAGCATCTGATA | 64 | 0 | 2.938 | 0.01 | -8.19869057** |
| miR5488 | AGAAGGCTACTATGATTTA | 64 | 0 | 2.938 | 0.01 | -8.19869057** |
| miR7708 | TGTCATGAACTGAACGAAAGACG | 311 | 1 | 14.2768 | 0.0492 | -8.18079862** |
| miR4239 | TTTGTTATTTTCGATTGTCTC | 52 | 0 | 2.3871 | 0.01 | -7.8991152** |
| miR1104 | CGCAGTTGTTCGTCTTTCCTT | 51 | 0 | 2.3412 | 0.01 | -7.87110436** |
| miR4230 | TGTCTCGGTGAATTTGCATCCCTA | 50 | 0 | 2.2953 | 0.01 | -7.84253891** |
| miR7546 | TTGGTACCGATCGGAAGCGCGTGC | 49 | 0 | 2.2494 | 0.01 | -7.81339644** |
| miR5508 | TGATGGCTGATTGGCTGTGGT | 46 | 0 | 2.1117 | 0.01 | -7.7222611** |
| miR5543 | TATGAGTGGCTAATTTTCTTT | 46 | 0 | 2.1117 | 0.01 | -7.7222611** |
| miR7720 | TTTAAAATATTTGGGTTGGATC | 44 | 0 | 2.0199 | 0.01 | -7.65814007** |
| miR5290 | AATGTGAGTAGAGTAGACACCTA | 5595 | 27 | 256.845 | 1.3284 | -7.59506455** |
| miR2102 | ATGGTGGTCCGGTGGCCGGTGGCG | 42 | 0 | 1.9281 | 0.01 | -7.59103606** |
| miR6167 | AACACCAGTGGAAGTTTTGA | 41 | 0 | 1.8822 | 0.01 | -7.55627612** |
| miR6421 | CAAAGCAGATTGTAAGGGG | 40 | 0 | 1.8362 | 0.01 | -7.52057941** |
| miR839 | GAACGCATGGAGGATGGAGCAA | 37 | 0 | 1.6985 | 0.01 | -7.40811742** |
| miR4992 | TTCTGTAGATGGTTTTTTTAG | 36 | 0 | 1.6526 | 0.01 | -7.36859377** |
| miR5630 | GCTAAGGGCGTTTTCTGATGGCA | 36 | 0 | 1.6526 | 0.01 | -7.36859377** |
| miR7544 | TTAGAACAGAAGATTTGTTAGCCG | 36 | 0 | 1.6526 | 0.01 | -7.36859377** |
| miR5791 | TTGCGGAGACTGGAGAGACGAG | 33 | 0 | 1.5149 | 0.01 | -7.24307875** |
| miR1037 | AGCCTTGTTGGATATTGATGG | 29 | 0 | 1.3313 | 0.01 | -7.05669189** |
| miR7787 | GTCTACGTCCACAAGAGCAAGAGA | 29 | 0 | 1.3313 | 0.01 | -7.05669189** |
| miR5292 | ATTCAGTAGAGCAACAAAGAAGGC | 142 | 1 | 6.5187 | 0.0492 | -7.04978216** |
| miR5506 | TGGTAGCTTCGTCGACGGT | 27 | 0 | 1.2395 | 0.01 | -6.95361445** |
| miR5725 | ATTTGGCATATCTGAATCTG | 26 | 0 | 1.1936 | 0.01 | -6.89917563** |
| miR5779 | CAATAAAAGTAGAGAAGTTGCA | 26 | 0 | 1.1936 | 0.01 | -6.89917563** |
| miR6212 | ATAAGATTTACAATGACGGT | 25 | 0 | 1.1477 | 0.01 | -6.84260178** |
| miR2665 | TCATTTCAGGAAGAATTGCA | 837 | 7 | 38.4235 | 0.3444 | -6.80175998** |
| miR5146 | TTTCAACTAATGAACCAAGCACAT | 24 | 0 | 1.1017 | 0.01 | -6.78358761** |
| miR6191 | TAAAGATTTGTCAAGATTTGAA | 24 | 0 | 1.1017 | 0.01 | -6.78358761** |
| miR8135 | AGGATTTTCAGGTTCATT | 24 | 0 | 1.1017 | 0.01 | -6.78358761** |
| miR439 | TTCGAACCGGGTTGGTGA | 23 | 0 | 1.0558 | 0.01 | -6.72219276** |
| miR6247 | TGGCTGAATGAACATAAGGCA | 655 | 6 | 30.0685 | 0.2952 | -6.67041637** |
| miR6234 | TTAGATCAAGTAGAGAACACACGT | 22 | 0 | 1.0099 | 0.01 | -6.65806863** |
| miR5271 | TGATAATTCTGGAAAATAACGGTG | 411 | 4 | 18.8674 | 0.1968 | -6.58302159** |
| miR841 | TACGAGCCACTTGAAGATGAACA | 601 | 6 | 27.5896 | 0.2952 | -6.54628801** |
| miR5148 | TAGAGGCCTAGAAATGTCATACT | 380 | 4 | 17.4443 | 0.1968 | -6.46988167** |
| miR5649 | ATTGCAATTGTTGGTTATTTT | 270 | 3 | 12.3947 | 0.1476 | -6.39188682** |
| miR3476 | AGACCAACAACAGCAGCTCTA | 83 | 1 | 3.8102 | 0.0492 | -6.2750646** |
| miR5261 | TGATTTAGATGGCTTTGT | 943 | 13 | 43.2895 | 0.6396 | -6.08070339** |
| miR5029 | AATGACGAGAGAAACTGCA | 546 | 8 | 25.0648 | 0.3936 | -5.99278869** |
| miR3637 | AATATGTTTGTGTTTTCGTCTGA | 1123 | 18 | 51.5526 | 0.8856 | -5.86324616** |
| miR5641 | TTGTAAGTAGATGATGAGAATTA | 5658 | 105 | 259.737 | 5.1659 | -5.65188858** |
| miR1052 | TTTCTCTTTTATTGTTTGGTA | 51 | 1 | 2.3412 | 0.0492 | -5.57244606** |
| miR394 | AGGTGGGGATGACGTCAAGT | 374 | 8 | 17.1689 | 0.3936 | -5.44692358** |
| miR5198 | GGGAGAAAGAGAGATTGTTGGGAG | 310 | 7 | 14.2309 | 0.3444 | -5.36879795** |
| miR5762 | TCATGAGGAATAGACTGGC | 4231 | 105 | 194.229 | 5.1659 | -5.2325951** |
| miR1044 | TTGTGGGCATATTTCTTTTA | 236 | 6 | 10.8339 | 0.2952 | -5.19771615** |
| miR6480 | TATGCTGAAACGACGGAACAT | 267 | 7 | 12.2569 | 0.3444 | -5.15336519** |
| miR4240 | ATCGGCTAGAGTACAAACCCG | 618 | 18 | 28.37 | 0.8856 | -5.00156712** |
| miR6200 | TTTGTCCAAGCTAGATCTAA | 66 | 2 | 3.0298 | 0.0984 | -4.94442044** |
| miR4223 | AGGAGAATTTGCATAAATAAATCA | 162 | 5 | 7.4368 | 0.246 | -4.91795176** |
| miR1866 | ATGAAATTCCTGTGAAAGATTCCG | 189 | 6 | 8.6763 | 0.2952 | -4.87731531** |
| miR2676 | CATGTTTGGATAAAGTCTG | 113 | 4 | 5.1874 | 0.1968 | -4.7202095** |
| miR6485 | AGAATGTAGAAGAGGTAA | 695 | 25 | 31.9048 | 1.23 | -4.69704327** |
| miR5834 | TACGGATGAGAAAATGGTGT | 1340 | 50 | 61.5143 | 2.4599 | -4.64425025** |
| miR5242 | TTGTAGAAAAAAAGATGTC | 187 | 7 | 8.5845 | 0.3444 | -4.63957706** |
| miR6136 | TCAGACGAAGGATTGTATGACGG | 24 | 1 | 1.1017 | 0.0492 | -4.4849293** |
| miR2086 | AGACAAGTGAATGAGCAACTGGAA | 150 | 7 | 6.8859 | 0.3444 | -4.32148818** |
| miR5231 | TTTGCAAGTTGAAGGCTCA | 146 | 7 | 6.7023 | 0.3444 | -4.28249922** |
| miR5522 | AAAATAACGGAATTGGAGGCAT | 196 | 10 | 8.9976 | 0.492 | -4.19281001** |
| miR2616 | AATTCGGTTTGGTTCGGTTCGGAT | 486 | 27 | 22.3104 | 1.3284 | -4.06995485** |
| miR6444 | TACGGAAAATTTGAGATTGATGGT | 35 | 2 | 1.6067 | 0.0984 | -4.02929845** |
| miR5066 | AAGTGGATTAGAGTGGAGGCCT | 189 | 12 | 8.6763 | 0.5904 | -3.87731531** |
| miR5201 | AGGGAGGGCAAAATGATCAAT | 47 | 3 | 2.1576 | 0.1476 | -3.8696628** |
| miR5286 | AAAACGGATGGCAAAGACAGGA | 266 | 18 | 12.211 | 0.8856 | -3.78538232** |
| miR6446 | TGTGGGTGCTTGATGATGGA | 483 | 33 | 22.1727 | 1.6236 | -3.77151631** |
| miR6190 | CGAGAAAAGGAAAAGACAG | 380 | 29 | 17.4443 | 1.4268 | -3.61190068** |
| miR5763 | TGAATATGACTGAAAGACGCGTTA | 112 | 9 | 5.1415 | 0.4428 | -3.53746219** |
| miR4413 | TAAGAGGATTGTAAGTTACGTG | 1988 | 164 | 91.2614 | 8.0686 | -3.49961451** |
| miR5257 | TACAAGAAGAACCTTTTTCTG | 46 | 4 | 2.1117 | 0.1968 | -3.42360277** |
| miR5205 | CTTATTTAGGGAACGGACGGGAT | 23 | 2 | 1.0558 | 0.0984 | -3.42353445** |
| miR8015 | ATTGGAATTGAAAAGACGACTT | 33 | 3 | 1.5149 | 0.1476 | -3.35945794** |
| miR5291 | GTGGATTGATGGATTGGATTGGAT | 634 | 61 | 29.1045 | 3.0011 | -3.27767894** |
| miR5646 | GTTGAGGAACGTTGGAGT | 142 | 14 | 6.5187 | 0.6888 | -3.24242723** |
| miR821 | AAGTAGCAGACAAAAAAGCTGAAT | 40 | 4 | 1.8362 | 0.1968 | -3.22192108** |
| miR8136 | TGAAAAGTGGAATTGAGGAAAATA | 39 | 4 | 1.7903 | 0.1968 | -3.18539923** |
| miR3436 | CAACAAGTTTTTGGCGTCGT | 178 | 19 | 8.1713 | 0.9348 | -3.12783598** |
| miR6205 | AGGAGTTTGGAGACGTTGTTACAT | 27 | 3 | 1.2395 | 0.1476 | -3.06999364** |
| miR2949 | GCAAATCCAGTCAAACCACTTA | 125 | 14 | 5.7383 | 0.6888 | -3.05846635** |
| miR3513 | TTATAAGTAGAAATAAGTAT | 205 | 24 | 9.4108 | 1.1808 | -2.99455274** |
| miR5260 | TTTGGTATTGTTGACGGGCATT | 24 | 3 | 1.1017 | 0.1476 | -2.8999668** |
| miR6203 | AGACGATATCAGATAGACCTGCAT | 51 | 7 | 2.3412 | 0.3444 | -2.76509114** |
| miR8030 | TTCGGGTTCGGTTCGGTTCGGGTT | 283 | 39 | 12.9914 | 1.9187 | -2.75935585** |
| miR7743 | AATTTACTGACGTAGTTTGACTGC | 28 | 4 | 1.2854 | 0.1968 | -2.70741525** |
| miR7784 | CAGAGACTTAGAGACAGAGACAG | 35 | 5 | 1.6067 | 0.246 | -2.70737036** |
| miR5789 | TGAGGGTTCGTTCGGTAT | 90 | 13 | 4.1316 | 0.6396 | -2.69145874** |
| miR8045 | CTTGATAGTTAAGGATGGTTT | 26 | 4 | 1.1936 | 0.1968 | -2.60051731** |
| miR6273 | AAGTAGCATTTTTTTTTTT | 105 | 17 | 4.8201 | 0.8364 | -2.52679811** |
| miR5773 | TCTTTAAAAGTTCAGTAGAT | 49 | 8 | 2.2494 | 0.3936 | -2.51473811** |
| miR6209 | TCAGATCAGAAGGTTGCG | 176 | 30 | 8.0795 | 1.476 | -2.45257329** |
| miR838 | TGAAGAATAAGAGGAGAGCAAAGC | 49 | 9 | 2.2494 | 0.4428 | -2.3448131** |
| miR1439 | ATTTAGGAACAGGAGTGAGATATT | 37 | 7 | 1.6985 | 0.3444 | -2.30210417** |
| miR6028 | AGGAGATTAAGGACATTAA | 269 | 51 | 12.3488 | 2.5091 | -2.29912898** |
| miR1847 | TGGACTTTGCAGGTTGGGCAC | 686 | 138 | 31.4916 | 6.7894 | -2.21361107** |
| miR6182 | TGAGTGTGCTAGTATGGATGGTAT | 132 | 28 | 6.0596 | 1.3776 | -2.13706552** |
| miR2867 | GCAGGAAGATGTGTGGATGGCA | 48 | 11 | 2.2035 | 0.5412 | -2.02556315** |
| miR860 | TGCATTAGATTGGACTATAAG | 39 | 9 | 1.7903 | 0.4428 | -2.01547423** |
| miR5268 | ATAGAGTGGAATGAGAATGTGTT | 202 | 49 | 9.273 | 2.4107 | -1.94358403** |
| miR2923 | AAGGCAAAAGATACTAAATAAAAA | 43 | 11 | 1.974 | 0.5412 | -1.86688825** |
| miR902 | AGAAGGATCATTGGCAACTATTAC | 34 | 9 | 1.5608 | 0.4428 | -1.81755856** |
| miR5057 | AATGTCAAATCAGTTTGTACA | 163 | 45 | 7.4827 | 2.2139 | -1.75696888** |
| miR8033 | TGCCAAAGCTGAGACATATGTAGT | 39 | 11 | 1.7903 | 0.5412 | -1.72596762** |
| miR6426 | GATGGAGACAGTAGGTGAAGA | 494 | 146 | 22.6776 | 7.183 | -1.65860955** |
| miR812 | AAAAGGATGATAAGTTGGACA | 454 | 135 | 20.8414 | 6.6418 | -1.64980601** |
| miR6171 | ATTGTGGACGGCTGAAGGTTT | 509 | 154 | 23.3662 | 7.5766 | -1.62480064** |
| miR2111 | TAATCTGCATCCTGAGGTTTG | 39 | 12 | 1.7903 | 0.5904 | -1.60043673** |
| miR5158 | TGAGCCATGTGGGATGAGAATGAA | 117 | 37 | 5.371 | 1.8203 | -1.56101448** |
| miR1033 | TGAGGGCGTGATGTGGCAT | 562 | 193 | 25.7993 | 9.4953 | -1.44204643** |
| miR5278 | GAAAATATGCATGCAGAAATGTGA | 116 | 40 | 5.3251 | 1.9679 | -1.43615171** |
| miR5074 | GCAAGGCCACCGTGCCGGCGACGC | 631 | 218 | 28.9668 | 10.7253 | -1.43338231** |
| miR6277 | TGTGTGTAGAAAGGCGAAT | 43 | 15 | 1.974 | 0.738 | -1.41942927** |
| miR6449 | CTATGATTCTGGAAATAAACGGTT | 610 | 218 | 28.0028 | 10.7253 | -1.38455309** |
| miR1069 | TGATAGAATCAAAGGTGTCGACTG | 39 | 14 | 1.7903 | 0.6888 | -1.37804431** |
| miR5025 | ACTGTTATATGTAGATGACAT | 44 | 16 | 2.0199 | 0.7872 | -1.35948174** |
| miR855 | AGACAAGAAGAAGGAAAAGGAGA | 41 | 15 | 1.8822 | 0.738 | -1.35072721** |
| miR5767 | GGAGGATTTGGAGGTGCA | 115 | 44 | 5.2792 | 2.1647 | -1.28615222** |
| miR5152 | AGTCCTGCTATACCCACCA | 137 | 54 | 6.2891 | 2.6567 | -1.24321825** |
| miR1313 | TGTCCACTGGATTGTTGTTCG | 159 | 63 | 7.2991 | 3.0995 | -1.23568308** |
| miR5284 | TAGGGACCTAAGTGGAGAATCCT | 75 | 30 | 3.443 | 1.476 | -1.22197346** |
| miR7984 | TCCGACTTTGTGAAATGACTT | 1087 | 440 | 49.9 | 21.6474 | -1.20484606** |
| miR1521 | TCTTAATGGGAAAATGTCTGA | 24 | 10 | 1.1017 | 0.492 | -1.1630012** |
| miR2937 | TAAGGCTGTTGAAGAGGTC | 24 | 10 | 1.1017 | 0.492 | -1.1630012** |
| miR7130 | GATGGAATGTGGAGGATGTGTGT | 152 | 65 | 6.9777 | 3.1979 | -1.12562675** |
| miR5253 | GAAGTGAAAATAGATTTGTTGGAT | 42 | 18 | 1.9281 | 0.8856 | -1.12245275** |
| miR5712 | AATTATTAATTAATTGAGTGGAGA | 310 | 133 | 14.2309 | 6.5434 | -1.12091453** |
| miR5239 | TGGGAGAATAAGAAGAATGAGGTG | 79 | 34 | 3.6266 | 1.6728 | -1.11635266** |
| miR5535 | TCTCGTGCTTGAAGTGCTGGAT | 30 | 13 | 1.3772 | 0.6396 | -1.10649624** |
| miR3623 | TAACAAACTTCATCCAAGCACA | 25 | 11 | 1.1477 | 0.5412 | -1.08451184** |
| miR8146 | AGAAGACGGTAATTATGAAGCAA | 42 | 19 | 1.9281 | 0.9348 | -1.04445024** |
| miR7696 | TTCAAATGAGAACTTTGAAG | 16572 | 7497 | 760.757 | 368.842 | -1.04443257** |
| miR5054 | GTTCCCCACAGACGGCGCCA | 15839 | 7207 | 727.108 | 354.574 | -1.03608069** |
| miR5501 | ACTTGTGGCTAGGGGTGA | 163 | 75 | 7.4827 | 3.6899 | -1.01997722** |
| **Equally expressed miRNAs** | |  |  |  |  |  |
| miR5061 | TCTTTTCTGTCTGCTTCGGT | 13 | 24 | 0.5968 | 1.1808 | 0.98444519 |
| miR473 | TGAGGCCGTTGGGGAGAGTGG | 1361 | 2478 | 62.4783 | 121.914 | 0.96443907 |
| miR5716 | TTGATAATTGAGAGAGTACTGAAA | 77 | 138 | 3.5348 | 6.7894 | 0.9416555 |
| miR1873 | TACATGGTATCAGAGCAGGGAACG | 13 | 23 | 0.5968 | 1.1316 | 0.92304464 |
| miR8125 | CAGGAAAGAGAGAAGAGTA | 197 | 347 | 9.0435 | 17.0719 | 0.9166705 |
| miR4995 | GCAGTGGCTTGGTTAAGG | 40 | 70 | 1.8362 | 3.4439 | 0.90732005 |
| miR7753 | TGACAAGAAACGAAGACATGG | 23 | 40 | 1.0558 | 1.9679 | 0.89832034 |
| miR398 | GGGGCGACATGAGATCACATG | 988 | 1707 | 45.3553 | 83.982 | 0.888809 |
| miR6477 | TAAACAGTAGAAGGAATTCAT | 21 | 36 | 0.964 | 1.7712 | 0.87762208 |
| miR6035 | TGAAGTAAAAATGCATGACGT | 26 | 44 | 1.1936 | 2.1647 | 0.85884766 |
| miR3694 | ATTAAGGGGTTGTGGGTGGCGGCT | 22 | 37 | 1.0099 | 1.8203 | 0.84996379 |
| miR5266 | CGGGGGACTGACTGGGAACG | 14789 | 24781 | 678.906 | 1219.19 | 0.84463976 |
| miR2607 | TGTGATATATGTGATAGGTGAT | 17 | 28 | 0.7804 | 1.3776 | 0.81987136 |
| miR5052 | CCTGTGGACGTAGGCATA | 1590 | 2546 | 72.9908 | 125.26 | 0.77913579 |
| miR8042 | ATTAGACGGAAGTGGATT | 1091 | 1740 | 50.0836 | 85.6056 | 0.77336691 |
| miR2590 | ATCTAAACGGATGATTATCGAGCC | 17 | 27 | 0.7804 | 1.3284 | 0.76740394 |
| miR8005 | TTTAGAGTTTTACTGTTTAGGTATT | 36 | 57 | 1.6526 | 2.8043 | 0.76290312 |
| miR396 | GCTCAAGAAAGCTGTGGGAGA | 8151 | 12653 | 374.181 | 622.51 | 0.73436032 |
| miR5211 | TCGCAGGGGAGATGGGACCGC | 10428 | 15729 | 478.71 | 773.845 | 0.69289419 |
| miR5208 | ACATGGATGTAGGTGGGTTTGTTA | 98 | 146 | 4.4988 | 7.183 | 0.67504628 |
| miR5748 | AAAAGAACATTGGAAGGCTGTAAG | 37 | 55 | 1.6985 | 2.7059 | 0.67184731 |
| miR5999 | CTTCACGATCATGACGGACAA | 1540 | 2284 | 70.6955 | 112.37 | 0.6685615 |
| miR7783 | AGAGCTCTGATACCATGTGGAAAA | 19 | 28 | 0.8722 | 1.3776 | 0.65942615 |
| miR6460 | CTGATATGTGGATCATCGA | 47 | 69 | 2.1576 | 3.3947 | 0.65385666 |
| miR6192 | TGGAGAGGGGAGGAGAGGGAACTG | 30 | 44 | 1.3772 | 2.1647 | 0.65242901 |
| miR2097 | TCTCTCTCTCTTCTGTCGGCTTTT | 59 | 86 | 2.7085 | 4.2311 | 0.64353869 |
| miR4352 | TAATAAGTAGACATTTAATGACGG | 20 | 29 | 0.9181 | 1.4268 | 0.63605991 |
| miR834 | TGAGTAGGATCAGTAGGGTGGTAA | 108 | 156 | 4.9579 | 7.675 | 0.63043758 |
| miR3442 | TTCAGAGTCGTGAAATGTTGT | 28 | 40 | 1.2854 | 1.9679 | 0.61443953 |
| miR408 | ACGGGGAACAGGCAGAGCATG | 12594 | 17902 | 578.142 | 880.754 | 0.60731392 |
| miR845 | CAAGTGGTATCAGAGCTAAGG | 1269 | 1802 | 58.2549 | 88.6559 | 0.60583724 |
| miR6021 | TTGGAGATGCTTGCTATTGAA | 49 | 69 | 2.2494 | 3.3947 | 0.59374385 |
| miR4993 | GGCGGCGGAGGAGGAGGAG | 280 | 394 | 12.8537 | 19.3843 | 0.59270493 |
| miR909 | TGTGGTCAAAGAGTGTGGTGG | 15 | 21 | 0.6886 | 1.0332 | 0.58538146 |
| miR7814 | AATTGATTTTTATAGCTTTGA | 207 | 286 | 9.5026 | 14.0708 | 0.56631015 |
| miR5758 | TAAATTGGAAGAAGTCATTGG | 42 | 58 | 1.9281 | 2.8535 | 0.56555268 |
| miR5039 | CCCTTTCTGAATCGTTTGCTG | 27 | 37 | 1.2395 | 1.8203 | 0.55441797 |
| miR5494 | TTATAGGAAGGTTAGAGGT | 60 | 82 | 2.7544 | 4.0343 | 0.55058028 |
| miR5741 | TAGTTGACTAAATTAATGGTT | 48 | 65 | 2.2035 | 3.1979 | 0.53732793 |
| miR815 | AAGGGTGATGAGGAGGAGTGGG | 184 | 247 | 8.4467 | 12.1521 | 0.52474593 |
| miR1858 | GTGATGAGGAGGAGTGGGGTC | 240 | 317 | 11.0175 | 15.596 | 0.50137916 |
| miR7511 | AGAAGTTTGTGCTGTTGCTAG | 119 | 157 | 5.4628 | 7.7242 | 0.49974491 |
| miR5026 | AGTGCAATAAGATCGTGACACAT | 19 | 25 | 0.8722 | 1.23 | 0.49592742 |
| miR161 | TTAATGACTTAAAGTGACTTA | 133 | 175 | 6.1055 | 8.6098 | 0.49587028 |
| miR5562 | GAAGAGGAGAAGGCTGCAC | 337 | 443 | 15.4704 | 21.795 | 0.4944867 |
| miR8051 | GATAGTATGGTAGAAAGATTCA | 35 | 46 | 1.6067 | 2.2631 | 0.49419976 |
| miR2275 | AGAATTGGCAAGGAACAAAAGTGA | 32 | 42 | 1.469 | 2.0663 | 0.49221533 |
| miR952 | AAAACAGAACATGGCATTGGT | 2687 | 3496 | 123.35 | 171.998 | 0.47963776 |
| miR7838 | AGCATGTGCTGGGAGGAGAGAGAG | 633 | 814 | 29.0586 | 40.0477 | 0.46275419 |
| miR5185 | TTTAAAATTGAATCGAGATGC | 263 | 336 | 12.0733 | 16.5307 | 0.45332776 |
| miR5386 | CGTCGGCTGTCGGCGGACTG | 2596 | 3298 | 119.172 | 162.257 | 0.44523057 |
| miR6114 | TGAAATCGGAATCAGAATCGTGAG | 41 | 52 | 1.8822 | 2.5583 | 0.44276552 |
| miR7540 | TGATGATGATAGTGATGGTGA | 23 | 29 | 1.0558 | 1.4268 | 0.43444655 |
| miR1877 | AGATGACAGTGGATATGGAGGGGG | 100 | 126 | 4.5906 | 6.199 | 0.43335278 |
| miR5337 | TCTGGAACTGCAAGAATTTGA | 54 | 68 | 2.4789 | 3.3455 | 0.43252177 |
| miR172 | AGAATCTTGATGATGCTGCAT | 61936 | 77687 | 2843.24 | 3822.09 | 0.42682478 |
| miR3446 | CTCGGAGGCGTAGACGTAGGCAGG | 1865 | 2339 | 85.615 | 115.076 | 0.42664648 |
| miR157 | TTGACGGAAGATAGAGAGCAC | 1E+05 | 172840 | 6345.88 | 8503.49 | 0.42223354 |
| miR4411 | TCATGTAACTATATTATGTCGGAT | 20 | 25 | 0.9181 | 1.23 | 0.42193511 |
| miR535 | TGACAATGAGAGAGAGCACAC | 20658 | 25777 | 948.33 | 1268.19 | 0.41931354 |
| miR479 | TGTGATATTGGTTCGGCTCATC | 61386 | 76396 | 2818 | 3758.58 | 0.41551719 |
| miR4351 | ATTGGGAGTGTCGAGTGGGAGTGG | 9304 | 11535 | 427.111 | 567.506 | 0.41002493 |
| miR948 | TGTGGTCGTGGGTTCGGG | 112 | 138 | 5.1415 | 6.7894 | 0.40109477 |
| miR1436 | ACATAATGAGACGGAGAGGAGAT | 367 | 451 | 16.8476 | 22.1886 | 0.39727555 |
| miR5077 | TTCACGTCGGGTTCACCA | 3945 | 4843 | 181.1 | 238.269 | 0.3958057 |
| miR4387 | AAGAAGAGTGATGAGCGGATACT | 22 | 27 | 1.0099 | 1.3284 | 0.39547718 |
| miR7991 | AGGAGGTGGATTTTTAATTATATA | 127 | 155 | 5.8301 | 7.6258 | 0.38736807 |
| miR6455 | TAAAGATAGCATTCTCAACAT | 85 | 103 | 3.902 | 5.0675 | 0.37706041 |
| miR5373 | TGTCTTGATTTTAGATGCATG | 1404 | 1700 | 64.4523 | 83.6376 | 0.37591982 |
| miR6146 | TTTTGGCACAATAAATACTTAATC | 176 | 212 | 8.0795 | 10.4301 | 0.36841507 |
| miR5565 | TTTTGTTGGAAGATTGTCGGA | 249 | 299 | 11.4306 | 14.7104 | 0.36393534 |
| miR391 | ATCATGCTATCCCTTTGGATT | 105 | 126 | 4.8201 | 6.199 | 0.36297243 |
| miR319 | AGCTGCCGACTCATTCATTCA | 493 | 590 | 22.6317 | 29.0272 | 0.35906046 |
| miR5147 | ACAACTGTTGTGGATTTGAGGGT | 61 | 73 | 2.8003 | 3.5915 | 0.35900512 |
| miR1134 | CAGAACAAAGAAGAAGAAGAAGAT | 384 | 458 | 17.628 | 22.533 | 0.3541706 |
| miR390 | TTGGCAGGGAGATAGTGACCA | 16043 | 19034 | 736.473 | 936.446 | 0.34656457 |
| miR5221 | TGAGATGAGATGGTGGTTTTGACT | 22 | 26 | 1.0099 | 1.2792 | 0.3410294 |
| miR5272 | GAAATTGTTATGTTTGGATAA | 72 | 85 | 3.3052 | 4.1819 | 0.33942099 |
| miR5049 | AATTGGATATGGAGATCGGAGGGA | 90 | 106 | 4.1316 | 5.2151 | 0.33599433 |
| miR5544 | ATAACTGCGGAGTAGAAGTTGG | 303 | 355 | 13.9096 | 17.4655 | 0.32842701 |
| miR4414 | TGTGAATGATGCGGGAGATAA | 789 | 923 | 36.22 | 45.4103 | 0.32623302 |
| miR5140 | AGCTGGTTAAAGATTTGGATG | 24 | 28 | 1.1017 | 1.3776 | 0.32242563 |
| miR482 | AATGGGAGGCTTGGCAAGAAG | 7917 | 9205 | 363.439 | 452.873 | 0.31739353 |
| miR5788 | TGGATGTAGAGCATACTCAGTATA | 50 | 58 | 2.2953 | 2.8535 | 0.31404983 |
| miR5541 | TCAAAGTGGTGTATGTAATGA | 25 | 29 | 1.1477 | 1.4268 | 0.31403754 |
| miR5163 | GTAGAATAGTTCAGGTGTGTGGGA | 28 | 32 | 1.2854 | 1.5744 | 0.29258475 |
| miR6484 | TGATGGGCTCTGCAAGAATGG | 440 | 491 | 20.1987 | 24.1565 | 0.258149 |
| miR8154 | CAGAGAGGAAGGATGAAGAGGGGC | 130 | 145 | 5.9678 | 7.1338 | 0.25747158 |
| miR1507 | TCTTCATTCCACACGTCGTCT | 149 | 165 | 6.84 | 8.1178 | 0.24709247 |
| miR5523 | TGGGAGGGAATATTTTACTAG | 28 | 31 | 1.2854 | 1.5252 | 0.24678106 |
| miR5667 | AAAAGAACTCAAATGGATTGC | 66 | 73 | 3.0298 | 3.5915 | 0.24536395 |
| miR5281 | CTCTTGTAATAGGTCTGACGGGAA | 19 | 21 | 0.8722 | 1.0332 | 0.24438865 |
| miR7748 | AATTTCTTCTGATTGTTGGACTGG | 48 | 53 | 2.2035 | 2.6075 | 0.24287036 |
| miR5539 | AAGAAAACGGGATGGCGAGCT | 1464 | 1616 | 67.2066 | 79.505 | 0.24244267 |
| miR3948 | GGAGTGGGAGTGGGAGTAGGTTGT | 13095 | 14421 | 601.141 | 709.493 | 0.23908496 |
| miR2652 | TTATGCAGGGTGGATTGGATT | 82 | 90 | 3.7643 | 4.4279 | 0.23424103 |
| miR1886 | TGAGAGAAGTGAGAGAAGTGT | 843 | 923 | 38.6989 | 45.4103 | 0.23072701 |
| miR6118 | TGGACGATATGGGTGGTTCGGAAA | 298 | 324 | 13.68 | 15.9404 | 0.2206196 |
| miR4375 | AGCACTATGGTCGGGCCTGGCAT | 23 | 25 | 1.0558 | 1.23 | 0.22032174 |
| miR8122 | TAAGGAAGGTTTTGTGGACAAG | 939 | 1019 | 43.1059 | 50.1334 | 0.21788673 |
| miR538 | TTCGCATGTGAATTATCGTCTGGA | 24 | 26 | 1.1017 | 1.2792 | 0.21551042 |
| miR6475 | TCTTGGAAGTAGAACGACG | 5844 | 6306 | 268.276 | 310.246 | 0.20969862 |
| miR1510 | AGTGGATAGGTGTAAAACAATACA | 241 | 260 | 11.0634 | 12.7916 | 0.20940191 |
| miR1223 | TTCTAAGTCATACAGCTTCT | 78 | 84 | 3.5807 | 4.1327 | 0.20684299 |
| miR3711 | GGCGCTAGAAGGAGGGCATG | 26 | 28 | 1.1936 | 1.3776 | 0.20683761 |
| miR1047 | TGATCCGAGATGACCAGTTGC | 105 | 113 | 4.8201 | 5.5594 | 0.20586611 |
| miR4249 | TAATTATGAGAAGTATGAGCTA | 465 | 500 | 21.3464 | 24.5993 | 0.20462448 |
| miR3950 | TATTTTCTGCAACATGATTGT | 802 | 861 | 36.8167 | 42.36 | 0.20234227 |
| miR5772 | TAGGAATGTGAGTAGAGTAAGCAT | 342 | 367 | 15.6999 | 18.0559 | 0.20171496 |
| miR5234 | TTTTGTTATGGATGGCTGAAG | 731 | 781 | 33.5574 | 38.4241 | 0.19538053 |
| miR173 | GATTCTCTGGGCGAAGTGGAATGC | 88 | 94 | 4.0397 | 4.6247 | 0.19511162 |
| miR1850 | TGGAAAGTAGAAGAGATTGGG | 1042 | 1113 | 47.8342 | 54.7581 | 0.19502992 |
| miR533 | GAGTGGCCAGGGCTGTTGGAGGGC | 112 | 119 | 5.1415 | 5.8546 | 0.18738129 |
| miR5161 | TTTGATAGAGTGGAGTATA | 3171 | 3357 | 145.568 | 165.16 | 0.18216449 |
| miR5293 | AGAGAACGAAGAGGAAGGAAGAAG | 138 | 146 | 6.3351 | 7.183 | 0.18121912 |
| miR1310 | GAGGCATCGGGGGCGCAACGC | 629 | 665 | 28.875 | 32.7171 | 0.18022393 |
| miR7535 | TGGAAAATGACTGGGGGTGGT | 124 | 131 | 5.6924 | 6.445 | 0.17914332 |
| miR159 | TTTGGATTGAAGGGAGCTCTA | 2108 | 2224 | 96.7702 | 109.418 | 0.17721139 |
| miR1518 | TGTGCTGTAAAGGGAATAGTCCA | 91 | 96 | 4.1775 | 4.7231 | 0.17709425 |
| miR6029 | TCGCGGTTGTGGTTTAGGCTT | 94 | 99 | 4.3152 | 4.8707 | 0.1747017 |
| miR1171 | AGTGTGGAGTGGGAGTGGGAGTGG | 8972 | 9438 | 411.87 | 464.337 | 0.17298129 |
| miR3951 | TAGATAAAGATGAGAGAAAAA | 38252 | 40126 | 1756 | 1974.14 | 0.16893196 |
| miR8003 | TTTCTGGTAACAAATGGGAGTC | 205 | 215 | 9.4108 | 10.5777 | 0.16863669 |
| miR6275 | AGTGGAAGTGCAAGTGGAAGCCAA | 21 | 22 | 0.964 | 1.0824 | 0.16712869 |
| miR165 | TCGGACCAGGCTTCATCCCCC | 1406 | 1471 | 64.5441 | 72.3712 | 0.16513047 |
| miR6281 | AGCTGAGATCGAGAGAGTGAG | 65 | 68 | 2.9839 | 3.3455 | 0.16502266 |
| miR8019 | AGGGAAGTAGGCATTTCTTTATTT | 87 | 91 | 3.9938 | 4.4771 | 0.16480245 |
| miR6233 | CAAGTTGTTTTGGAATTACTGG | 1288 | 1345 | 59.1271 | 66.1721 | 0.16240355 |
| miR4365 | AAGAACTTGTTCCCGTAGATGA | 24 | 25 | 1.1017 | 1.23 | 0.15892689 |
| miR857 | GTGTTGAATGTTGAATGGTTGGCA | 24 | 25 | 1.1017 | 1.23 | 0.15892689 |
| miR5070 | AACTAGTAGGTCAGAGACGT | 511 | 532 | 23.458 | 26.1737 | 0.15803787 |
| miR1048 | AAGAAATGAGTGTAGACGGAT | 25 | 26 | 1.1477 | 1.2792 | 0.15649626 |
| miR5653 | CTGGGTTGGGTAGAGTTGGTTGC | 25 | 26 | 1.1477 | 1.2792 | 0.15649626 |
| miR7129 | AGAAATCTAGAGATCGTGTAT | 2065 | 2144 | 94.7962 | 105.482 | 0.15409296 |
| miR5826 | TAGGGAAAGTAGAAGAGTGGAGT | 135 | 140 | 6.1973 | 6.8878 | 0.15240344 |
| miR5237 | TTAAAAGATTGTAAGTGTTGGGAT | 216 | 224 | 9.9157 | 11.0205 | 0.15240315 |
| miR6230 | CGACAAGTTTGAGGGATCTGA | 28 | 29 | 1.2854 | 1.4268 | 0.15056574 |
| miR166 | TCGGACCAGGCTTCATTCCCC | 8E+05 | 810265 | 35917.4 | 39863.9 | 0.15040063 |
| miR8143 | GATGAGATGGGGACAAGATAGGTG | 29 | 30 | 1.3313 | 1.476 | 0.14885701 |
| miR5259 | CAAGGGGTATTTGGATGGACA | 1110 | 1146 | 50.9558 | 56.3816 | 0.14597805 |
| miR854 | AATGAAGTAGAGGAGGAGGAG | 125 | 129 | 5.7383 | 6.3466 | 0.14536052 |
| miR6267 | TAGGAATAGGTCAGGCAATGT | 764 | 787 | 35.0723 | 38.7193 | 0.14272083 |
| miR7764 | CAAAACCTTAGATCTGGATCAA | 274 | 281 | 12.5783 | 13.8248 | 0.13632166 |
| miR2600 | AATTCATTGTGATTGTGATGGT | 46 | 47 | 2.1117 | 2.3123 | 0.13092369 |
| miR5770 | TTCAGGATATGGTTTTGATAA | 30478 | 30672 | 1399.13 | 1509.02 | 0.10908378 |
| miR5296 | ATTTTGTGGGTTGTAGACAGGTT | 36 | 36 | 1.6526 | 1.7712 | 0.09998955 |
| miR5793 | AGGGGCAGATACAGTGCAG | 68 | 68 | 3.1216 | 3.3455 | 0.09993616 |
| miR5747 | AAAACGAAGACTCATACAATCATT | 27 | 27 | 1.2395 | 1.3284 | 0.09993136 |
| miR6196 | AGGAGAGGTATAGATGGACGAGGA | 141 | 141 | 6.4728 | 6.937 | 0.09992196 |
| miR5490 | ATTGAATTATTTATTTAGGACGTG | 25 | 25 | 1.1477 | 1.23 | 0.09991273 |
| miR774 | TGAGATGGAAGATGATGGTAT | 898 | 895 | 41.2237 | 44.0328 | 0.09510459 |
| miR2670 | TCTCAACATGGACGTGATCAA | 189 | 188 | 8.6763 | 9.2493 | 0.09226425 |
| miR5079 | TTTGGATATGTTATGTTTGGTGGT | 118 | 117 | 5.4169 | 5.7562 | 0.08764926 |
| miR5254 | AGCGGTGGAAGCAATTGTGTA | 4366 | 4327 | 200.426 | 212.882 | 0.08698485 |
| miR8155 | TAACCTGGCTCTGATACCA | 368 | 362 | 16.8935 | 17.8099 | 0.07621116 |
| miR5244 | TTATCGGATGAAGATTGTTGG | 1047 | 1029 | 48.0638 | 50.6254 | 0.07491069 |
| miR7520 | GAAGGGGAAGGTGAGACAC | 58 | 57 | 2.6626 | 2.8043 | 0.07480499 |
| miR5813 | ACAGCAGGACGGTGGTCATGGA | 3916 | 3848 | 179.769 | 189.316 | 0.07465839 |
| miR5241 | TGCCTGAATGGAAGAGTGCCT | 53 | 52 | 2.433 | 2.5583 | 0.07244913 |
| miR5076 | GAATGGAGAGGATGATGCAGGTTT | 52 | 51 | 2.3871 | 2.5091 | 0.07191097 |
| miR5169 | TTTGACCAAAGTTTGAGAGACATT | 52 | 51 | 2.3871 | 2.5091 | 0.07191097 |
| miR5534 | CTTTAGACAACAGTAGAATGG | 1511 | 1480 | 69.3642 | 72.8139 | 0.07002263 |
| miR4364 | CGTAGATCGGCAGCGGAAGAAGTT | 271 | 265 | 12.4406 | 13.0376 | 0.06762225 |
| miR168 | TCGCTTGGTGCAGGTCGGGAA | 91032 | 88888 | 4178.93 | 4373.17 | 0.06554469 |
| miR1863 | AGAGTTTGTGGCTGTATCATTACT | 2171 | 2116 | 99.6623 | 104.104 | 0.06290989 |
| miR2621 | AGCAATTGGGCTAGCGAATTGGGC | 1003 | 975 | 46.0439 | 47.9686 | 0.0590803 |
| miR3444 | TTGGAGCTGATGAGATGGT | 100 | 97 | 4.5906 | 4.7723 | 0.05600201 |
| miR8126 | TCTGACTCCCAGATTACTGACATA | 228 | 221 | 10.4666 | 10.8729 | 0.05494392 |
| miR1446 | CGAACTCTCTCCCTCAACGGC | 40402 | 39118 | 1854.7 | 1924.55 | 0.05333566 |
| miR5298 | TGAGGAAATGAATATGAAGACAA | 497 | 481 | 22.8154 | 23.6645 | 0.05271649 |
| miR861 | CTTGGAGAAATTATGAGCGTCAGA | 1521 | 1468 | 69.8233 | 72.2236 | 0.04876179 |
| miR6472 | TAGGTTAATTCGTAGATCTCAATC | 104 | 100 | 4.7742 | 4.9199 | 0.04336999 |
| miR4406 | ACATTGTACTAGAGAACCGGTGTA | 668 | 642 | 30.6653 | 31.5855 | 0.04265535 |
| miR1861 | TGATCTTGAGGCAAGAAGCTGT | 245 | 235 | 11.247 | 11.5617 | 0.03981331 |
| miR5269 | AGAAGATGGTGGGACAACTTGCTT | 416 | 398 | 19.097 | 19.581 | 0.03610843 |
| miR5666 | AGGGACATAGAGACATTTACT | 2944 | 2815 | 135.148 | 138.494 | 0.03528756 |
| miR5368 | GGGACAGTCTCAGGTAGACAGTA | 22 | 21 | 1.0099 | 1.0332 | 0.0329071 |
| miR6300 | GTCGTTGTAGTATAGTGGT | 12723 | 12142 | 584.064 | 597.37 | 0.03249687 |
| miR2873 | ACAATTTGGACTTAATTTGGAACA | 41 | 39 | 1.8822 | 1.9187 | 0.02770922 |
| miR5631 | TGACGAGGAAGAGATAATTTT | 187 | 177 | 8.5845 | 8.7082 | 0.02064043 |
| miR4346 | TAAACCCAACGAGAGAGCTGCAT | 112 | 106 | 5.1415 | 5.2151 | 0.0205056 |
| miR5492 | AGACTAGGAGAAACAGATATGGTT | 712 | 672 | 32.6852 | 33.0615 | 0.01651466 |
| miR5675 | TCAGAGACGGACAACTGGAAA | 35 | 33 | 1.6067 | 1.6236 | 0.01509567 |
| miR3630 | GCAACTGATGATAGAACAGACA | 66 | 62 | 3.0298 | 3.0503 | 0.00972858 |
| miR7825 | TTGGAAGAAATGGTAGAAGAGGAG | 181 | 170 | 8.309 | 8.3638 | 0.00948371 |
| miR1520 | TGTCACGATCCTGTTGGACTAA | 292 | 274 | 13.4046 | 13.4804 | 0.00813514 |
| miR530 | TGCATTTGCACCTGCACCTTG | 32 | 30 | 1.469 | 1.476 | 0.00685833 |
| miR7772 | AGACTAATGAATGAGAAACGGT | 80 | 75 | 3.6725 | 3.6899 | 0.00681923 |
| miR7812 | TGTTAGTGAATTGATGGGTGA | 700 | 655 | 32.1343 | 32.2251 | 0.00407079 |
| miR5632 | TTTATTGATAGTTGGATAAGT | 212 | 198 | 9.7321 | 9.7413 | 0.00136317 |
| miR1887 | CACTAAGTAGATCTAAGAGGAT | 22 | 10 | 1.0099 | 0.492 | -1.03748222 |
| miR3953 | TTGAGTTCTGCAAGCCGTCGA | 22 | 10 | 1.0099 | 0.492 | -1.03748222 |
| miR7532 | GAACAGCCTCTGGTCGATGGA | 4774 | 2290 | 219.156 | 112.665 | -0.95992133 |
| miR4374 | AAGACTAGAGACGTGATGTCAGCA | 32 | 16 | 1.469 | 0.7872 | -0.90003227 |
| miR4378 | CATAGGACTACTTAGAATGGCGA | 38 | 19 | 1.7444 | 0.9348 | -0.90000126 |
| miR2938 | GATCTTCTGAGAAGGGTTCGAG | 371 | 187 | 17.0312 | 9.2001 | -0.88845864 |
| miR5521 | AGTGGCATGCTTATCTGATGA | 29 | 15 | 1.3313 | 0.738 | -0.85114299 |
| miR7822 | TTTGAAATTGTAGCACAACGGTA | 23 | 12 | 1.0558 | 0.5904 | -0.83857194 |
| miR2666 | CAAAGTGTTGATAATCAAGGA | 36 | 19 | 1.6526 | 0.9348 | -0.82200793 |
| miR5020 | ATGGCATGCAAGAGGTCAG | 36 | 19 | 1.6526 | 0.9348 | -0.82200793 |
| miR5377 | CTGCAGTTGATCGATTTAGAATGT | 34 | 18 | 1.5608 | 0.8856 | -0.81755856 |
| miR6202 | GAAGGATTTAAAGCATTGA | 50 | 27 | 2.2953 | 1.3284 | -0.7889931 |
| miR5017 | ATATTCACACAAATTAATAGCAAA | 37 | 20 | 1.6985 | 0.984 | -0.787531 |
| miR3627 | TCTGTCGCAGGAGAGATGGTGCCTA | 2138 | 1160 | 98.1474 | 57.0704 | -0.78220738 |
| miR5225 | TCTGTCGCAGGAGAGATGGTGC | 2212 | 1213 | 101.544 | 59.6779 | -0.76684201 |
| miR4379 | AGACTGTATACTAGGGAAGGCCT | 218 | 120 | 10.0075 | 5.9038 | -0.76136586 |
| miR5810 | AGAACCCTATTGACGATGAGCAT | 29 | 16 | 1.3313 | 0.7872 | -0.75803358 |
| miR6117 | GGGTTGGGTTGATTGGGTTAGAC | 29 | 16 | 1.3313 | 0.7872 | -0.75803358 |
| miR5207 | ACTAATGTGGTTTCTGAGACGGTT | 27 | 15 | 1.2395 | 0.738 | -0.74806555 |
| miR7786 | ATGCACAAACTGTGGAATGGCGGC | 27 | 15 | 1.2395 | 0.738 | -0.74806555 |
| miR1160 | TGACAAGGAAGAGAGAGAGGA | 145 | 81 | 6.6564 | 3.9851 | -0.7401262 |
| miR2636 | TTTGTGTTGAAGATGGCTGAATAT | 1661 | 933 | 76.2501 | 45.9023 | -0.73217279 |
| miR2936 | GCTAGAGAGAGAGAAGCACGAGAG | 367 | 209 | 16.8476 | 10.2825 | -0.71235202 |
| miR167 | TGAAGCTGCCAGCATGATCTGA | 3E+05 | 184742 | 14829.4 | 9089.05 | -0.70625775 |
| miR7503 | AGATGGTGGCTGAAGCGAAGTTAG | 38 | 22 | 1.7444 | 1.0824 | -0.68849715 |
| miR2916 | GGGGCTCGAAGACGATCAGATA | 5378 | 3194 | 246.883 | 157.14 | -0.65177511 |
| miR1144 | TGGGTTTATGTGCGGCAGGCAG | 227 | 135 | 10.4207 | 6.6418 | -0.64980601 |
| miR1314 | TAGGCCATAGAATGTTAGGAGA | 104 | 62 | 4.7742 | 3.0503 | -0.64630786 |
| miR5072 | TTCGTTCCCCAGTGGAGTCGCCA | 188 | 113 | 8.6304 | 5.5594 | -0.63449824 |
| miR7758 | ATTGACCGTTAGTTGACCGTGTAA | 1228 | 740 | 56.3728 | 36.407 | -0.63078336 |
| miR6288 | AACCGAATTAGAAAAAACCGTCGG | 142 | 86 | 6.5187 | 4.2311 | -0.6235515 |
| miR8145 | TATAAAACAGTAGAATGACACATT | 23 | 14 | 1.0558 | 0.6888 | -0.61617952 |
| miR5628 | GAGGAATACGAAGAATATGATTAC | 67 | 41 | 3.0757 | 2.0171 | -0.60863218 |
| miR4391 | TCTATGGCAGAACTAAGAAGAAGA | 620 | 384 | 28.4618 | 18.8923 | -0.59122855 |
| miR8034 | ATATGACAGAAGATCTTCAAAAACT | 798 | 496 | 36.6331 | 24.4025 | -0.58611883 |
| miR2934 | CATCCAACGGTGCATTTGCTAGAA | 152 | 96 | 6.9777 | 4.7231 | -0.56301749 |
| miR4385 | AGATCATGTAGAAAGTAGAGTGGT | 38 | 24 | 1.7444 | 1.1808 | -0.56296627 |
| miR472 | TTTTTCCCACACCTCCCATCCC | 672 | 436 | 30.8489 | 21.4506 | -0.52420104 |
| miR5822 | TGTCTGCGAGTCGGGTTG | 1887 | 1225 | 86.6249 | 60.2683 | -0.52338241 |
| miR3633 | TTACCTATGCCACCCATTCCTT | 6158 | 4034 | 282.69 | 198.467 | -0.51032076 |
| miR5780 | AAACTTAACTGACGGTAGGGA | 3267 | 2144 | 149.975 | 105.482 | -0.50773178 |
| miR6157 | TTAGGTAATGTAGGATTTGCAAGA | 67 | 44 | 3.0757 | 2.1647 | -0.50674769 |
| miR5673 | CTTGGAATCTCGGGAAGCATG | 47 | 31 | 2.1576 | 1.5252 | -0.50042899 |
| miR6285 | TGTGAAAGTGTGAAATTAGGGCAT | 27 | 18 | 1.2395 | 0.8856 | -0.48503114 |
| miR5500 | ATTACTTGAAGAAAATCTTGCGGC | 114 | 76 | 5.2333 | 3.7391 | -0.48502991 |
| miR5059 | TCGTTCCTGGGCAGCAACACCA | 7995 | 5384 | 367.02 | 264.885 | -0.47049011 |
| miR5040 | ATTATACATATAAGAAGCATGAG | 57 | 39 | 2.6167 | 1.9187 | -0.44761938 |
| miR5083 | AGACTACAATTATCTGATCA | 45 | 31 | 2.0658 | 1.5252 | -0.43770215 |
| miR6282 | GTTGATCAAGTGTGGGCATTTACA | 29 | 20 | 1.3313 | 0.984 | -0.43610549 |
| miR5776 | AACTAGTGGCTGAGATTTAGGTG | 42 | 29 | 1.9281 | 1.4268 | -0.43439676 |
| miR7732 | GTAGAGATGTGGAGGAACAC | 255 | 178 | 11.7061 | 8.7574 | -0.41868599 |
| miR169 | CAGCCAAGGATGACTTGCCGG | 802 | 561 | 36.8167 | 27.6004 | -0.41567114 |
| miR3706 | CTTTCGGAGAAAAGGTAGGA | 87 | 61 | 3.9938 | 3.0011 | -0.4122707 |
| miR5380 | GGAAATGGAGTGAGGATGGGGAA | 134 | 94 | 6.1514 | 4.6247 | -0.41155501 |
| miR6280 | TTAGCATGTAAGATTCTTGGT | 222 | 157 | 10.1912 | 7.7242 | -0.39986651 |
| miR5745 | TGACAACACACTTTGGATGCA | 63 | 45 | 2.8921 | 2.2139 | -0.38552738 |
| miR1211 | AGGGATGGGAATGGTATATGCAAA | 32 | 23 | 1.469 | 1.1316 | -0.37647031 |
| miR1073 | TGGGCAGAATTAGATACTTTCATA | 25 | 18 | 1.1477 | 0.8856 | -0.37401845 |
| miR1124 | GCAGGACGATGAAGAGGAGCTGC | 37 | 27 | 1.6985 | 1.3284 | -0.35457159 |
| miR5835 | ACAAACTCATCCGGATTTTGGCAT | 119 | 87 | 5.4628 | 4.2803 | -0.35192869 |
| miR5164 | CGCATACTTGTAGTCTAGATTACC | 172 | 126 | 7.8959 | 6.199 | -0.34906821 |
| miR393 | ATCATGCTATCCCTTTGGATT | 103 | 76 | 4.7283 | 3.7391 | -0.33863052 |
| miR6034 | CTGATATGTAGAGGATTGGATTGT | 159 | 118 | 7.2991 | 5.8054 | -0.33032311 |
| miR5766 | TTGTGGATGAGGAAGAGGAAG | 586 | 435 | 26.901 | 21.4014 | -0.32995463 |
| miR5671 | CATGGTGGTGACGGGTGAC | 2644 | 1976 | 121.376 | 97.2165 | -0.32020889 |
| miR1312 | TCAATGGAGAAGAAAATGGGACAT | 33 | 25 | 1.5149 | 1.23 | -0.30056425 |
| miR397 | TCATTGAGTGCAGCGTTGATG | 5109 | 3874 | 234.535 | 190.595 | -0.29928747 |
| miR5227 | TGAAGATGAAGATGTTGATGA | 2086 | 1590 | 95.7603 | 78.2258 | -0.29178316 |
| miR2089 | TTACCTATGCCACCCATTCCT | 1994 | 1527 | 91.5369 | 75.1263 | -0.28503538 |
| miR2118 | GTCGATGGAACAATGTAGGCAAGG | 42964 | 32945 | 1972.31 | 1620.85 | -0.28313903 |
| miR6432 | CTGACTTGGAGAAAAAGATGG | 175 | 135 | 8.0336 | 6.6418 | -0.27447235 |
| miR1513 | TTTAATGTGTAGAGATTCAATGGT | 158 | 122 | 7.2532 | 6.0022 | -0.27312624 |
| miR6478 | CCGACCTTAGCTCAGTTGGC | 2511 | 1952 | 115.27 | 96.0357 | -0.26337938 |
| miR1028 | TGCACTTGTAGGGTTTAAGGAGGC | 27 | 21 | 1.2395 | 1.0332 | -0.26263872 |
| miR1114 | AAGAGAGGACAAAACATTGGCACG | 156 | 122 | 7.1614 | 6.0022 | -0.25475026 |
| miR844 | AGAGAGACTAAAGATGGCT | 463 | 364 | 21.2546 | 17.9083 | -0.24714672 |
| miR447 | TAAGTGGAAGAAATTGTGTTT | 136 | 107 | 6.2432 | 5.2643 | -0.24604398 |
| miR8022 | TTTAAGATAGATTTTGGATTTT | 38 | 30 | 1.7444 | 1.476 | -0.24103817 |
| miR6441 | AATTGACGGAAGGGCACA | 22765 | 17982 | 1045.05 | 884.689 | -0.24033427 |
| miR399 | TGCCAAAGGAGAGTTGCCCTA | 62 | 49 | 2.8462 | 2.4107 | -0.23958492 |
| miR5524 | GAAAAATGTGGATTCATGACGG | 1070 | 851 | 49.1196 | 41.868 | -0.23045081 |
| miR8043 | TTTATAGTTGGACTTTGGCCG | 221 | 176 | 10.1453 | 8.659 | -0.2285392 |
| miR8142 | TGAGATAGTGGAAGTTAAAGGTT | 118 | 94 | 5.4169 | 4.6247 | -0.22810768 |
| miR5024 | ATAAGAACGGCCAAGATACTAACA | 86 | 69 | 3.9479 | 3.3947 | -0.21780136 |
| miR5790 | AACGAGATTCTAGCAAAGCTTCA | 31 | 25 | 1.4231 | 1.23 | -0.21037873 |
| miR1088 | CAGAAAGAGCAGTGAGCACGCAT | 109 | 88 | 5.0038 | 4.3295 | -0.2088237 |
| miR1879 | GTTTGTTTGGTTTGGGAGAGATGG | 182 | 147 | 8.3549 | 7.2322 | -0.20818799 |
| miR5669 | AATGTGAGTGTGGTTAAGTGGGGC | 54 | 44 | 2.4789 | 2.1647 | -0.19553297 |
| miR6260 | TGAGTGAGAGATTGGGTGT | 1021 | 832 | 46.8702 | 40.9332 | -0.1953995 |
| miR7711 | ATGAGAATTTCTGAAAGTGAGAAG | 38 | 31 | 1.7444 | 1.5252 | -0.19373246 |
| miR5265 | AAGTGATGTGTGGATGATGGTTGA | 276 | 226 | 12.6701 | 11.1189 | -0.18841384 |
| miR6462 | AAGGGACTAAAATGGGCATTAAGC | 74 | 61 | 3.3971 | 3.0011 | -0.1788123 |
| miR1023 | AGACTGAGAATTGAAGAGAGTGCA | 441 | 364 | 20.2446 | 17.9083 | -0.17690875 |
| miR2101 | ATTTGAACTCAAGTGACCGTTGGT | 104 | 86 | 4.7742 | 4.2311 | -0.17422622 |
| miR1445 | TCCCTTGTAGATCTAGTAGAAGA | 2297 | 1906 | 105.447 | 93.7726 | -0.16927287 |
| miR3954 | TTGGACAGAGAAATCACGGTCA | 1E+06 | 806756 | 44566.3 | 39691.3 | -0.16713125 |
| miR5250 | TGATGCATGTTGATACGGATC | 811 | 674 | 37.2299 | 33.1599 | -0.16702208 |
| miR5657 | TGGACAAGGAAGATTGAGGTG | 479 | 399 | 21.9891 | 19.6302 | -0.16371369 |
| miR4371 | ATGTGAATGAAGTGGTAGAGGAGT | 72 | 60 | 3.3052 | 2.9519 | -0.16309372 |
| miR8129 | ATAATAAATCGTCCGGCATGCAAT | 30 | 25 | 1.3772 | 1.23 | -0.16307977 |
| miR1115 | TCACCAAAATCCTGATGGCTCA | 24 | 20 | 1.1017 | 0.984 | -0.1630012 |
| miR5376 | TGAGAGGGTTTGAAGAATTTGGGC | 1325 | 1109 | 60.8257 | 54.5613 | -0.156803 |
| miR6438 | TTTACAATCAGAATAGGTAGAATT | 86 | 72 | 3.9479 | 3.5423 | -0.15639905 |
| miR1857 | TGGATTTTTTTGAAGCATCGG | 25 | 21 | 1.1477 | 1.0332 | -0.15162603 |
| miR4349 | TATGGCAGATGATAGAGACAAAGT | 65 | 55 | 2.9839 | 2.7059 | -0.14109066 |
| miR3447 | ATTTGAGTGTCGTTTGGTTTGAA | 26 | 22 | 1.1936 | 1.0824 | -0.1410857 |
| miR1108 | TGAGCAAATGAGAGAATGAACCCC | 39 | 33 | 1.7903 | 1.6236 | -0.14100511 |
| miR1108 | AAGCTAAGAGGAACATACTGAACC | 39 | 33 | 1.7903 | 1.6236 | -0.14100511 |
| miR3946 | GTAGAGAGAGAGAGAGAGAGCAA | 1074 | 911 | 49.3032 | 44.8199 | -0.13754186 |
| miR5713 | TATGAGCTTCAGAAGAACTTTGTT | 320 | 272 | 14.69 | 13.382 | -0.13454065 |
| miR2604 | TTAATTTTTTTGTGAGAGTGT | 28 | 24 | 1.2854 | 1.1808 | -0.12245275 |
| miR5644 | GTGAAGTGGCGGATAACGGTA | 163 | 140 | 7.4827 | 6.8878 | -0.11951568 |
| miR5073 | GTTTGGTGGACGGTAAATATATTT | 848 | 729 | 38.9284 | 35.8658 | -0.11821424 |
| miR3704 | GGATCTGGTGGAGATTGTGGAGGA | 94 | 81 | 4.3152 | 3.9851 | -0.1148115 |
| miR5167 | TCTAGTTAACGGTAATTTGACAGT | 29 | 25 | 1.3313 | 1.23 | -0.11417739 |
| miR415 | AAAAGATGCAGAAGGCAGAACAT | 102 | 88 | 4.6824 | 4.3295 | -0.11304776 |
| miR1026 | ATGAGAAAGATAGAGAGGACG | 73 | 63 | 3.3511 | 3.0995 | -0.11259923 |
| miR847 | CTTGAATGTAAGTATGGAATGGAA | 514 | 445 | 23.5958 | 21.8934 | -0.10803407 |
| miR4344 | AAGTAGACATTTAATGACGGTTTTT | 60 | 52 | 2.7544 | 2.5583 | -0.10655263 |
| miR403 | TTAGATTCACGCACAAACTCG | 463 | 402 | 21.2546 | 19.7778 | -0.10389315 |
| miR5021 | TGGGCCAAGAAGAAGAAAGAAAA | 138 | 120 | 6.3351 | 5.9038 | -0.10172354 |
| miR3952 | TGAAGGGCCTTTCTAGAGCAC | 215 | 187 | 9.8698 | 9.2001 | -0.10137131 |
| miR440 | ATGTCACTGATGATCGGAGGACAA | 371 | 324 | 17.0312 | 15.9404 | -0.09549226 |
| miR6019 | AACAGGTGGACGGTTGTAAATTTT | 2533 | 2213 | 116.28 | 108.877 | -0.0949141 |
| miR6286 | TTTGCACACTATTGGATCGTCGTT | 41 | 36 | 1.8822 | 1.7712 | -0.08769281 |
| miR171 | CGAGCCGAATCAATATCACTC | 19696 | 17351 | 904.168 | 853.645 | -0.08295414 |
| miR2199 | TGATAACTCGACGGATCGC | 11548 | 10175 | 530.124 | 500.596 | -0.08268447 |
| miR5263 | AGGCTAAAACAGAACGGGG | 132 | 117 | 6.0596 | 5.7562 | -0.07410584 |
| miR5721 | AGAAAATGGTAGAGAGAAAGTGGA | 567 | 503 | 26.0288 | 24.7469 | -0.07286099 |
| miR162 | TCGATAAACCTCTGCATCCAG | 670 | 595 | 30.7571 | 29.2732 | -0.07133902 |
| miR858 | TGTACGACAGATAACGAAGAT | 531 | 474 | 24.3762 | 23.3201 | -0.06389927 |
| miR6466 | TCAGTGGTAGAGCATTTGACTGCA | 737 | 658 | 33.8328 | 32.3727 | -0.06364488 |
| miR5139 | AACCTGGCTCTGATACCA | 1498 | 1338 | 68.7674 | 65.8277 | -0.06303001 |
| miR156 | TGACAGAAGAGAGTGAGCAC | 4E+05 | 313995 | 16099.2 | 15448.1 | -0.05955373 |
| miR7122 | TTGGACAGAGAAATCACGGTCG | 10843 | 9719 | 497.761 | 478.161 | -0.05795438 |
| miR4393 | TTGAATAAGGGACACAGAGAC | 435 | 390 | 19.9692 | 19.1875 | -0.05760979 |
| miR6295 | AGGACAGGAGATGATTCATGA | 920 | 825 | 42.2337 | 40.5889 | -0.0573094 |
| miR5656 | AGTGAGTGAGAGATTGGGTGT | 427 | 383 | 19.6019 | 18.8431 | -0.05695717 |
| miR3514 | AGGATTCTGGAATTAAACGGTGAA | 215 | 193 | 9.8698 | 9.4953 | -0.05580727 |
| miR3515 | GAATGTAGAGCAAAATGAAGGTAT | 315 | 283 | 14.4604 | 13.9232 | -0.05461663 |
| miR5655 | AAGTAGAGACTGATGAAGAAGGAG | 79 | 71 | 3.6266 | 3.4931 | -0.05410969 |
| miR5740 | TGGAACAGGAAACAACATTTGG | 237 | 213 | 10.8798 | 10.4793 | -0.05410969 |
| miR831 | AGAGAATTACAGAGGATGATGAGA | 258 | 232 | 11.8438 | 11.4141 | -0.05331493 |
| miR818 | GAGGAGCATTAGGATGGACCA | 745 | 671 | 34.2001 | 33.0123 | -0.05099689 |
| miR1515 | TCATTTTTGCGTGCAATGATCC | 297 | 268 | 13.6341 | 13.1852 | -0.04830001 |
| miR164 | TGGAGAAGCAGGGCACGTGCA | 18977 | 17132 | 871.161 | 842.871 | -0.04762854 |
| miR1523 | AGTGGGATAAATGTAAGGCTA | 212 | 192 | 9.7321 | 9.4461 | -0.04303234 |
| miR6217 | AAAATATATCGTAAGTAGGGGTGG | 43 | 39 | 1.974 | 1.9187 | -0.04099283 |
| miR8141 | TGGAACTGAGGCTGGTAT | 164 | 149 | 7.5286 | 7.3306 | -0.03845032 |
| miR3699 | TCAGAAGATAGAGTTTGGTCA | 22 | 20 | 1.0099 | 0.984 | -0.03748222 |
| miR3629 | GGCTTTGTAAAATGTAGGA | 589 | 536 | 27.0387 | 26.3705 | -0.03610086 |
| miR6198 | GCTGTTCTTAGAGATGGTCGATTC | 106 | 97 | 4.8661 | 4.7723 | -0.02808123 |
| miR477 | ACCTCCCTCGAAGGCTTCCAA | 15408 | 14114 | 707.322 | 694.389 | -0.02662283 |
| miR5719 | TTGTGATGAAAATAGACGTCC | 2640 | 2419 | 121.192 | 119.011 | -0.02619826 |
| miR7496 | AGACCAAATTGTTAGACGATGTGT | 638 | 585 | 29.2881 | 28.7812 | -0.02518786 |
| miR6140 | AAGTTTGTAGAAGAGTTTGTGGCT | 1457 | 1337 | 66.8853 | 65.7785 | -0.02407306 |
| miR7746 | AAGACAAGACTTCGCAACAGAATG | 25 | 23 | 1.1477 | 1.1316 | -0.0203815 |
| miR5718 | ACAGAGACACAAACACAGACACAA | 458 | 422 | 21.025 | 20.7618 | -0.01817427 |
| miR2084 | CCTCATTGATGGATTGTGTAA | 26 | 24 | 1.1936 | 1.1808 | -0.01555481 |
| miR8148 | TAGACGGATCGATGACGTGGCAT | 1981 | 1829 | 90.9401 | 89.9843 | -0.01524328 |
| miR3947 | AATGATTTAGTAGACGACGTTACA | 278 | 257 | 12.7619 | 12.644 | -0.01339019 |
| miR4403 | ACGACACGAACACGACCCGATGAC | 2178 | 2020 | 99.9836 | 99.3812 | -0.00871851 |
| miR894 | GTTTCACGTCGGGTTCACCA | 21588 | 20041 | 991.022 | 985.989 | -0.00734524 |
| miR419 | TTGATGAATGGCTAGGATTTG | 911 | 849 | 41.8205 | 41.7696 | -0.00175698 |

* and ** indicate a significant difference at *P* < 0.05 and *P* < 0.01, respectively.
