# Supplementary material for: MicroRNA-mediated responses to long-term magnesium-deficiency in Citrus sinensis roots revealed by Illumina sequencing
Source: BMC Genomics. 2017 Aug 24;18:657. doi: 10.1186/s12864-017-3999-5 (PMC5571589; doi:10.1186/s12864-017-3999-5)
Supplement: Supplementary file 2 — List of known miRNAs in C. sinensis roots after removing these miRNAs with normalized read-count less than 10 TPM in the two miRNA libraries constructed from Mg-sufficient and -deficient roots. (DOC 447 kb) [file 12864_2017_3999_MOESM2_ESM.doc]

**Additional file 2** List of known miRNAs in *C. sinensis* roots after removing these miRNAs with normalized read-count less than 10 TPM in the two miRNA libraries constructed from Mg-sufficient and -deficient roots.

| miRNA | Sequence | Expressed | | Normalized read count | | Fold change |
| --- | --- | --- | --- | --- | --- | --- |
|  |  | Control | Mg-  deficiency | Control | Mg-  deficiency |  |
| **Up-regulated miRNAs** | |  |  |  |  |  |
| miR6108 | AGGTGAGAAGGGTGATCT | 0 | 2835 | 0.01 | 139.4781 | 13.767751** |
| miR8032 | AGTGTGGAGTGGGAGTGTGAGTAGG | 0 | 2618 | 0.01 | 128.802 | 13.65286737** |
| miR5243 | TGGGCAGAGTATTCGGTGAGC | 0 | 2542 | 0.01 | 125.0629 | 13.61036626** |
| miR3981 | AGTATTCAAGATCGTCTCAT | 0 | 1448 | 0.01 | 71.2396 | 12.7984637** |
| miR2870 | ACTAACAGTTTGGTGGACGACAAA | 0 | 881 | 0.01 | 43.344 | 12.08161658** |
| miR8128 | TCAGCGGGGAAAGACTAATCG | 0 | 870 | 0.01 | 42.8028 | 12.06348946** |
| miR5513 | AAACAAAGGAAACAGACA | 0 | 703 | 0.01 | 34.5866 | 11.75599748** |
| miR5141 | AGACCAGACGTGATGAGCAGATAA | 0 | 683 | 0.01 | 33.6027 | 11.71436144** |
| miR1851 | TATGGGATGGCATTTGGC | 0 | 580 | 0.01 | 28.5352 | 11.47852696** |
| miR917 | TTTGCACGGTTATTTTTGAA | 0 | 559 | 0.01 | 27.502 | 11.42532082** |
| miR5062 | TGAACCTCTGGAGAAGAAGCCCCT | 0 | 425 | 0.01 | 20.9094 | 11.02993595** |
| miR7725 | AAACGAGATGGGACGAGAT | 0 | 407 | 0.01 | 20.0238 | 10.96750007** |
| miR8138 | TAAAGATGGGAACAAAACAA | 0 | 404 | 0.01 | 19.8762 | 10.95682625** |
| miR5338 | TGAAGCTCAGTTGGTAGGTTT | 0 | 397 | 0.01 | 19.5318 | 10.9316092** |
| miR5525 | TCAATCCTTGTGGAGACGATCTGA | 0 | 366 | 0.01 | 18.0067 | 10.81431809** |
| miR1078 | CTTGATTGATTGTTGGAT | 1 | 1594 | 0.0459 | 78.4226 | 10.7385596** |
| miR2878 | TACATGTTAAAATTTTGTAGTGAT | 0 | 328 | 0.01 | 16.1371 | 10.65616562** |
| miR8130 | GGGTTCAATTGTGGAAGGCT | 0 | 308 | 0.01 | 15.1532 | 10.56540677** |
| miR2631 | TGACACCTACGATGGCACACC | 0 | 267 | 0.01 | 13.136 | 10.35931032** |
| miR160 | TGCCTGGCTCCCTGTATGCCA | 0 | 262 | 0.01 | 12.89 | 10.33203655** |
| miR1853 | TAATTCGGGTTATGTTCGGATTGT | 0 | 258 | 0.01 | 12.6932 | 10.30984011** |
| miR2876 | AATGGTGGCTGCGACTGTTTA | 0 | 250 | 0.01 | 12.2997 | 10.26440741** |
| miR1432 | ACATGGAGAGGACACCGAC | 0 | 231 | 0.01 | 11.3649 | 10.15036927** |
| miR4410 | TATGTTGTCCGTCATGATCGTGAA | 0 | 226 | 0.01 | 11.1189 | 10.11879835** |
| miR7488 | TTTTGATGTAGCAGGGGAAACAA | 0 | 207 | 0.01 | 10.1841 | 9.99210278** |
| miR158 | TCTTAAATGTAGACAAAGCA | 1 | 563 | 0.0459 | 27.6988 | 9.23711361** |
| miR6150 | CTTGTTTGATGGTATTTGCT | 9 | 4179 | 0.4132 | 205.601 | 8.95879131** |
| miR6278 | TCATTGTACACAAGCTGAG | 5 | 2162 | 0.2295 | 106.3674 | 8.85634619** |
| miR1077 | TTGAAGTGTTCGGATCGCGGC | 78 | 31368 | 3.5807 | 1543.262 | 8.75152564** |
| miR779 | TCTGCTCATAGATTGTCTGCTCAT | 1 | 316 | 0.0459 | 15.5468 | 8.40390779** |
| miR1168 | TGTGGACAAGGCCAAGGAA | 13 | 2480 | 0.5968 | 122.0126 | 7.67556689** |
| miR7730 | ATGAACACGACACGATTGAAGTTAT | 4 | 760 | 0.1836 | 37.3909 | 7.66997733** |
| miR7785 | GTAGGTGGGTAGAGAGAGAAGGC | 3 | 457 | 0.1377 | 22.4838 | 7.35121352** |
| miR1512 | ATAAATGGAAATTGGGATCAATGA | 5 | 693 | 0.2295 | 34.0946 | 7.2149053** |
| miR4345 | AATAGACGGAACTTACTAAGAT | 52 | 6630 | 2.3871 | 326.1868 | 7.09429559** |
| miR5558 | TGAGACTTTAGAATTAGAAATGGC | 23 | 2417 | 1.0558 | 118.913 | 6.81542606** |
| miR1440 | TTTAGGAGAGATTGGCTATTAGAG | 9 | 870 | 0.4132 | 42.8028 | 6.69472111** |
| miR5149 | GAGAGGCTTGTGACGATTTGGG | 4 | 348 | 0.1836 | 17.1211 | 6.54306553** |
| miR5635 | TTTTAGGATTGTAACGGTG | 4 | 320 | 0.1836 | 15.7436 | 6.4220556** |
| miR2928 | AAGAAGAGGACATTGATG | 7 | 481 | 0.3213 | 23.6645 | 6.20265965** |
| miR8744 | TAAAGAGTGGGCAAAATGGT | 8 | 540 | 0.3672 | 26.5673 | 6.17694175** |
| miR1091 | CGGCATGTGAGGGAAGAGTTG | 6 | 364 | 0.2754 | 17.9083 | 6.02295602** |
| miR2919 | AATGGGAGGGGGGGGCAAGAA | 6 | 361 | 0.2754 | 17.7607 | 6.01101607** |
| miR5782 | TAGCGGAAGGAGAAGTCG | 280 | 15688 | 12.8537 | 771.8278 | 5.9080234** |
| miR3520 | AGGTGATCGGTGAATAATTATCCT | 27 | 1236 | 1.2395 | 60.8095 | 5.61646655** |
| miR7745 | AGTAAGGCATTTAGAAAGGAT | 15 | 609 | 0.6886 | 29.962 | 5.44332394** |
| miR5507 | AATGAGAATGGACCGGAG | 9 | 308 | 0.4132 | 15.1532 | 5.19663842** |
| miR5830 | ATGAGAGGAGGTGATGTGACATCA | 7 | 226 | 0.3213 | 11.1189 | 5.11294928** |
| miR7121 | TCCTCTTGGGATCGACACTCGT | 13 | 325 | 0.5968 | 15.9895 | 4.74373348** |
| miR395 | TAACTGAACTGCCTGTAGGGA | 21 | 479 | 0.964 | 23.5661 | 4.61153607** |
| miR6214 | TGACGACGACGACGACGACGACA | 18 | 410 | 0.8263 | 20.1714 | 4.60950174** |
| miR7707 | TTTGAGTCGAAGATGGCTGAATG | 37 | 830 | 1.6985 | 40.8348 | 4.58746604** |
| miR5176 | TGTGATGATGTGGCATTGACCGAT | 34 | 737 | 1.5608 | 36.2594 | 4.53799746** |
| miR5210 | ATAAGTGCGTTTGGAATTAAGGTT | 18 | 352 | 0.8263 | 17.3179 | 4.38945452** |
| miR7821 | AGATGGGCAAGGGCATTTGCA | 64 | 1047 | 2.938 | 51.5109 | 4.13197145** |
| miR3437 | AAAAATACAAGGACTAAACGGAT | 73 | 1070 | 3.3511 | 52.6425 | 3.97352136** |
| miR2643 | TTTGGAGATGAGAAATTATGGTAGA | 31 | 350 | 1.4231 | 17.2195 | 3.5969343** |
| miR3638 | GAACAAGCAGAAAGAGGACACC | 36 | 390 | 1.6526 | 19.1875 | 3.53735727** |
| miR6427 | GTGGAGAATGAAATTATGAAGA | 25 | 254 | 1.1477 | 12.4964 | 3.44469505** |
| miR5542 | TTTGAGAAGGTGATGTGACAT | 77 | 735 | 3.5348 | 36.161 | 3.35473408** |
| miR5304 | AAGATGAGGATGATAGCATTGGA | 73 | 590 | 3.3511 | 29.0272 | 3.11469877** |
| miR414 | TCATCATCATCATCGTCGTCGTC | 30 | 227 | 1.3772 | 11.1681 | 3.01957377** |
| miR7508 | CAAGAGAGAGTAGATCGGGAGAG | 34 | 211 | 1.5608 | 10.3809 | 2.73357394** |
| miR6170 | CAAGAAAAAGTAGAGATGGGCAT | 37 | 217 | 1.6985 | 10.6761 | 2.6520516** |
| miR418 | TTTGTGATGATTGAAATGAGG | 52 | 218 | 2.3871 | 10.7253 | 2.16768709** |
| miR1100 | TCCACGGAAGAACCCCAACTC | 65 | 264 | 2.9839 | 12.9884 | 2.12195263** |
| miR8153 | TGCACTGTAGACTGTAGCGGAGC | 326 | 1259 | 14.9654 | 61.9411 | 2.04926616** |
| miR2119 | CAAAGGGAGGTGGTGGATTAA | 80 | 304 | 3.6725 | 14.9564 | 2.02592856** |
| miR7497 | ATTGTGGACTGTCATATATGGTTA | 64 | 229 | 2.938 | 11.2665 | 1.9391331** |
| miR7485 | AAAGACCATCTTTGATTCGTTTGA | 120 | 347 | 5.5087 | 17.0719 | 1.63183983** |
| miR5818 | TCGAACTGAGAGGCACAGGTT | 362 | 960 | 16.618 | 47.2307 | 1.50697816** |
| miR7545 | TTGAAGAAATTAGAGTGCT | 138 | 326 | 6.3351 | 16.0387 | 1.34011791** |
| miR6464 | ATAATTGATTGTTGGATATTT | 1157 | 2609 | 53.1134 | 128.3592 | 1.27303891** |
| **Down-regulated miRNAs** | |  |  |  |  |  |
| miR6231 | ATATTAGTGGAGTATGGACAT | 7994 | 0 | 366.974 | 0.01 | -15.16338954** |
| miR3710 | CGGGACCTGCACGGGCCACCA | 5218 | 0 | 239.538 | 0.01 | -14.54796795** |
| miR1222 | CTGAAAGATCATTGGTGACA | 2094 | 0 | 96.1275 | 0.01 | -13.23073355** |
| miR6177 | TAGCATGGACAGAAGAGCATA | 1774 | 0 | 81.4375 | 0.01 | -12.99147701** |
| miR780 | TTCTTCTGAAGAACTGGCAT | 1563 | 0 | 71.7513 | 0.01 | -12.80878923** |
| miR5629 | TTAGGATTTAACGGACGTTA | 1043 | 0 | 47.8801 | 0.01 | -12.2252107** |
| miR6151 | AGAGTGTGAGCAATTGGAGAG | 912 | 0 | 41.8664 | 0.01 | -12.0315773** |
| miR6219 | ATCAGGGACGAAAGTTGGG | 841 | 0 | 38.6071 | 0.01 | -11.91465055** |
| miR7714 | CTAAATATGTATGCACGGAGAGC | 619 | 0 | 28.4159 | 0.01 | -11.4724825** |
| miR6229 | ATATCTCACTTGAGCGTCGGAGG | 390 | 0 | 17.9034 | 0.01 | -10.80601794** |
| miR5794 | TGAGGAACACTAGTGGCAT | 365 | 0 | 16.7557 | 0.01 | -10.71043632** |
| miR6469 | CTGGCAAACAGGATCGTTTA | 336 | 0 | 15.4245 | 0.01 | -10.59100808** |
| miR2592 | ACAACAGCGACATCAAGAATATC | 1652 | 1 | 75.837 | 0.0492 | -10.59002795** |
| miR1151 | ACTGGTTGTGGACACGGA | 304 | 0 | 13.9555 | 0.01 | -10.44661802** |
| miR5829 | ATCAGGACTTAGGGATGGTAA | 290 | 0 | 13.3128 | 0.01 | -10.37859829** |
| miR5668 | AGAATCGGAATTATTGACAGC | 261 | 0 | 11.9815 | 0.01 | -10.22659289** |
| miR6425 | TTGCTTCCGTGGACATAGGCA | 256 | 0 | 11.752 | 0.01 | -10.19869057** |
| miR7693 | GACTCTCGCATCGATGAAGAACGTA | 4997 | 4 | 229.393 | 0.1968 | -10.18687598** |
| miR6289 | TCCTTTGAAGGTGTTGGCTGA | 247 | 0 | 11.3388 | 0.01 | -10.14705232** |
| miR169 | AGGCAGTCTCCTTGGCTAAC | 228 | 0 | 10.467 | 0.01 | -10.03157715** |
| miR5502 | CTACGGATCGGATACGGATTC | 221 | 0 | 10.1453 | 0.01 | -9.98659581** |
| miR3438 | TCGATGCTTCATCTCGGACAC | 4764 | 7 | 218.697 | 0.3444 | -9.31063262** |
| miR7708 | TGTCATGAACTGAACGAAAGACG | 311 | 1 | 14.2768 | 0.0492 | -8.18079862** |
| miR5290 | AATGTGAGTAGAGTAGACACCTA | 5595 | 27 | 256.845 | 1.3284 | -7.59506455** |
| miR2665 | TCATTTCAGGAAGAATTGCA | 837 | 7 | 38.4235 | 0.3444 | -6.80175998** |
| miR6247 | TGGCTGAATGAACATAAGGCA | 655 | 6 | 30.0685 | 0.2952 | -6.67041637** |
| miR5271 | TGATAATTCTGGAAAATAACGGTG | 411 | 4 | 18.8674 | 0.1968 | -6.58302159** |
| miR841 | TACGAGCCACTTGAAGATGAACA | 601 | 6 | 27.5896 | 0.2952 | -6.54628801** |
| miR5148 | TAGAGGCCTAGAAATGTCATACT | 380 | 4 | 17.4443 | 0.1968 | -6.46988167** |
| miR5649 | ATTGCAATTGTTGGTTATTTT | 270 | 3 | 12.3947 | 0.1476 | -6.39188682** |
| miR5261 | TGATTTAGATGGCTTTGT | 943 | 13 | 43.2895 | 0.6396 | -6.08070339** |
| miR5029 | AATGACGAGAGAAACTGCA | 546 | 8 | 25.0648 | 0.3936 | -5.99278869** |
| miR3637 | AATATGTTTGTGTTTTCGTCTGA | 1123 | 18 | 51.5526 | 0.8856 | -5.86324616** |
| miR5641 | TTGTAAGTAGATGATGAGAATTA | 5658 | 105 | 259.737 | 5.1659 | -5.65188858** |
| miR394 | AGGTGGGGATGACGTCAAGT | 374 | 8 | 17.1689 | 0.3936 | -5.44692358** |
| miR5198 | GGGAGAAAGAGAGATTGTTGGGAG | 310 | 7 | 14.2309 | 0.3444 | -5.36879795** |
| miR5762 | TCATGAGGAATAGACTGGC | 4231 | 105 | 194.229 | 5.1659 | -5.2325951** |
| miR1044 | TTGTGGGCATATTTCTTTTA | 236 | 6 | 10.8339 | 0.2952 | -5.19771615** |
| miR6480 | TATGCTGAAACGACGGAACAT | 267 | 7 | 12.2569 | 0.3444 | -5.15336519** |
| miR4240 | ATCGGCTAGAGTACAAACCCG | 618 | 18 | 28.37 | 0.8856 | -5.00156712** |
| miR6485 | AGAATGTAGAAGAGGTAA | 695 | 25 | 31.9048 | 1.23 | -4.69704327** |
| miR5834 | TACGGATGAGAAAATGGTGT | 1340 | 50 | 61.5143 | 2.4599 | -4.64425025** |
| miR2616 | AATTCGGTTTGGTTCGGTTCGGAT | 486 | 27 | 22.3104 | 1.3284 | -4.06995485** |
| miR5286 | AAAACGGATGGCAAAGACAGGA | 266 | 18 | 12.211 | 0.8856 | -3.78538232** |
| miR6446 | TGTGGGTGCTTGATGATGGA | 483 | 33 | 22.1727 | 1.6236 | -3.77151631** |
| miR6190 | CGAGAAAAGGAAAAGACAG | 380 | 29 | 17.4443 | 1.4268 | -3.61190068** |
| miR4413 | TAAGAGGATTGTAAGTTACGTG | 1988 | 164 | 91.2614 | 8.0686 | -3.49961451** |
| miR5291 | GTGGATTGATGGATTGGATTGGAT | 634 | 61 | 29.1045 | 3.0011 | -3.27767894** |
| miR8030 | TTCGGGTTCGGTTCGGTTCGGGTT | 283 | 39 | 12.9914 | 1.9187 | -2.75935585** |
| miR6028 | AGGAGATTAAGGACATTAA | 269 | 51 | 12.3488 | 2.5091 | -2.29912898** |
| miR1847 | TGGACTTTGCAGGTTGGGCAC | 686 | 138 | 31.4916 | 6.7894 | -2.21361107** |
| miR6426 | GATGGAGACAGTAGGTGAAGA | 494 | 146 | 22.6776 | 7.183 | -1.65860955** |
| miR812 | AAAAGGATGATAAGTTGGACA | 454 | 135 | 20.8414 | 6.6418 | -1.64980601** |
| miR6171 | ATTGTGGACGGCTGAAGGTTT | 509 | 154 | 23.3662 | 7.5766 | -1.62480064** |
| miR1033 | TGAGGGCGTGATGTGGCAT | 562 | 193 | 25.7993 | 9.4953 | -1.44204643** |
| miR5074 | GCAAGGCCACCGTGCCGGCGACGC | 631 | 218 | 28.9668 | 10.7253 | -1.43338231** |
| miR6449 | CTATGATTCTGGAAATAAACGGTT | 610 | 218 | 28.0028 | 10.7253 | -1.38455309** |
| miR7984 | TCCGACTTTGTGAAATGACTT | 1087 | 440 | 49.9 | 21.6474 | -1.20484606** |
| miR5712 | AATTATTAATTAATTGAGTGGAGA | 310 | 133 | 14.2309 | 6.5434 | -1.12091453** |
| miR7696 | TTCAAATGAGAACTTTGAAG | 16572 | 7497 | 760.757 | 368.842 | -1.04443257** |
| miR5054 | GTTCCCCACAGACGGCGCCA | 15839 | 7207 | 727.108 | 354.5744 | -1.03608069** |
| **Equally expressed miRNAs** | |  |  |  |  |  |
| miR473 | TGAGGCCGTTGGGGAGAGTGG | 1361 | 2478 | 62.4783 | 121.9142 | 0.96443907 |
| miR8125 | CAGGAAAGAGAGAAGAGTA | 197 | 347 | 9.0435 | 17.0719 | 0.9166705 |
| miR398 | GGGGCGACATGAGATCACATG | 988 | 1707 | 45.3553 | 83.982 | 0.888809 |
| miR5266 | CGGGGGACTGACTGGGAACG | 14789 | 24781 | 678.906 | 1219.1908 | 0.84463976 |
| miR5052 | CCTGTGGACGTAGGCATA | 1590 | 2546 | 72.9908 | 125.2597 | 0.77913579 |
| miR8042 | ATTAGACGGAAGTGGATT | 1091 | 1740 | 50.0836 | 85.6056 | 0.77336691 |
| miR396 | GCTCAAGAAAGCTGTGGGAGA | 8151 | 12653 | 374.181 | 622.51 | 0.73436032 |
| miR5211 | TCGCAGGGGAGATGGGACCGC | 10428 | 15729 | 478.71 | 773.845 | 0.69289419 |
| miR5999 | CTTCACGATCATGACGGACAA | 1540 | 2284 | 70.6955 | 112.3696 | 0.6685615 |
| miR408 | ACGGGGAACAGGCAGAGCATG | 12594 | 17902 | 578.142 | 880.7535 | 0.60731392 |
| miR845 | CAAGTGGTATCAGAGCTAAGG | 1269 | 1802 | 58.2549 | 88.6559 | 0.60583724 |
| miR4993 | GGCGGCGGAGGAGGAGGAG | 280 | 394 | 12.8537 | 19.3843 | 0.59270493 |
| miR7814 | AATTGATTTTTATAGCTTTGA | 207 | 286 | 9.5026 | 14.0708 | 0.56631015 |
| miR815 | AAGGGTGATGAGGAGGAGTGGG | 184 | 247 | 8.4467 | 12.1521 | 0.52474593 |
| miR1858 | GTGATGAGGAGGAGTGGGGTC | 240 | 317 | 11.0175 | 15.596 | 0.50137916 |
| miR5562 | GAAGAGGAGAAGGCTGCAC | 337 | 443 | 15.4704 | 21.795 | 0.4944867 |
| miR952 | AAAACAGAACATGGCATTGGT | 2687 | 3496 | 123.35 | 171.9983 | 0.47963776 |
| miR7838 | AGCATGTGCTGGGAGGAGAGAGAG | 633 | 814 | 29.0586 | 40.0477 | 0.46275419 |
| miR5185 | TTTAAAATTGAATCGAGATGC | 263 | 336 | 12.0733 | 16.5307 | 0.45332776 |
| miR5386 | CGTCGGCTGTCGGCGGACTG | 2596 | 3298 | 119.172 | 162.257 | 0.44523057 |
| miR172 | AGAATCTTGATGATGCTGCAT | 61936 | 77687 | 2843.24 | 3822.0925 | 0.42682478 |
| miR3446 | CTCGGAGGCGTAGACGTAGGCAGG | 1865 | 2339 | 85.615 | 115.0756 | 0.42664648 |
| miR157 | TTGACGGAAGATAGAGAGCAC | 138236 | 172840 | 6345.88 | 8503.488 | 0.42223354 |
| miR535 | TGACAATGAGAGAGAGCACAC | 20658 | 25777 | 948.33 | 1268.1926 | 0.41931354 |
| miR479 | TGTGATATTGGTTCGGCTCATC | 61386 | 76396 | 2818 | 3758.5771 | 0.41551719 |
| miR4351 | ATTGGGAGTGTCGAGTGGGAGTGG | 9304 | 11535 | 427.111 | 567.506 | 0.41002493 |
| miR1436 | ACATAATGAGACGGAGAGGAGAT | 367 | 451 | 16.8476 | 22.1886 | 0.39727555 |
| miR5077 | TTCACGTCGGGTTCACCA | 3945 | 4843 | 181.1 | 238.2689 | 0.3958057 |
| miR5373 | TGTCTTGATTTTAGATGCATG | 1404 | 1700 | 64.4523 | 83.6376 | 0.37591982 |
| miR6146 | TTTTGGCACAATAAATACTTAATC | 176 | 212 | 8.0795 | 10.4301 | 0.36841507 |
| miR5565 | TTTTGTTGGAAGATTGTCGGA | 249 | 299 | 11.4306 | 14.7104 | 0.36393534 |
| miR319 | AGCTGCCGACTCATTCATTCA | 493 | 590 | 22.6317 | 29.0272 | 0.35906046 |
| miR1134 | CAGAACAAAGAAGAAGAAGAAGAT | 384 | 458 | 17.628 | 22.533 | 0.3541706 |
| miR390 | TTGGCAGGGAGATAGTGACCA | 16043 | 19034 | 736.473 | 936.4464 | 0.34656457 |
| miR5544 | ATAACTGCGGAGTAGAAGTTGG | 303 | 355 | 13.9096 | 17.4655 | 0.32842701 |
| miR4414 | TGTGAATGATGCGGGAGATAA | 789 | 923 | 36.22 | 45.4103 | 0.32623302 |
| miR482 | AATGGGAGGCTTGGCAAGAAG | 7917 | 9205 | 363.439 | 452.8732 | 0.31739353 |
| miR6484 | TGATGGGCTCTGCAAGAATGG | 440 | 491 | 20.1987 | 24.1565 | 0.258149 |
| miR5539 | AAGAAAACGGGATGGCGAGCT | 1464 | 1616 | 67.2066 | 79.505 | 0.24244267 |
| miR3948 | GGAGTGGGAGTGGGAGTAGGTTGT | 13095 | 14421 | 601.141 | 709.4932 | 0.23908496 |
| miR1886 | TGAGAGAAGTGAGAGAAGTGT | 843 | 923 | 38.6989 | 45.4103 | 0.23072701 |
| miR6118 | TGGACGATATGGGTGGTTCGGAAA | 298 | 324 | 13.68 | 15.9404 | 0.2206196 |
| miR8122 | TAAGGAAGGTTTTGTGGACAAG | 939 | 1019 | 43.1059 | 50.1334 | 0.21788673 |
| miR6475 | TCTTGGAAGTAGAACGACG | 5844 | 6306 | 268.276 | 310.2464 | 0.20969862 |
| miR1510 | AGTGGATAGGTGTAAAACAATACA | 241 | 260 | 11.0634 | 12.7916 | 0.20940191 |
| miR4249 | TAATTATGAGAAGTATGAGCTA | 465 | 500 | 21.3464 | 24.5993 | 0.20462448 |
| miR3950 | TATTTTCTGCAACATGATTGT | 802 | 861 | 36.8167 | 42.36 | 0.20234227 |
| miR5772 | TAGGAATGTGAGTAGAGTAAGCAT | 342 | 367 | 15.6999 | 18.0559 | 0.20171496 |
| miR5234 | TTTTGTTATGGATGGCTGAAG | 731 | 781 | 33.5574 | 38.4241 | 0.19538053 |
| miR1850 | TGGAAAGTAGAAGAGATTGGG | 1042 | 1113 | 47.8342 | 54.7581 | 0.19502992 |
| miR5161 | TTTGATAGAGTGGAGTATA | 3171 | 3357 | 145.568 | 165.1597 | 0.18216449 |
| miR1310 | GAGGCATCGGGGGCGCAACGC | 629 | 665 | 28.875 | 32.7171 | 0.18022393 |
| miR159 | TTTGGATTGAAGGGAGCTCTA | 2108 | 2224 | 96.7702 | 109.4177 | 0.17721139 |
| miR1171 | AGTGTGGAGTGGGAGTGGGAGTGG | 8972 | 9438 | 411.87 | 464.3365 | 0.17298129 |
| miR3951 | TAGATAAAGATGAGAGAAAAA | 38252 | 40126 | 1756 | 1974.1435 | 0.16893196 |
| miR8003 | TTTCTGGTAACAAATGGGAGTC | 205 | 215 | 9.4108 | 10.5777 | 0.16863669 |
| miR165 | TCGGACCAGGCTTCATCCCCC | 1406 | 1471 | 64.5441 | 72.3712 | 0.16513047 |
| miR6233 | CAAGTTGTTTTGGAATTACTGG | 1288 | 1345 | 59.1271 | 66.1721 | 0.16240355 |
| miR5070 | AACTAGTAGGTCAGAGACGT | 511 | 532 | 23.458 | 26.1737 | 0.15803787 |
| miR7129 | AGAAATCTAGAGATCGTGTAT | 2065 | 2144 | 94.7962 | 105.4818 | 0.15409296 |
| miR5237 | TTAAAAGATTGTAAGTGTTGGGAT | 216 | 224 | 9.9157 | 11.0205 | 0.15240315 |
| miR166 | TCGGACCAGGCTTCATTCCCC | 782409 | 810265 | 35917.4 | 39863.9127 | 0.15040063 |
| miR5259 | CAAGGGGTATTTGGATGGACA | 1110 | 1146 | 50.9558 | 56.3816 | 0.14597805 |
| miR6267 | TAGGAATAGGTCAGGCAATGT | 764 | 787 | 35.0723 | 38.7193 | 0.14272083 |
| miR7764 | CAAAACCTTAGATCTGGATCAA | 274 | 281 | 12.5783 | 13.8248 | 0.13632166 |
| miR5770 | TTCAGGATATGGTTTTGATAA | 30478 | 30672 | 1399.13 | 1509.0198 | 0.10908378 |
| miR774 | TGAGATGGAAGATGATGGTAT | 898 | 895 | 41.2237 | 44.0328 | 0.09510459 |
| miR5254 | AGCGGTGGAAGCAATTGTGTA | 4366 | 4327 | 200.426 | 212.8824 | 0.08698485 |
| miR8155 | TAACCTGGCTCTGATACCA | 368 | 362 | 16.8935 | 17.8099 | 0.07621116 |
| miR5244 | TTATCGGATGAAGATTGTTGG | 1047 | 1029 | 48.0638 | 50.6254 | 0.07491069 |
| miR5813 | ACAGCAGGACGGTGGTCATGGA | 3916 | 3848 | 179.769 | 189.3163 | 0.07465839 |
| miR5534 | CTTTAGACAACAGTAGAATGG | 1511 | 1480 | 69.3642 | 72.8139 | 0.07002263 |
| miR4364 | CGTAGATCGGCAGCGGAAGAAGTT | 271 | 265 | 12.4406 | 13.0376 | 0.06762225 |
| miR168 | TCGCTTGGTGCAGGTCGGGAA | 91032 | 88888 | 4178.93 | 4373.1662 | 0.06554469 |
| miR1863 | AGAGTTTGTGGCTGTATCATTACT | 2171 | 2116 | 99.6623 | 104.1043 | 0.06290989 |
| miR2621 | AGCAATTGGGCTAGCGAATTGGGC | 1003 | 975 | 46.0439 | 47.9686 | 0.0590803 |
| miR8126 | TCTGACTCCCAGATTACTGACATA | 228 | 221 | 10.4666 | 10.8729 | 0.05494392 |
| miR1446 | CGAACTCTCTCCCTCAACGGC | 40402 | 39118 | 1854.7 | 1924.5513 | 0.05333566 |
| miR5298 | TGAGGAAATGAATATGAAGACAA | 497 | 481 | 22.8154 | 23.6645 | 0.05271649 |
| miR861 | CTTGGAGAAATTATGAGCGTCAGA | 1521 | 1468 | 69.8233 | 72.2236 | 0.04876179 |
| miR4406 | ACATTGTACTAGAGAACCGGTGTA | 668 | 642 | 30.6653 | 31.5855 | 0.04265535 |
| miR1861 | TGATCTTGAGGCAAGAAGCTGT | 245 | 235 | 11.247 | 11.5617 | 0.03981331 |
| miR5269 | AGAAGATGGTGGGACAACTTGCTT | 416 | 398 | 19.097 | 19.581 | 0.03610843 |
| miR5666 | AGGGACATAGAGACATTTACT | 2944 | 2815 | 135.148 | 138.4941 | 0.03528756 |
| miR6300 | GTCGTTGTAGTATAGTGGT | 12723 | 12142 | 584.064 | 597.3695 | 0.03249687 |
| miR5492 | AGACTAGGAGAAACAGATATGGTT | 712 | 672 | 32.6852 | 33.0615 | 0.01651466 |
| miR1520 | TGTCACGATCCTGTTGGACTAA | 292 | 274 | 13.4046 | 13.4804 | 0.00813514 |
| miR7812 | TGTTAGTGAATTGATGGGTGA | 700 | 655 | 32.1343 | 32.2251 | 0.00407079 |
| miR7532 | GAACAGCCTCTGGTCGATGGA | 4774 | 2290 | 219.156 | 112.6648 | -0.95992133 |
| miR2938 | GATCTTCTGAGAAGGGTTCGAG | 371 | 187 | 17.0312 | 9.2001 | -0.88845864 |
| miR3627 | TCTGTCGCAGGAGAGATGGTGCCTA | 2138 | 1160 | 98.1474 | 57.0704 | -0.78220738 |
| miR5225 | TCTGTCGCAGGAGAGATGGTGC | 2212 | 1213 | 101.544 | 59.6779 | -0.76684201 |
| miR4379 | AGACTGTATACTAGGGAAGGCCT | 218 | 120 | 10.0075 | 5.9038 | -0.76136586 |
| miR2636 | TTTGTGTTGAAGATGGCTGAATAT | 1661 | 933 | 76.2501 | 45.9023 | -0.73217279 |
| miR2936 | GCTAGAGAGAGAGAAGCACGAGAG | 367 | 209 | 16.8476 | 10.2825 | -0.71235202 |
| miR167 | TGAAGCTGCCAGCATGATCTGA | 323037 | 184742 | 14829.4 | 9089.0498 | -0.70625775 |
| miR2916 | GGGGCTCGAAGACGATCAGATA | 5378 | 3194 | 246.883 | 157.1404 | -0.65177511 |
| miR1144 | TGGGTTTATGTGCGGCAGGCAG | 227 | 135 | 10.4207 | 6.6418 | -0.64980601 |
| miR7758 | ATTGACCGTTAGTTGACCGTGTAA | 1228 | 740 | 56.3728 | 36.407 | -0.63078336 |
| miR4391 | TCTATGGCAGAACTAAGAAGAAGA | 620 | 384 | 28.4618 | 18.8923 | -0.59122855 |
| miR8034 | ATATGACAGAAGATCTTCAAAAACT | 798 | 496 | 36.6331 | 24.4025 | -0.58611883 |
| miR472 | TTTTTCCCACACCTCCCATCCC | 672 | 436 | 30.8489 | 21.4506 | -0.52420104 |
| miR5822 | TGTCTGCGAGTCGGGTTG | 1887 | 1225 | 86.6249 | 60.2683 | -0.52338241 |
| miR3633 | TTACCTATGCCACCCATTCCTT | 6158 | 4034 | 282.69 | 198.4672 | -0.51032076 |
| miR5780 | AAACTTAACTGACGGTAGGGA | 3267 | 2144 | 149.975 | 105.4818 | -0.50773178 |
| miR5059 | TCGTTCCTGGGCAGCAACACCA | 7995 | 5384 | 367.02 | 264.8853 | -0.47049011 |
| miR7732 | GTAGAGATGTGGAGGAACAC | 255 | 178 | 11.7061 | 8.7574 | -0.41868599 |
| miR169 | CAGCCAAGGATGACTTGCCGG | 802 | 561 | 36.8167 | 27.6004 | -0.41567114 |
| miR6280 | TTAGCATGTAAGATTCTTGGT | 222 | 157 | 10.1912 | 7.7242 | -0.39986651 |
| miR393 | ATCATGCTATCCCTTTGGATT | 586 | 435 | 4.7283 | 3.7391 | -0.33863052 |
| miR5766 | TTGTGGATGAGGAAGAGGAAG | 2644 | 1976 | 26.901 | 21.4014 | -0.32995463 |
| miR5671 | CATGGTGGTGACGGGTGAC | 33 | 25 | 121.376 | 97.2165 | -0.32020889 |
| miR397 | TCATTGAGTGCAGCGTTGATG | 5109 | 3874 | 234.535 | 190.5954 | -0.29928747 |
| miR5227 | TGAAGATGAAGATGTTGATGA | 2086 | 1590 | 95.7603 | 78.2258 | -0.29178316 |
| miR2089 | TTACCTATGCCACCCATTCCT | 1994 | 1527 | 91.5369 | 75.1263 | -0.28503538 |
| miR2118 | GTCGATGGAACAATGTAGGCAAGG | 42964 | 32945 | 1972.31 | 1620.8482 | -0.28313903 |
| miR6478 | CCGACCTTAGCTCAGTTGGC | 2511 | 1952 | 115.27 | 96.0357 | -0.26337938 |
| miR844 | AGAGAGACTAAAGATGGCT | 463 | 364 | 21.2546 | 17.9083 | -0.24714672 |
| miR6441 | AATTGACGGAAGGGCACA | 22765 | 17982 | 1045.05 | 884.6894 | -0.24033427 |
| miR5524 | GAAAAATGTGGATTCATGACGG | 1070 | 851 | 49.1196 | 41.868 | -0.23045081 |
| miR8043 | TTTATAGTTGGACTTTGGCCG | 221 | 176 | 10.1453 | 8.659 | -0.2285392 |
| miR6260 | TGAGTGAGAGATTGGGTGT | 1021 | 832 | 46.8702 | 40.9332 | -0.1953995 |
| miR5265 | AAGTGATGTGTGGATGATGGTTGA | 276 | 226 | 12.6701 | 11.1189 | -0.18841384 |
| miR1023 | AGACTGAGAATTGAAGAGAGTGCA | 441 | 364 | 20.2446 | 17.9083 | -0.17690875 |
| miR1445 | TCCCTTGTAGATCTAGTAGAAGA | 2297 | 1906 | 105.447 | 93.7726 | -0.16927287 |
| miR3954 | TTGGACAGAGAAATCACGGTCA | 970813 | 806756 | 44566.3 | 39691.2748 | -0.16713125 |
| miR5250 | TGATGCATGTTGATACGGATC | 811 | 674 | 37.2299 | 33.1599 | -0.16702208 |
| miR5657 | TGGACAAGGAAGATTGAGGTG | 479 | 399 | 21.9891 | 19.6302 | -0.16371369 |
| miR5376 | TGAGAGGGTTTGAAGAATTTGGGC | 72 | 60 | 60.8257 | 54.5613 | -0.156803 |
| miR3946 | GTAGAGAGAGAGAGAGAGAGCAA | 1325 | 1109 | 49.3032 | 44.8199 | -0.13754186 |
| miR5713 | TATGAGCTTCAGAAGAACTTTGTT | 1074 | 911 | 14.69 | 13.382 | -0.13454065 |
| miR5073 | GTTTGGTGGACGGTAAATATATTT | 320 | 272 | 38.9284 | 35.8658 | -0.11821424 |
| miR847 | CTTGAATGTAAGTATGGAATGGAA | 514 | 445 | 23.5958 | 21.8934 | -0.10803407 |
| miR403 | TTAGATTCACGCACAAACTCG | 463 | 402 | 21.2546 | 19.7778 | -0.10389315 |
| miR440 | ATGTCACTGATGATCGGAGGACAA | 371 | 324 | 17.0312 | 15.9404 | -0.09549226 |
| miR6019 | AACAGGTGGACGGTTGTAAATTTT | 2533 | 2213 | 116.28 | 108.8765 | -0.0949141 |
| miR171 | CGAGCCGAATCAATATCACTC | 19696 | 17351 | 904.168 | 853.6451 | -0.08295414 |
| miR2199 | TGATAACTCGACGGATCGC | 11548 | 10175 | 530.124 | 500.5959 | -0.08268447 |
| miR5721 | AGAAAATGGTAGAGAGAAAGTGGA | 567 | 503 | 26.0288 | 24.7469 | -0.07286099 |
| miR162 | TCGATAAACCTCTGCATCCAG | 670 | 595 | 30.7571 | 29.2732 | -0.07133902 |
| miR858 | TGTACGACAGATAACGAAGAT | 531 | 474 | 24.3762 | 23.3201 | -0.06389927 |
| miR6466 | TCAGTGGTAGAGCATTTGACTGCA | 737 | 658 | 33.8328 | 32.3727 | -0.06364488 |
| miR5139 | AACCTGGCTCTGATACCA | 1498 | 1338 | 68.7674 | 65.8277 | -0.06303001 |
| miR156 | TGACAGAAGAGAGTGAGCAC | 350697 | 313995 | 16099.2 | 15448.1179 | -0.05955373 |
| miR7122 | TTGGACAGAGAAATCACGGTCG | 10843 | 9719 | 497.761 | 478.1613 | -0.05795438 |
| miR4393 | TTGAATAAGGGACACAGAGAC | 435 | 390 | 19.9692 | 19.1875 | -0.05760979 |
| miR6295 | AGGACAGGAGATGATTCATGA | 920 | 825 | 42.2337 | 40.5889 | -0.0573094 |
| miR5656 | AGTGAGTGAGAGATTGGGTGT | 427 | 383 | 19.6019 | 18.8431 | -0.05695717 |
| miR3515 | GAATGTAGAGCAAAATGAAGGTAT | 315 | 283 | 14.4604 | 13.9232 | -0.05461663 |
| miR5655 | AAGTAGAGACTGATGAAGAAGGAG | 79 | 71 | 3.6266 | 3.4931 | -0.05410969 |
| miR5740 | TGGAACAGGAAACAACATTTGG | 237 | 213 | 10.8798 | 10.4793 | -0.05410969 |
| miR831 | AGAGAATTACAGAGGATGATGAGA | 258 | 232 | 11.8438 | 11.4141 | -0.05331493 |
| miR818 | GAGGAGCATTAGGATGGACCA | 745 | 671 | 34.2001 | 33.0123 | -0.05099689 |
| miR1515 | TCATTTTTGCGTGCAATGATCC | 297 | 268 | 13.6341 | 13.1852 | -0.04830001 |
| miR164 | TGGAGAAGCAGGGCACGTGCA | 18977 | 17132 | 871.161 | 842.8706 | -0.04762854 |
| miR3629 | GGCTTTGTAAAATGTAGGA | 589 | 536 | 27.0387 | 26.3705 | -0.03610086 |
| miR477 | ACCTCCCTCGAAGGCTTCCAA | 15408 | 14114 | 707.322 | 694.3892 | -0.02662283 |
| miR5719 | TTGTGATGAAAATAGACGTCC | 2640 | 2419 | 121.192 | 119.0114 | -0.02619826 |
| miR7496 | AGACCAAATTGTTAGACGATGTGT | 638 | 585 | 29.2881 | 28.7812 | -0.02518786 |
| miR6140 | AAGTTTGTAGAAGAGTTTGTGGCT | 1457 | 1337 | 66.8853 | 65.7785 | -0.02407306 |
| miR5718 | ACAGAGACACAAACACAGACACAA | 458 | 422 | 21.025 | 20.7618 | -0.01817427 |
| miR8148 | TAGACGGATCGATGACGTGGCAT | 1981 | 1829 | 90.9401 | 89.9843 | -0.01524328 |
| miR3947 | AATGATTTAGTAGACGACGTTACA | 278 | 257 | 12.7619 | 12.644 | -0.01339019 |
| miR4403 | ACGACACGAACACGACCCGATGAC | 2178 | 2020 | 99.9836 | 99.3812 | -0.00871851 |
| miR894 | GTTTCACGTCGGGTTCACCA | 21588 | 20041 | 991.022 | 985.9894 | -0.00734524 |
| miR419 | TTGATGAATGGCTAGGATTTG | 911 | 849 | 41.8205 | 41.7696 | -0.00175698 |

* and ** indicate a significant difference at *P* < 0.05 and *P* < 0.01, respectively.
