# Supplementary material for: MicroRNA-mediated responses to long-term magnesium-deficiency in Citrus sinensis roots revealed by Illumina sequencing
Source: BMC Genomics. 2017 Aug 24;18:657. doi: 10.1186/s12864-017-3999-5 (PMC5571589; doi:10.1186/s12864-017-3999-5)
Supplement: Supplementary file 3 — List of Mg-deficiency-responsive known miRNAs in C. sinensis roots. (DOC 205 kb) [file 12864_2017_3999_MOESM3_ESM.doc]

**Additional file 3** List of Mg-deficiency-responsive known miRNAs in *C. sinensis* roots.

| miRNA | Sequence | Expressed | | Normalized read count | | Fold change |
| --- | --- | --- | --- | --- | --- | --- |
|  |  | Control | Mg-  deficiency | Control | Mg-  deficiency |  |
| **Up-regulated miRNAs** | |  |  |  |  |  |
| miR6108 | AGGTGAGAAGGGTGATCT | 0 | 2835 | 0.01 | 139.4781 | 13.767751** |
| miR8032 | AGTGTGGAGTGGGAGTGTGAGTAGG | 0 | 2618 | 0.01 | 128.802 | 13.65286737** |
| miR5243 | TGGGCAGAGTATTCGGTGAGC | 0 | 2542 | 0.01 | 125.0629 | 13.61036626** |
| miR3981 | AGTATTCAAGATCGTCTCAT | 0 | 1448 | 0.01 | 71.2396 | 12.7984637** |
| miR2870 | ACTAACAGTTTGGTGGACGACAAA | 0 | 881 | 0.01 | 43.344 | 12.08161658** |
| miR8128 | TCAGCGGGGAAAGACTAATCG | 0 | 870 | 0.01 | 42.8028 | 12.06348946** |
| miR5513 | AAACAAAGGAAACAGACA | 0 | 703 | 0.01 | 34.5866 | 11.75599748** |
| miR5141 | AGACCAGACGTGATGAGCAGATAA | 0 | 683 | 0.01 | 33.6027 | 11.71436144** |
| miR1851 | TATGGGATGGCATTTGGC | 0 | 580 | 0.01 | 28.5352 | 11.47852696** |
| miR917 | TTTGCACGGTTATTTTTGAA | 0 | 559 | 0.01 | 27.502 | 11.42532082** |
| miR5062 | TGAACCTCTGGAGAAGAAGCCCCT | 0 | 425 | 0.01 | 20.9094 | 11.02993595** |
| miR7725 | AAACGAGATGGGACGAGAT | 0 | 407 | 0.01 | 20.0238 | 10.96750007** |
| miR8138 | TAAAGATGGGAACAAAACAA | 0 | 404 | 0.01 | 19.8762 | 10.95682625** |
| miR5338 | TGAAGCTCAGTTGGTAGGTTT | 0 | 397 | 0.01 | 19.5318 | 10.9316092** |
| miR5525 | TCAATCCTTGTGGAGACGATCTGA | 0 | 366 | 0.01 | 18.0067 | 10.81431809** |
| miR1078 | CTTGATTGATTGTTGGAT | 1 | 1594 | 0.0459 | 78.4226 | 10.7385596** |
| miR2878 | TACATGTTAAAATTTTGTAGTGAT | 0 | 328 | 0.01 | 16.1371 | 10.65616562** |
| miR8130 | GGGTTCAATTGTGGAAGGCT | 0 | 308 | 0.01 | 15.1532 | 10.56540677** |
| miR2631 | TGACACCTACGATGGCACACC | 0 | 267 | 0.01 | 13.136 | 10.35931032** |
| miR160 | TGCCTGGCTCCCTGTATGCCA | 0 | 262 | 0.01 | 12.89 | 10.33203655** |
| miR1853 | TAATTCGGGTTATGTTCGGATTGT | 0 | 258 | 0.01 | 12.6932 | 10.30984011** |
| miR2876 | AATGGTGGCTGCGACTGTTTA | 0 | 250 | 0.01 | 12.2997 | 10.26440741** |
| miR1432 | ACATGGAGAGGACACCGAC | 0 | 231 | 0.01 | 11.3649 | 10.15036927** |
| miR4410 | TATGTTGTCCGTCATGATCGTGAA | 0 | 226 | 0.01 | 11.1189 | 10.11879835** |
| miR7488 | TTTTGATGTAGCAGGGGAAACAA | 0 | 207 | 0.01 | 10.1841 | 9.99210278** |
| miR158 | TCTTAAATGTAGACAAAGCA | 1 | 563 | 0.0459 | 27.6988 | 9.23711361** |
| miR6150 | CTTGTTTGATGGTATTTGCT | 9 | 4179 | 0.4132 | 205.601 | 8.95879131** |
| miR6278 | TCATTGTACACAAGCTGAG | 5 | 2162 | 0.2295 | 106.3674 | 8.85634619** |
| miR1077 | TTGAAGTGTTCGGATCGCGGC | 78 | 31368 | 3.5807 | 1543.262 | 8.75152564** |
| miR779 | TCTGCTCATAGATTGTCTGCTCAT | 1 | 316 | 0.0459 | 15.5468 | 8.40390779** |
| miR1168 | TGTGGACAAGGCCAAGGAA | 13 | 2480 | 0.5968 | 122.0126 | 7.67556689** |
| miR7730 | ATGAACACGACACGATTGAAGTTAT | 4 | 760 | 0.1836 | 37.3909 | 7.66997733** |
| miR7785 | GTAGGTGGGTAGAGAGAGAAGGC | 3 | 457 | 0.1377 | 22.4838 | 7.35121352** |
| miR1512 | ATAAATGGAAATTGGGATCAATGA | 5 | 693 | 0.2295 | 34.0946 | 7.2149053** |
| miR4345 | AATAGACGGAACTTACTAAGAT | 52 | 6630 | 2.3871 | 326.1868 | 7.09429559** |
| miR5558 | TGAGACTTTAGAATTAGAAATGGC | 23 | 2417 | 1.0558 | 118.913 | 6.81542606** |
| miR1440 | TTTAGGAGAGATTGGCTATTAGAG | 9 | 870 | 0.4132 | 42.8028 | 6.69472111** |
| miR5149 | GAGAGGCTTGTGACGATTTGGG | 4 | 348 | 0.1836 | 17.1211 | 6.54306553** |
| miR5635 | TTTTAGGATTGTAACGGTG | 4 | 320 | 0.1836 | 15.7436 | 6.4220556** |
| miR2928 | AAGAAGAGGACATTGATG | 7 | 481 | 0.3213 | 23.6645 | 6.20265965** |
| miR8744 | TAAAGAGTGGGCAAAATGGT | 8 | 540 | 0.3672 | 26.5673 | 6.17694175** |
| miR1091 | CGGCATGTGAGGGAAGAGTTG | 6 | 364 | 0.2754 | 17.9083 | 6.02295602** |
| miR2919 | AATGGGAGGGGGGGGCAAGAA | 6 | 361 | 0.2754 | 17.7607 | 6.01101607** |
| miR5782 | TAGCGGAAGGAGAAGTCG | 280 | 15688 | 12.854 | 771.8278 | 5.9080234** |
| miR3520 | AGGTGATCGGTGAATAATTATCCT | 27 | 1236 | 1.2395 | 60.8095 | 5.61646655** |
| miR7745 | AGTAAGGCATTTAGAAAGGAT | 15 | 609 | 0.6886 | 29.962 | 5.44332394** |
| miR5507 | AATGAGAATGGACCGGAG | 9 | 308 | 0.4132 | 15.1532 | 5.19663842** |
| miR5830 | ATGAGAGGAGGTGATGTGACATCA | 7 | 226 | 0.3213 | 11.1189 | 5.11294928** |
| miR7121 | TCCTCTTGGGATCGACACTCGT | 13 | 325 | 0.5968 | 15.9895 | 4.74373348** |
| miR395 | TAACTGAACTGCCTGTAGGGA | 21 | 479 | 0.964 | 23.5661 | 4.61153607** |
| miR6214 | TGACGACGACGACGACGACGACA | 18 | 410 | 0.8263 | 20.1714 | 4.60950174** |
| miR7707 | TTTGAGTCGAAGATGGCTGAATG | 37 | 830 | 1.6985 | 40.8348 | 4.58746604** |
| miR5176 | TGTGATGATGTGGCATTGACCGAT | 34 | 737 | 1.5608 | 36.2594 | 4.53799746** |
| miR5210 | ATAAGTGCGTTTGGAATTAAGGTT | 18 | 352 | 0.8263 | 17.3179 | 4.38945452** |
| miR7821 | AGATGGGCAAGGGCATTTGCA | 64 | 1047 | 2.938 | 51.5109 | 4.13197145** |
| miR3437 | AAAAATACAAGGACTAAACGGAT | 73 | 1070 | 3.3511 | 52.6425 | 3.97352136** |
| miR2643 | TTTGGAGATGAGAAATTATGGTAGA | 31 | 350 | 1.4231 | 17.2195 | 3.5969343** |
| miR3638 | GAACAAGCAGAAAGAGGACACC | 36 | 390 | 1.6526 | 19.1875 | 3.53735727** |
| miR6427 | GTGGAGAATGAAATTATGAAGA | 25 | 254 | 1.1477 | 12.4964 | 3.44469505** |
| miR5542 | TTTGAGAAGGTGATGTGACAT | 77 | 735 | 3.5348 | 36.161 | 3.35473408** |
| miR5304 | AAGATGAGGATGATAGCATTGGA | 73 | 590 | 3.3511 | 29.0272 | 3.11469877** |
| miR414 | TCATCATCATCATCGTCGTCGTC | 30 | 227 | 1.3772 | 11.1681 | 3.01957377** |
| miR7508 | CAAGAGAGAGTAGATCGGGAGAG | 34 | 211 | 1.5608 | 10.3809 | 2.73357394** |
| miR6170 | CAAGAAAAAGTAGAGATGGGCAT | 37 | 217 | 1.6985 | 10.6761 | 2.6520516** |
| miR418 | TTTGTGATGATTGAAATGAGG | 52 | 218 | 2.3871 | 10.7253 | 2.16768709** |
| miR1100 | TCCACGGAAGAACCCCAACTC | 65 | 264 | 2.9839 | 12.9884 | 2.12195263** |
| miR8153 | TGCACTGTAGACTGTAGCGGAGC | 326 | 1259 | 14.965 | 61.9411 | 2.04926616** |
| miR2119 | CAAAGGGAGGTGGTGGATTAA | 80 | 304 | 3.6725 | 14.9564 | 2.02592856** |
| miR7497 | ATTGTGGACTGTCATATATGGTTA | 64 | 229 | 2.938 | 11.2665 | 1.9391331** |
| miR7485 | AAAGACCATCTTTGATTCGTTTGA | 120 | 347 | 5.5087 | 17.0719 | 1.63183983** |
| miR5818 | TCGAACTGAGAGGCACAGGTT | 362 | 960 | 16.618 | 47.2307 | 1.50697816** |
| **Down-regulated miRNAs** | |  |  |  |  |  |
| miR6231 | ATATTAGTGGAGTATGGACAT | 7994 | 0 | 366.97 | 0.01 | -15.16338954** |
| miR3710 | CGGGACCTGCACGGGCCACCA | 5218 | 0 | 239.54 | 0.01 | -14.54796795** |
| miR1222 | CTGAAAGATCATTGGTGACA | 2094 | 0 | 96.128 | 0.01 | -13.23073355** |
| miR6177 | TAGCATGGACAGAAGAGCATA | 1774 | 0 | 81.438 | 0.01 | -12.99147701** |
| miR780 | TTCTTCTGAAGAACTGGCAT | 1563 | 0 | 71.751 | 0.01 | -12.80878923** |
| miR5629 | TTAGGATTTAACGGACGTTA | 1043 | 0 | 47.88 | 0.01 | -12.2252107** |
| miR6151 | AGAGTGTGAGCAATTGGAGAG | 912 | 0 | 41.866 | 0.01 | -12.0315773** |
| miR6219 | ATCAGGGACGAAAGTTGGG | 841 | 0 | 38.607 | 0.01 | -11.91465055** |
| miR7714 | CTAAATATGTATGCACGGAGAGC | 619 | 0 | 28.416 | 0.01 | -11.4724825** |
| miR6229 | ATATCTCACTTGAGCGTCGGAGG | 390 | 0 | 17.903 | 0.01 | -10.80601794** |
| miR5794 | TGAGGAACACTAGTGGCAT | 365 | 0 | 16.756 | 0.01 | -10.71043632** |
| miR6469 | CTGGCAAACAGGATCGTTTA | 336 | 0 | 15.425 | 0.01 | -10.59100808** |
| miR2592 | ACAACAGCGACATCAAGAATATC | 1652 | 1 | 75.837 | 0.0492 | -10.59002795** |
| miR1151 | ACTGGTTGTGGACACGGA | 304 | 0 | 13.956 | 0.01 | -10.44661802** |
| miR5829 | ATCAGGACTTAGGGATGGTAA | 290 | 0 | 13.313 | 0.01 | -10.37859829** |
| miR5668 | AGAATCGGAATTATTGACAGC | 261 | 0 | 11.982 | 0.01 | -10.22659289** |
| miR6425 | TTGCTTCCGTGGACATAGGCA | 256 | 0 | 11.752 | 0.01 | -10.19869057** |
| miR7693 | GACTCTCGCATCGATGAAGAACGTA | 4997 | 4 | 229.39 | 0.1968 | -10.18687598** |
| miR6289 | TCCTTTGAAGGTGTTGGCTGA | 247 | 0 | 11.339 | 0.01 | -10.14705232** |
| miR169 | AGGCAGTCTCCTTGGCTAAC | 228 | 0 | 10.467 | 0.01 | -10.03157715** |
| miR5502 | CTACGGATCGGATACGGATTC | 221 | 0 | 10.145 | 0.01 | -9.98659581** |
| miR3438 | TCGATGCTTCATCTCGGACAC | 4764 | 7 | 218.7 | 0.3444 | -9.31063262** |
| miR7708 | TGTCATGAACTGAACGAAAGACG | 311 | 1 | 14.277 | 0.0492 | -8.18079862** |
| miR5290 | AATGTGAGTAGAGTAGACACCTA | 5595 | 27 | 256.85 | 1.3284 | -7.59506455** |
| miR2665 | TCATTTCAGGAAGAATTGCA | 837 | 7 | 38.424 | 0.3444 | -6.80175998** |
| miR6247 | TGGCTGAATGAACATAAGGCA | 655 | 6 | 30.069 | 0.2952 | -6.67041637** |
| miR5271 | TGATAATTCTGGAAAATAACGGTG | 411 | 4 | 18.867 | 0.1968 | -6.58302159** |
| miR841 | TACGAGCCACTTGAAGATGAACA | 601 | 6 | 27.59 | 0.2952 | -6.54628801** |
| miR5148 | TAGAGGCCTAGAAATGTCATACT | 380 | 4 | 17.444 | 0.1968 | -6.46988167** |
| miR5649 | ATTGCAATTGTTGGTTATTTT | 270 | 3 | 12.395 | 0.1476 | -6.39188682** |
| miR5261 | TGATTTAGATGGCTTTGT | 943 | 13 | 43.29 | 0.6396 | -6.08070339** |
| miR5029 | AATGACGAGAGAAACTGCA | 546 | 8 | 25.065 | 0.3936 | -5.99278869** |
| miR3637 | AATATGTTTGTGTTTTCGTCTGA | 1123 | 18 | 51.553 | 0.8856 | -5.86324616** |
| miR5641 | TTGTAAGTAGATGATGAGAATTA | 5658 | 105 | 259.74 | 5.1659 | -5.65188858** |
| miR394 | AGGTGGGGATGACGTCAAGT | 374 | 8 | 17.169 | 0.3936 | -5.44692358** |
| miR5198 | GGGAGAAAGAGAGATTGTTGGGAG | 310 | 7 | 14.231 | 0.3444 | -5.36879795** |
| miR5762 | TCATGAGGAATAGACTGGC | 4231 | 105 | 194.23 | 5.1659 | -5.2325951** |
| miR1044 | TTGTGGGCATATTTCTTTTA | 236 | 6 | 10.834 | 0.2952 | -5.19771615** |
| miR6480 | TATGCTGAAACGACGGAACAT | 267 | 7 | 12.257 | 0.3444 | -5.15336519** |
| miR4240 | ATCGGCTAGAGTACAAACCCG | 618 | 18 | 28.37 | 0.8856 | -5.00156712** |
| miR6485 | AGAATGTAGAAGAGGTAA | 695 | 25 | 31.905 | 1.23 | -4.69704327** |
| miR5834 | TACGGATGAGAAAATGGTGT | 1340 | 50 | 61.514 | 2.4599 | -4.64425025** |
| miR2616 | AATTCGGTTTGGTTCGGTTCGGAT | 486 | 27 | 22.31 | 1.3284 | -4.06995485** |
| miR5286 | AAAACGGATGGCAAAGACAGGA | 266 | 18 | 12.211 | 0.8856 | -3.78538232** |
| miR6446 | TGTGGGTGCTTGATGATGGA | 483 | 33 | 22.173 | 1.6236 | -3.77151631** |
| miR6190 | CGAGAAAAGGAAAAGACAG | 380 | 29 | 17.444 | 1.4268 | -3.61190068** |
| miR4413 | TAAGAGGATTGTAAGTTACGTG | 1988 | 164 | 91.261 | 8.0686 | -3.49961451** |
| miR5291 | GTGGATTGATGGATTGGATTGGAT | 634 | 61 | 29.105 | 3.0011 | -3.27767894** |
| miR8030 | TTCGGGTTCGGTTCGGTTCGGGTT | 283 | 39 | 12.991 | 1.9187 | -2.75935585** |
| miR6028 | AGGAGATTAAGGACATTAA | 269 | 51 | 12.349 | 2.5091 | -2.29912898** |
| miR1847 | TGGACTTTGCAGGTTGGGCAC | 686 | 138 | 31.492 | 6.7894 | -2.21361107** |
| miR6426 | GATGGAGACAGTAGGTGAAGA | 494 | 146 | 22.678 | 7.183 | -1.65860955** |
| miR812 | AAAAGGATGATAAGTTGGACA | 454 | 135 | 20.841 | 6.6418 | -1.64980601** |
| miR6171 | ATTGTGGACGGCTGAAGGTTT | 509 | 154 | 23.366 | 7.5766 | -1.62480064** |

** indicates a significant difference at *P* < 0.01.
