# Supplementary material for: MicroRNA-mediated responses to long-term magnesium-deficiency in Citrus sinensis roots revealed by Illumina sequencing
Source: BMC Genomics. 2017 Aug 24;18:657. doi: 10.1186/s12864-017-3999-5 (PMC5571589; doi:10.1186/s12864-017-3999-5)
Supplement: Supplementary file 4 — List of novel miRNAs in C. sinensis roots. (DOC 288 kb) [file 12864_2017_3999_MOESM4_ESM.doc]

**Additional file 4** List of novel miRNAs in *C. sinensis* roots.

| miRNA | *Sequence* | Expressed | | Normalized read count | | Fold change |
| --- | --- | --- | --- | --- | --- | --- |
|  |  | Control | Mg-  deficiency | Control | Mg-  deficiency |  |
| **Up-regulated miRNAs** | |  |  |  |  |  |
| novel_mir_585 | CGTGTCGTGGTGTAGTTGGT | 0 | 62192 | 0.01 | 3059.76 | 18.22305897** |
| novel_mir_586 | AAGACTGTAGTGAACATG | 0 | 5165 | 0.01 | 254.1108 | 14.63317007** |
| novel_mir_429 | TTGGATTGGGTAGAGTATTCGG | 0 | 1120 | 0.01 | 55.1024 | 12.4278994** |
| novel_mir_468 | ATTGGGGGTAGATTGAGGTTT | 0 | 956 | 0.01 | 47.0339 | 12.1994853** |
| novel_mir_470 | TGGGTGGCTTCTCGGACTTAC | 0 | 867 | 0.01 | 42.6552 | 12.0585059** |
| novel_mir_587 | CGGAAGGGCCGCGGCGGC | 0 | 767 | 0.01 | 37.7353 | 11.88169903** |
| novel_mir_504 | TGTGAGATGATTGTAAGTTAC | 0 | 523 | 0.01 | 25.7309 | 11.3292862** |
| novel_mir_397 | CATGGGTGTTAATTGGTTCAAC | 0 | 322 | 0.01 | 15.842 | 10.6295388** |
| novel_mir_497 | TGGCACTCTTCGGACCAATGC | 0 | 305 | 0.01 | 15.0056 | 10.5512853** |
| novel_mir_588 | CTTGTAACTGTAGTAAGGTA | 0 | 282 | 0.01 | 13.874 | 10.43816807** |
| novel_mir_589 | ACTAGTTAGATGGACCTAC | 0 | 269 | 0.01 | 13.2344 | 10.37007707** |
| novel_mir_590 | TCGCGACCCAATGTGATTTTCGGA | 0 | 237 | 0.01 | 11.6601 | 10.18736445** |
| novel_mir_406 | TGGTCGTGTACTTGGACGACAT | 0 | 228 | 0.01 | 11.2173 | 10.1315098** |
| novel_mir_101 | GATGCCTCATCTCGGACACC | 0 | 132 | 0.01 | 6.4942 | 9.34300801** |
| novel_mir_362 | AGGCTGTACCTTACGTTGCAT | 0 | 130 | 0.01 | 6.3958 | 9.32098102** |
| novel_mir_350 | TTGTGGAGTGTGTATGTTACA | 0 | 116 | 0.01 | 5.707 | 9.15658875** |
| novel_mir_361 | TAAACTCGTCTTGGACTTTAA | 0 | 98 | 0.01 | 4.8215 | 8.91333824** |
| novel_mir_155 | TACTAGACACAAGACATGGCAT | 0 | 86 | 0.01 | 4.2311 | 8.72488897** |
| novel_mir_163 | ACGTGGCAGCATATCAGTGGACA | 0 | 79 | 0.01 | 3.8867 | 8.60240195** |
| novel_mir_78 | TTGAGAAGTGTAGTATTATTT | 0 | 75 | 0.01 | 3.6899 | 8.52743791** |
| novel_mir_371 | AGAAAGAAGAAGAAAAATTGA | 0 | 60 | 0.01 | 2.9519 | 8.20550004** |
| novel_mir_591 | ATGTAGAATCAAGGTAAA | 1 | 273 | 0.0459 | 13.4312 | 8.19287834** |
| novel_mir_295 | TTGATGAAAATCTAACGGCTG | 0 | 59 | 0.01 | 2.9027 | 8.18125166** |
| novel_mir_372 | CGGAGTTCATTGTAGAAGGTG | 0 | 59 | 0.01 | 2.9027 | 8.18125166** |
| novel_mir_161 | AATGGCTGTGGTGGGTGGTCT | 0 | 58 | 0.01 | 2.8535 | 8.15658875** |
| novel_mir_434 | TTTCTTCATGAGAGCTGGCCA | 0 | 47 | 0.01 | 2.3123 | 7.85318478** |
| novel_mir_592 | ATTATTGATTGTTAGGAT | 2 | 415 | 0.0918 | 20.4174 | 7.79708929** |
| novel_mir_229 | TGCCAAAGGAGAGTTGCCCTA | 0 | 42 | 0.01 | 2.0663 | 7.69090592** |
| novel_mir_140 | GTGAACCATGGGCCATTTGACGA | 0 | 39 | 0.01 | 1.9187 | 7.58398535** |
| novel_mir_374 | AGAGACGGCACCAGCTGCTTT | 0 | 32 | 0.01 | 1.5744 | 7.29865832** |
| novel_mir_593 | TGATTGATAGGGACAGTTGG | 6 | 868 | 0.2754 | 42.7044 | 7.27671235** |
| novel_mir_437 | CATCGTTGAAATGTAGTAGGTGC | 0 | 29 | 0.01 | 1.4268 | 7.15663931** |
| novel_mir_477 | TCTGTTATGTAAGATGGTCCTA | 0 | 29 | 0.01 | 1.4268 | 7.15663931** |
| novel_mir_345 | ATATTGATATGACTCTTCAAATA | 0 | 28 | 0.01 | 1.3776 | 7.10601324** |
| novel_mir_594 | TAGAGAGAGAGAGAGAGCGAGAG | 7 | 889 | 0.3213 | 43.7376 | 7.08880927** |
| novel_mir_595 | TTTTTTGGATCTGGATATA | 2 | 238 | 0.0918 | 11.7093 | 6.99494496** |
| novel_mir_364 | AACCCTGAATTTGCATTTTAA | 0 | 24 | 0.01 | 1.1808 | 6.88362082** |
| novel_mir_482 | TCTGATTTAGGTTATGGAGGCAT | 0 | 23 | 0.01 | 1.1316 | 6.82222027** |
| novel_mir_423 | GGTGGGCTTGATTTTAGGCAA | 0 | 22 | 0.01 | 1.0824 | 6.75808993** |
| novel_mir_153 | AATTGTTGGGGAAAAATTAGGTT | 0 | 21 | 0.01 | 1.0332 | 6.69097574** |
| novel_mir_596 | TGATTGGGAAGAAGACGACGA | 17 | 688 | 0.7804 | 33.8486 | 5.43873858** |
| novel_mir_597 | TAACTAATCGTGACGGTGACGGTGA | 8 | 284 | 0.3672 | 13.9724 | 5.24986998** |
| novel_mir_598 | ATGTTGTAGGAATGGAGGTAGGTA | 66 | 1946 | 3.0298 | 95.7405 | 4.98183487** |
| novel_mir_599 | TAGTGGGAGATTGTTGGGAAAAT | 8 | 232 | 0.3672 | 11.4141 | 4.95810724** |
| novel_mir_600 | AGCAGATACGGATCTTAAT | 23 | 530 | 1.0558 | 26.0753 | 4.62627538** |
| novel_mir_601 | TGGGGTGGGGATGGGGAAAGCATT | 51 | 734 | 2.3412 | 36.1118 | 3.94715024** |
| novel_mir_602 | TGAGAAAGGAGAGATGGTGCA | 1662 | 16002 | 76.296 | 787.2762 | 3.36719054** |
| novel_mir_603 | ATGAGATGATGATGGATA | 41 | 391 | 1.8822 | 19.2367 | 3.35336949** |
| novel_mir_604 | TGAGAGCTTAGATCAGAAGATGAT | 62 | 557 | 2.8462 | 27.4036 | 3.26725648** |
| novel_mir_605 | TTCATGGAGAACTTGAAGT | 76 | 447 | 3.4889 | 21.9918 | 2.65612154** |
| novel_mir_426 | TTTCTCTTATCGTTATCTGTG | 4689 | 21527 | 215.254 | 1059.0985 | 2.29872482** |
| novel_mir_120 | TTCAAGAAATCTGTGGGAAG | 39 | 151 | 1.7903 | 7.429 | 2.05296667** |
| novel_mir_606 | TGATAGTGACATAGATGATGGATG | 505 | 1746 | 23.1826 | 85.9008 | 1.88962919** |
| novel_mir_381 | TCCAAAGGGATCGCATTGATCC | 10 | 23 | 0.4591 | 1.1316 | 1.30148374** |
| novel_mir_439 | GGGTGGTTGTATTGTGTGGCCC | 71 | 137 | 3.2593 | 6.7402 | 1.04822925** |
|  | **Down-regulated miRNAs** |  |  |  |  |  |
| novel_mir_607 | TTCTCTCAAGTAATTCTGACGGA | 4919 | 0 | 225.8124 | 0.01 | -14.46283864** |
| novel_mir_98 | TCACTACTTTCAATCTCGGTC | 1154 | 0 | 52.9757 | 0.01 | -12.371115** |
| novel_mir_608 | GAGTGAAAGTGGGAGTAGGTTGTT | 999 | 0 | 45.8603 | 0.01 | -12.16303023** |
| novel_mir_609 | GGATGATCGAAAGTAAAAG | 930 | 0 | 42.6927 | 0.01 | -12.05977358** |
| novel_mir_610 | GGGGAGGGGACAAGGATC | 396 | 0 | 18.1788 | 0.01 | -10.82804129** |
| novel_mir_515 | AATGGGAGGGGTGGGCAAGAAA | 174 | 0 | 7.9877 | 0.01 | -9.6416364** |
| novel_mir_584 | TTTTAATTGGTACGTGGCGTTG | 131 | 0 | 6.0137 | 0.01 | -9.2321091** |
| novel_mir_611 | TGGATAGTAGAATAATGAAGGAGA | 1647 | 3 | 75.6074 | 0.1476 | -9.00069089** |
| novel_mir_88 | TTCAGAGTAGACAAATGGTAA | 100 | 0 | 4.5906 | 0.01 | -8.8425389** |
| novel_mir_578 | TTGCTGTATCGGTCGACAACG | 92 | 0 | 4.2234 | 0.01 | -8.7222611** |
| novel_mir_49 | CAACTGTGGAATAGAAGTTGGGA | 85 | 0 | 3.902 | 0.01 | -8.60807** |
| novel_mir_533 | TTGAACTCCTCGAAGCCAGCA | 77 | 0 | 3.5348 | 0.01 | -8.4654848** |
| novel_mir_179 | TCTCTTTCTGAAGATCCTGGAT | 64 | 0 | 2.938 | 0.01 | -8.1986906** |
| novel_mir_137 | AAGCATGGGCTTCGAGGCTTGG | 44 | 0 | 2.0199 | 0.01 | -7.6581401** |
| novel_mir_580 | GCAAGTGTTGGGCATGATCCT | 44 | 0 | 2.0199 | 0.01 | -7.6581401** |
| novel_mir_612 | GAGGAAGGAGAGATGGAGCAG | 13674 | 65 | 627.7209 | 3.1979 | -7.6168546** |
| novel_mir_74 | AGGGATAGTTATAGATGGAGGTT | 38 | 0 | 1.7444 | 0.01 | -7.4465871** |
| novel_mir_522 | AGCAGATGGTGCCATTTCTTC | 32 | 0 | 1.469 | 0.01 | -7.1986906** |
| novel_mir_614 | TGAGACGAGATGGGATGAG | 792 | 6 | 13.68 | 0.0984 | -7.11919419** |
| novel_mir_572 | TTTAAATTTTGTGTTGGATTTA | 30 | 0 | 1.3772 | 0.01 | -7.1055943** |
| novel_mir_287 | CCAAAACTGAAGAAGAGTATT | 28 | 0 | 1.2854 | 0.01 | -7.0060736** |
| novel_mir_527 | ATGCAGCGGAAGTTTAATGTTCT | 28 | 0 | 1.2854 | 0.01 | -7.0060736** |
| novel_mir_582 | ATGAGATGATGATGGATATGA | 27 | 0 | 1.2395 | 0.01 | -6.9536145** |
| novel_mir_613 | TGAAATTTTGGAGGACTT | 298 | 2 | 36.3577 | 0.2952 | -6.9444244** |
| novel_mir_581 | TGACTGAAAGTAACAGTGGTA | 26 | 0 | 1.1936 | 0.01 | -6.8991756** |
| novel_mir_38 | CGGTGCCATCCTCCTGCGATA | 25 | 0 | 1.1477 | 0.01 | -6.8426018** |
| novel_mir_536 | TTTGTGCCATAGCCATTGACA | 25 | 0 | 1.1477 | 0.01 | -6.8426018** |
| novel_mir_517 | TCAAGCTGTCAAGAGGTTCGGTG | 23 | 0 | 1.0558 | 0.01 | -6.7221928** |
| novel_mir_615 | ATGTGGAGAATGAAATTATGAAGA | 225 | 2 | 10.3289 | 0.0984 | -6.71381259** |
| novel_mir_542 | AGGGTAGTGAGTAGAGGAAGACA | 22 | 0 | 1.0099 | 0.01 | -6.6580686** |
| novel_mir_616 | CAGAAAGAAGACGAGTAG | 773 | 17 | 35.4855 | 0.8364 | -5.40689276** |
| novel_mir_617 | ATTTCGATAGTACGAGATTGT | 348 | 11 | 15.9753 | 0.5412 | -4.88353737** |
| novel_mir_618 | TAACTTCAAGTGGAATTCAGCAAA | 264 | 24 | 12.1192 | 1.1808 | -3.35945794** |
| novel_mir_619 | TGGACGGGGTTGATGGGCG | 720 | 84 | 33.0524 | 4.1327 | -2.99959849** |
| novel_mir_276 | AGGCAGTCTCCTTGGCTAAG | 94 | 16 | 4.3152 | 0.7872 | -2.4546253** |
| novel_mir_620 | TGAACAACTGGAGAAGCAA | 1495 | 265 | 68.6297 | 13.0376 | -2.39615473** |
| novel_mir_70 | AGGTGCAGCTGTATATGCAGG | 22 | 9 | 1.0099 | 0.4428 | -1.1894853** |
| novel_mir_9 | TGATTGAGCCGTGCCAATATC | 108 | 48 | 4.9579 | 2.3615 | -1.0700256** |
| novel_mir_205 | TGCCAAAGGAGATTTGCCCGG | 33 | 15 | 1.5149 | 0.738 | -1.0375298** |
|  | **Equally expressed miRNAs** |  |  |  |  |  |
| novel_mir_443 | GCAGCATCATCAAGATTCACA | 1290 | 2366 | 59.2189 | 116.4039 | 0.9750098 |
| novel_mir_430 | GGGGAGAAGATAGGTTTGCTG | 13 | 23 | 0.5968 | 1.1316 | 0.92304464 |
| novel_mir_450 | TCCAATGGCTGTGGTGGGTGG | 92 | 157 | 4.2234 | 7.7242 | 0.87098063 |
| novel_mir_128 | CTGGAGACAACTGTGGTACGG | 410 | 684 | 18.8215 | 33.6519 | 0.83830635 |
| novel_mir_80 | GGAAACCCTAGGGGGAGGTCG | 1738 | 2836 | 79.7849 | 139.5273 | 0.80635979 |
| novel_mir_87 | TGGTATGGGTGAGTAGGGAAG | 24766 | 37340 | 1136.91 | 1837.0761 | 0.6922908 |
| novel_mir_273 | TTTGATATGTGGTTAGCTTGG | 64 | 96 | 2.938 | 4.7231 | 0.68489969 |
| novel_mir_141 | TCTGTATAGGACAGTTGATGTT | 29 | 42 | 1.3313 | 2.0663 | 0.63421402 |
| novel_mir_389 | AAAACTCAGGATAGATAGCGC | 69 | 98 | 3.1675 | 4.8215 | 0.60613743 |
| novel_mir_344 | GATACTCATTTAGGCAAGACG | 577 | 818 | 26.4879 | 40.2445 | 0.60345816 |
| novel_mir_270 | TCTTTATCTGTGGGTGGCATT | 90 | 127 | 4.1316 | 6.2482 | 0.59674005 |
| novel_mir_144 | TTCAACTTTGAAAACGTCATC | 465 | 650 | 21.3464 | 31.9791 | 0.58313655 |
| novel_mir_207 | TGAGGTTCTTGGGGAGAGTAG | 213 | 297 | 9.778 | 14.612 | 0.57954235 |
| novel_mir_59 | CTTTCAGCAGCCTCCGGCGTC | 48 | 65 | 2.2035 | 3.1979 | 0.53732793 |
| novel_mir_266 | TACGTTACAGATTCTGTATGT | 17 | 23 | 0.7804 | 1.1316 | 0.5360784 |
| novel_mir_410 | CCGTTGAGGTAGGGCAGTTCGG | 34496 | 46166 | 1583.58 | 2271.3031 | 0.5203314 |
| novel_mir_401 | CTGGATGCAACTGTGGTACGG | 9198 | 12258 | 422.245 | 603.0766 | 0.51426126 |
| novel_mir_143 | TGGAGAGTGTGCGCTTGTGCC | 109 | 145 | 5.0038 | 7.1338 | 0.51164664 |
| novel_mir_420 | CCGCAGGGGCGACATGAGATC | 1816 | 2411 | 83.3656 | 118.6179 | 0.50879764 |
| novel_mir_436 | TCAACCCAAGATTTGTTCCCA | 16 | 21 | 0.7345 | 1.0332 | 0.49228515 |
| novel_mir_209 | ACCAGCGCTGCACTCGATCAT | 102 | 133 | 4.6824 | 6.5434 | 0.48279228 |
| novel_mir_164 | TGCTAGCGGCAAACCATGACAC | 178 | 222 | 8.1713 | 10.9221 | 0.41861275 |
| novel_mir_376 | TCACAGGATGCTGGCACTTGC | 141 | 175 | 6.4728 | 8.6098 | 0.4115898 |
| novel_mir_129 | TTAGAATTTGTGGTATTGGGC | 65 | 80 | 2.9839 | 3.9359 | 0.39949438 |
| novel_mir_184 | AGTAAGATTGTCGTCACACAT | 671 | 813 | 30.803 | 39.9985 | 0.37687503 |
| novel_mir_20 | GGTCATGGGAGGATTGGCGAGA | 153379 | 183477 | 7041.04 | 9026.8136 | 0.35842803 |
| novel_mir_251 | GGTGCAGCTGTGGTATGGTAC | 61 | 72 | 2.8003 | 3.5423 | 0.33910501 |
| novel_mir_94 | ACTGATGTGGCATGAAGAGAT | 105 | 123 | 4.8201 | 6.0514 | 0.32820587 |
| novel_mir_359 | AATGGGTGCATGGGCAAGAGA | 7642 | 8898 | 350.815 | 437.7692 | 0.31946043 |
| novel_mir_6 | ATACAGAGTCTGTAACGTAGT | 167 | 194 | 7.6663 | 9.5445 | 0.31613917 |
| novel_mir_174 | GTGCTCTCTACCATTGTCATA | 72 | 83 | 3.3052 | 4.0835 | 0.30506865 |
| novel_mir_360 | AGGTCATCTTGCAGCTTCAAT | 184 | 209 | 8.4467 | 10.2825 | 0.28373135 |
| novel_mir_135 | GCAATGCTCTTGAAGGACTAC | 28606 | 32185 | 1313.19 | 1583.4573 | 0.27000043 |
| novel_mir_202 | TATGTTGCAACAGTGGTACGGTA | 72 | 81 | 3.3052 | 3.9851 | 0.26987835 |
| novel_mir_110 | TGCTTGTTGATTGTCATCTAA | 48 | 54 | 2.2035 | 2.6567 | 0.26983843 |
| novel_mir_458 | CGAGGCTTGAACTAGTGCGGT | 2662 | 2988 | 122.202 | 147.0055 | 0.26659987 |
| novel_mir_3 | GGAATGTTGTCTGGCTCGAGG | 4613 | 5152 | 211.765 | 253.4712 | 0.25935699 |
| novel_mir_172 | GCTGTAGAAAGGCCCCTCAAC | 7012 | 7801 | 321.894 | 383.7984 | 0.25376301 |
| novel_mir_467 | TTGATTGTGTGATTTGGGTTG | 19 | 21 | 0.8722 | 1.0332 | 0.24438865 |
| novel_mir_503 | GCTGTAGATAGGCCCTTCAAC | 2071 | 2283 | 95.0717 | 112.3204 | 0.24053212 |
| novel_mir_173 | TCAAGGAGCGCACGAACGGTT | 2451 | 2689 | 112.516 | 132.2951 | 0.23362946 |
| novel_mir_126 | TAAATGATAGTATGATACCCT | 31 | 34 | 1.4231 | 1.6728 | 0.23322792 |
| novel_mir_418 | TGTTTATTTCTTTGGGCGGCTG | 1214 | 1329 | 55.7301 | 65.385 | 0.23050296 |
| novel_mir_472 | GGAATGTTGTTTGGCTCGAGGG | 1238 | 1319 | 56.8318 | 64.893 | 0.19136445 |
| novel_mir_134 | AAGTCATTAGAAGAACTGCCG | 21004 | 22154 | 964.213 | 1089.946 | 0.17683287 |
| novel_mir_442 | TACTTGCTGTATCGGTCGACAA | 447 | 459 | 20.5201 | 22.5822 | 0.13814828 |
| novel_mir_233 | AGCAGGAAAGTGGCTGGTTGA | 442 | 452 | 20.2905 | 22.2378 | 0.13220965 |
| novel_mir_411 | TTTGAACTCCTCGAAGCCTGC | 1120 | 1139 | 51.4149 | 56.0372 | 0.12419836 |
| novel_mir_187 | TGAGGGAAGAGCTTAGAAGG | 188 | 191 | 8.6304 | 9.3969 | 0.12275747 |
| novel_mir_378 | ATGAAAATTCAATGATTGTGGAA | 25 | 25 | 1.1477 | 1.23 | 0.09991273 |
| novel_mir_127 | AGGGACAAGCTAAAAGACCAA | 11367 | 11188 | 521.815 | 550.4341 | 0.0770306 |
| novel_mir_370 | GCTCACTCTCTATCTGTCACC | 130 | 127 | 5.9678 | 6.2482 | 0.06624145 |
| novel_mir_123 | TCGGGTTATAGATGCGGGTTG | 45 | 42 | 2.0658 | 2.0663 | 0.00034914 |
| novel_mir_379 | ACTCTCCCTCAAGGGCTTCGC | 1307 | 639 | 59.9994 | 31.4379 | -0.9324432 |
| novel_mir_19 | TTCCCTAGTCCCCCTATTCCTA | 1865 | 932 | 85.615 | 45.8531 | -0.9008443 |
| novel_mir_34 | TTGAGCCGCGCCAATATCACT | 79 | 44 | 3.6266 | 2.1647 | -0.7444505 |
| novel_mir_44 | GCAGCGTCCTCAAGATTCACA | 107 | 61 | 4.912 | 3.0011 | -0.7108192 |
| novel_mir_39 | TTGTCGCAGGAGCGGTGGCACC | 2527 | 1457 | 116.005 | 71.6824 | -0.6944949 |
| novel_mir_43 | AGAAGAACGAGAGAAAGACGA | 166 | 98 | 7.6204 | 4.8215 | -0.6603847 |
| novel_mir_252 | TTGAGCCGCGTCAATATCTCC | 93 | 56 | 4.2693 | 2.7551 | -0.6318949 |
| novel_mir_58 | TGTTGGAACGGCTCAATCAAA | 3104 | 1992 | 142.493 | 98.0036 | -0.5399814 |
| novel_mir_99 | TTCCACCAAAGCATTCATTTCC | 2845 | 1921 | 130.603 | 94.5105 | -0.4666415 |
| novel_mir_433 | TCATGCGATCCCTTCGGAATT | 31 | 22 | 1.4231 | 1.0824 | -0.3948033 |
| novel_mir_132 | GTGACAGAAGATAGAGAGCGC | 43999 | 31274 | 2019.83 | 1538.6374 | -0.3925772 |
| novel_mir_280 | GGAATGGCGGCTGGTTCAAAG | 53 | 38 | 2.433 | 1.8695 | -0.3800839 |
| novel_mir_61 | CGCTATCCATCCTGAGTTTCA | 696 | 501 | 31.9507 | 24.6485 | -0.3743477 |
| novel_mir_368 | TTCCACAGCTTTCTTGAACTG | 2937 | 2143 | 134.826 | 105.4326 | -0.354782 |
| novel_mir_506 | TTGGCTGCGGCGGCGTCAACT | 30 | 22 | 1.3772 | 1.0824 | -0.3475043 |
| novel_mir_41 | GTAGATGATGAGTATGCACTG | 116 | 86 | 5.3251 | 4.2311 | -0.3317758 |
| novel_mir_142 | AGCAAGCATCCTGGGCTAAT | 436 | 326 | 20.0151 | 16.0387 | -0.3195316 |
| novel_mir_369 | AAGCTGTGGGAGAACATGGCA | 506 | 382 | 23.2285 | 18.7939 | -0.3056315 |
| novel_mir_499 | TTAGGGGGCATTTATACATAT | 1021 | 791 | 46.8702 | 38.9161 | -0.2683038 |
| novel_mir_375 | TTGTCGCCGGAGAGATAGCAC | 301 | 240 | 13.8178 | 11.8077 | -0.2268 |
| novel_mir_290 | CAAGAAACAAATAAGGAGGAGGT | 63 | 51 | 2.8921 | 2.5091 | -0.2049475 |
| novel_mir_25 | TGGCGCAGCTGTCCTAAACGG | 86 | 70 | 3.9479 | 3.4439 | -0.1970422 |
| novel_mir_479 | TTTGTTGAAGTTGCATGGAGA | 27 | 22 | 1.2395 | 1.0824 | -0.1955245 |
| novel_mir_117 | AGATCATCTGGCAGTTTCACC | 382 | 324 | 17.5362 | 15.9404 | -0.1376483 |
| novel_mir_222 | TGGGTTTGCATGCGCTTGAA | 34 | 29 | 1.5608 | 1.4268 | -0.1295026 |
| novel_mir_162 | TAATCGTGGGAGACGAAGCTG | 2489 | 2127 | 114.26 | 104.6454 | -0.1268166 |
| novel_mir_176 | TCTCATCGGTTTGGATGGCATT | 97 | 83 | 4.4529 | 4.0835 | -0.124939 |
| novel_mir_456 | TATGATTTTTGGATTTGGTATT | 31 | 27 | 1.4231 | 1.3284 | -0.0993474 |
| novel_mir_27 | AGATCATGCGGCAGTTTCACC | 1132 | 993 | 51.9658 | 48.8542 | -0.0890799 |
| novel_mir_37 | TAGCTTACGCCACACACAGCA | 119 | 107 | 5.4628 | 5.2643 | -0.0533989 |
| novel_mir_314 | TCCGACAATGTGGTAAACGTGTT | 106 | 96 | 4.8661 | 4.7231 | -0.0430319 |
| novel_mir_10 | TAATATAGGAATAAATTGGACA | 398 | 362 | 18.2707 | 17.8099 | -0.0368525 |
| novel_mir_317 | TTATTGGCTTTAGAAACAGGTT | 177 | 162 | 8.1254 | 7.9702 | -0.0278229 |
| novel_mir_445 | TTCCACAGCTTTCTTGAACTT | 514 | 474 | 23.5958 | 23.3201 | -0.0169561 |

* and ** indicate a significant difference at P < 0.05 and P < 0.01, respectively.
