# Supplementary material for: MicroRNA-mediated responses to long-term magnesium-deficiency in Citrus sinensis roots revealed by Illumina sequencing
Source: BMC Genomics. 2017 Aug 24;18:657. doi: 10.1186/s12864-017-3999-5 (PMC5571589; doi:10.1186/s12864-017-3999-5)
Supplement: Supplementary file 5 — List of novel miRNAs in C. sinensis roots after removing these miRNAs with normalized read-count less than 10 TPM in the two miRNA libraries constructed from Mg-sufficient and -deficient roots. (DOC 160 kb) [file 12864_2017_3999_MOESM5_ESM.doc]

**Additional file 5** List of novel miRNAs in *C. sinensis* roots after removing these miRNAs with normalized read-count less than 10 TPM in the two miRNA libraries constructed from Mg-sufficient and -deficient roots.

| miRNA | Sequence | | Expressed | | Normalized read count | | Fold change |
| --- | --- | --- | --- | --- | --- | --- | --- |
|  |  |  | Control | Mg-  deficiency | Control | Mg-  deficiency |  |
| **Up-regulated miRNAs** | | |  |  |  |  |  |
| novel_mir_585 | CGTGTCGTGGTGTAGTTGGT | | 0 | 62192 | 0.01 | 3059.76 | 18.22305897** |
| novel_mir_586 | AAGACTGTAGTGAACATG | | 0 | 5165 | 0.01 | 254.1108 | 14.63317007** |
| novel_mir_429 | TTGGATTGGGTAGAGTATTCGG | | 0 | 1120 | 0.01 | 55.1024 | 12.42789944** |
| novel_mir_468 | ATTGGGGGTAGATTGAGGTTT | | 0 | 956 | 0.01 | 47.0339 | 12.19948525** |
| novel_mir_470 | TGGGTGGCTTCTCGGACTTAC | | 0 | 867 | 0.01 | 42.6552 | 12.05850591** |
| novel_mir_587 | CGGAAGGGCCGCGGCGGC | | 0 | 767 | 0.01 | 37.7353 | 11.88169903** |
| novel_mir_504 | TGTGAGATGATTGTAAGTTAC | | 0 | 523 | 0.01 | 25.7309 | 11.3292862** |
| novel_mir_397 | CATGGGTGTTAATTGGTTCAAC | | 0 | 322 | 0.01 | 15.842 | 10.62953877** |
| novel_mir_497 | TGGCACTCTTCGGACCAATGC | | 0 | 305 | 0.01 | 15.0056 | 10.55128529** |
| novel_mir_588 | CTTGTAACTGTAGTAAGGTA | | 0 | 282 | 0.01 | 13.874 | 10.43816807** |
| novel_mir_589 | ACTAGTTAGATGGACCTAC | | 0 | 269 | 0.01 | 13.2344 | 10.37007707** |
| novel_mir_590 | TCGCGACCCAATGTGATTTTCGGA | | 0 | 237 | 0.01 | 11.6601 | 10.18736445** |
| novel_mir_406 | TGGTCGTGTACTTGGACGACAT | | 0 | 228 | 0.01 | 11.2173 | 10.13150975** |
| novel_mir_591 | ATGTAGAATCAAGGTAAA | | 1 | 273 | 0.046 | 13.4312 | 8.19287834** |
| novel_mir_592 | ATTATTGATTGTTAGGAT | | 2 | 415 | 0.092 | 20.4174 | 7.79708929** |
| novel_mir_593 | TGATTGATAGGGACAGTTGG | | 6 | 868 | 0.275 | 42.7044 | 7.27671235** |
| novel_mir_594 | TAGAGAGAGAGAGAGAGCGAGAG | | 7 | 889 | 0.321 | 43.7376 | 7.08880927** |
| novel_mir_595 | TTTTTTGGATCTGGATATA | | 2 | 238 | 0.092 | 11.7093 | 6.99494496** |
| novel_mir_596 | TGATTGGGAAGAAGACGACGA | | 17 | 688 | 0.78 | 33.8486 | 5.43873858** |
| novel_mir_597 | TAACTAATCGTGACGGTGACGGTGA | | 8 | 284 | 0.367 | 13.9724 | 5.24986998** |
| novel_mir_598 | ATGTTGTAGGAATGGAGGTAGGTA | | 66 | 1946 | 3.03 | 95.7405 | 4.98183487** |
| novel_mir_599 | TAGTGGGAGATTGTTGGGAAAAT | | 8 | 232 | 0.367 | 11.4141 | 4.95810724** |
| novel_mir_600 | AGCAGATACGGATCTTAAT | | 23 | 530 | 1.056 | 26.0753 | 4.62627538** |
| novel_mir_601 | TGGGGTGGGGATGGGGAAAGCATT | | 51 | 734 | 2.341 | 36.1118 | 3.94715024** |
| novel_mir_602 | TGAGAAAGGAGAGATGGTGCA | | 1662 | 16002 | 76.3 | 787.2762 | 3.36719054** |
| novel_mir_603 | ATGAGATGATGATGGATA | | 41 | 391 | 1.882 | 19.2367 | 3.35336949** |
| novel_mir_604 | TGAGAGCTTAGATCAGAAGATGAT | | 62 | 557 | 2.846 | 27.4036 | 3.26725648** |
| novel_mir_605 | TTCATGGAGAACTTGAAGT | | 76 | 447 | 3.489 | 21.9918 | 2.65612154** |
| novel_mir_426 | TTTCTCTTATCGTTATCTGTG | | 4689 | 21527 | 215.3 | 1059.0985 | 2.29872482** |
| novel_mir_606 | TGATAGTGACATAGATGATGGATG | | 505 | 1746 | 23.18 | 85.9008 | 1.88962919** |
| **Down-regulated miRNAs** | |  |  |  |  |  |  |
| novel_mir_607 | TTCTCTCAAGTAATTCTGACGGA | | 4919 | 0 | 225.8 | 0.01 | -14.46283864** |
| novel_mir_98 | TCACTACTTTCAATCTCGGTC | | 1154 | 0 | 52.98 | 0.01 | -12.37111497** |
| novel_mir_608 | GAGTGAAAGTGGGAGTAGGTTGTT | | 999 | 0 | 45.86 | 0.01 | -12.16303023** |
| novel_mir_609 | GGATGATCGAAAGTAAAAG | | 930 | 0 | 42.69 | 0.01 | -12.05977358** |
| novel_mir_610 | GGGGAGGGGACAAGGATC | | 396 | 0 | 18.18 | 0.01 | -10.82804129** |
| novel_mir_611 | TGGATAGTAGAATAATGAAGGAGA | | 1647 | 3 | 75.61 | 0.1476 | -9.00069089** |
| novel_mir_612 | GAGGAAGGAGAGATGGAGCAG | | 13674 | 65 | 627.7 | 3.1979 | -7.6168546** |
| novel_mir_614 | TGAGACGAGATGGGATGAG | | 792 | 6 | 13.68 | 0.0984 | -7.11919419** |
| novel_mir_613 | TGAAATTTTGGAGGACTT | | 298 | 2 | 36.36 | 0.2952 | -6.9444244** |
| novel_mir_615 | ATGTGGAGAATGAAATTATGAAGA | | 225 | 2 | 10.33 | 0.0984 | -6.71381259** |
| novel_mir_616 | CAGAAAGAAGACGAGTAG | | 773 | 17 | 35.49 | 0.8364 | -5.40689276** |
| novel_mir_617 | ATTTCGATAGTACGAGATTGT | | 348 | 11 | 15.98 | 0.5412 | -4.88353737** |
| novel_mir_618 | TAACTTCAAGTGGAATTCAGCAAA | | 264 | 24 | 12.12 | 1.1808 | -3.35945794** |
| novel_mir_619 | TGGACGGGGTTGATGGGCG | | 720 | 84 | 33.05 | 4.1327 | -2.99959849** |
| novel_mir_620 | TGAACAACTGGAGAAGCAA | | 1495 | 265 | 68.63 | 13.0376 | -2.39615473** |
| **Equally expressed miRNAs** | |  |  |  |  |  |  |
| novel_mir_443 | GCAGCATCATCAAGATTCACA | | 1290 | 2366 | 59.22 | 116.4039 | 0.9750098 |
| novel_mir_128 | CTGGAGACAACTGTGGTACGG | | 410 | 684 | 18.82 | 33.6519 | 0.83830635 |
| novel_mir_80 | GGAAACCCTAGGGGGAGGTCG | | 1738 | 2836 | 79.78 | 139.5273 | 0.80635979 |
| novel_mir_87 | TGGTATGGGTGAGTAGGGAAG | | 24766 | 37340 | 1137 | 1837.0761 | 0.6922908 |
| novel_mir_344 | GATACTCATTTAGGCAAGACG | | 577 | 818 | 26.49 | 40.2445 | 0.60345816 |
| novel_mir_144 | TTCAACTTTGAAAACGTCATC | | 465 | 650 | 21.35 | 31.9791 | 0.58313655 |
| novel_mir_207 | TGAGGTTCTTGGGGAGAGTAG | | 213 | 297 | 9.778 | 14.612 | 0.57954235 |
| novel_mir_410 | CCGTTGAGGTAGGGCAGTTCGG | | 34496 | 46166 | 1584 | 2271.3031 | 0.5203314 |
| novel_mir_401 | CTGGATGCAACTGTGGTACGG | | 9198 | 12258 | 422.2 | 603.0766 | 0.51426126 |
| novel_mir_420 | CCGCAGGGGCGACATGAGATC | | 1816 | 2411 | 83.37 | 118.6179 | 0.50879764 |
| novel_mir_164 | TGCTAGCGGCAAACCATGACAC | | 178 | 222 | 8.171 | 10.9221 | 0.41861275 |
| novel_mir_184 | AGTAAGATTGTCGTCACACAT | | 671 | 813 | 30.8 | 39.9985 | 0.37687503 |
| novel_mir_20 | GGTCATGGGAGGATTGGCGAGA | | 2E+05 | 183477 | 7041 | 9026.8136 | 0.35842803 |
| novel_mir_359 | AATGGGTGCATGGGCAAGAGA | | 7642 | 8898 | 350.8 | 437.7692 | 0.31946043 |
| novel_mir_360 | AGGTCATCTTGCAGCTTCAAT | | 184 | 209 | 8.447 | 10.2825 | 0.28373135 |
| novel_mir_135 | GCAATGCTCTTGAAGGACTAC | | 28606 | 32185 | 1313 | 1583.4573 | 0.27000043 |
| novel_mir_458 | CGAGGCTTGAACTAGTGCGGT | | 2662 | 2988 | 122.2 | 147.0055 | 0.26659987 |
| novel_mir_3 | GGAATGTTGTCTGGCTCGAGG | | 4613 | 5152 | 211.8 | 253.4712 | 0.25935699 |
| novel_mir_172 | GCTGTAGAAAGGCCCCTCAAC | | 7012 | 7801 | 321.9 | 383.7984 | 0.25376301 |
| novel_mir_503 | GCTGTAGATAGGCCCTTCAAC | | 2071 | 2283 | 95.07 | 112.3204 | 0.24053212 |
| novel_mir_173 | TCAAGGAGCGCACGAACGGTT | | 2451 | 2689 | 112.5 | 132.2951 | 0.23362946 |
| novel_mir_418 | TGTTTATTTCTTTGGGCGGCTG | | 1214 | 1329 | 55.73 | 65.385 | 0.23050296 |
| novel_mir_472 | GGAATGTTGTTTGGCTCGAGGG | | 1238 | 1319 | 56.83 | 64.893 | 0.19136445 |
| novel_mir_134 | AAGTCATTAGAAGAACTGCCG | | 21004 | 22154 | 964.2 | 1089.946 | 0.17683287 |
| novel_mir_442 | TACTTGCTGTATCGGTCGACAA | | 447 | 459 | 20.52 | 22.5822 | 0.13814828 |
| novel_mir_233 | AGCAGGAAAGTGGCTGGTTGA | | 442 | 452 | 20.29 | 22.2378 | 0.13220965 |
| novel_mir_411 | TTTGAACTCCTCGAAGCCTGC | | 1120 | 1139 | 51.41 | 56.0372 | 0.12419836 |
| novel_mir_127 | AGGGACAAGCTAAAAGACCAA | | 11367 | 11188 | 521.8 | 550.4341 | 0.0770306 |
| novel_mir_379 | ACTCTCCCTCAAGGGCTTCGC | | 1307 | 639 | 60 | 31.4379 | -0.93244322 |
| novel_mir_19 | TTCCCTAGTCCCCCTATTCCTA | | 1865 | 932 | 85.62 | 45.8531 | -0.90084431 |
| novel_mir_39 | TTGTCGCAGGAGCGGTGGCACC | | 2527 | 1457 | 116 | 71.6824 | -0.6944949 |
| novel_mir_58 | TGTTGGAACGGCTCAATCAAA | | 3104 | 1992 | 142.5 | 98.0036 | -0.53998136 |
| novel_mir_99 | TTCCACCAAAGCATTCATTTCC | | 2845 | 1921 | 130.6 | 94.5105 | -0.46664151 |
| novel_mir_132 | GTGACAGAAGATAGAGAGCGC | | 43999 | 31274 | 2020 | 1538.6374 | -0.39257716 |
| novel_mir_61 | CGCTATCCATCCTGAGTTTCA | | 696 | 501 | 31.95 | 24.6485 | -0.37434769 |
| novel_mir_368 | TTCCACAGCTTTCTTGAACTG | | 2937 | 2143 | 134.8 | 105.4326 | -0.35478199 |
| novel_mir_142 | AGCAAGCATCCTGGGCTAAT | | 436 | 326 | 20.02 | 16.0387 | -0.31953161 |
| novel_mir_369 | AAGCTGTGGGAGAACATGGCA | | 506 | 382 | 23.23 | 18.7939 | -0.30563152 |
| novel_mir_499 | TTAGGGGGCATTTATACATAT | | 1021 | 791 | 46.87 | 38.9161 | -0.26830381 |
| novel_mir_375 | TTGTCGCCGGAGAGATAGCAC | | 301 | 240 | 13.82 | 11.8077 | -0.22679996 |
| novel_mir_117 | AGATCATCTGGCAGTTTCACC | | 382 | 324 | 17.54 | 15.9404 | -0.13764833 |
| novel_mir_162 | TAATCGTGGGAGACGAAGCTG | | 2489 | 2127 | 114.3 | 104.6454 | -0.12681659 |
| novel_mir_27 | AGATCATGCGGCAGTTTCACC | | 1132 | 993 | 51.97 | 48.8542 | -0.08907987 |
| novel_mir_10 | TAATATAGGAATAAATTGGACA | | 398 | 362 | 18.27 | 17.8099 | -0.03685249 |
| novel_mir_445 | TTCCACAGCTTTCTTGAACTT | | 514 | 474 | 23.6 | 23.3201 | -0.01695611 |

** indicates a significant difference at *P* < 0.01.
