# Supplementary material for: MicroRNA-mediated responses to long-term magnesium-deficiency in Citrus sinensis roots revealed by Illumina sequencing
Source: BMC Genomics. 2017 Aug 24;18:657. doi: 10.1186/s12864-017-3999-5 (PMC5571589; doi:10.1186/s12864-017-3999-5)
Supplement: Supplementary file 6 — List of Mg-deficiency-responsive novel miRNAs in C. sinensis roots. (DOC 105 kb) [file 12864_2017_3999_MOESM6_ESM.doc]

**Additional file 6** List of Mg-deficiency-responsive novel miRNAs in *C. sinensis* roots.

| miRNA | Sequence | Expressed | | Normalized read count | | Fold change |
| --- | --- | --- | --- | --- | --- | --- |
|  |  | Control | Mg-  deficiency | Control | Mg-  deficiency |
| **Up-regulated miRNAs** | |  |  |  |  |  |
| novel_mir_585 | CGTGTCGTGGTGTAGTTGGT | 0 | 62192 | 0.01 | 3059.76 | 18.22305897** |
| novel_mir_586 | AAGACTGTAGTGAACATG | 0 | 5165 | 0.01 | 254.1108 | 14.63317007** |
| novel_mir_429 | TTGGATTGGGTAGAGTATTCGG | 0 | 1120 | 0.01 | 55.1024 | 12.4278994** |
| novel_mir_468 | ATTGGGGGTAGATTGAGGTTT | 0 | 956 | 0.01 | 47.0339 | 12.1994853** |
| novel_mir_470 | TGGGTGGCTTCTCGGACTTAC | 0 | 867 | 0.01 | 42.6552 | 12.0585059** |
| novel_mir_587 | CGGAAGGGCCGCGGCGGC | 0 | 767 | 0.01 | 37.7353 | 11.88169903** |
| novel_mir_504 | TGTGAGATGATTGTAAGTTAC | 0 | 523 | 0.01 | 25.7309 | 11.3292862** |
| novel_mir_397 | CATGGGTGTTAATTGGTTCAAC | 0 | 322 | 0.01 | 15.842 | 10.6295388** |
| novel_mir_497 | TGGCACTCTTCGGACCAATGC | 0 | 305 | 0.01 | 15.0056 | 10.5512853** |
| novel_mir_588 | CTTGTAACTGTAGTAAGGTA | 0 | 282 | 0.01 | 13.874 | 10.43816807** |
| novel_mir_589 | ACTAGTTAGATGGACCTAC | 0 | 269 | 0.01 | 13.2344 | 10.37007707** |
| novel_mir_590 | TCGCGACCCAATGTGATTTTCGGA | 0 | 237 | 0.01 | 11.6601 | 10.18736445** |
| novel_mir_406 | TGGTCGTGTACTTGGACGACAT | 0 | 228 | 0.01 | 11.2173 | 10.1315098** |
| novel_mir_591 | ATGTAGAATCAAGGTAAA | 1 | 273 | 0.0459 | 13.4312 | 8.19287834** |
| novel_mir_592 | ATTATTGATTGTTAGGAT | 2 | 415 | 0.0918 | 20.4174 | 7.79708929** |
| novel_mir_593 | TGATTGATAGGGACAGTTGG | 6 | 868 | 0.2754 | 42.7044 | 7.27671235** |
| novel_mir_594 | TAGAGAGAGAGAGAGAGCGAGAG | 7 | 889 | 0.3213 | 43.7376 | 7.08880927** |
| novel_mir_595 | TTTTTTGGATCTGGATATA | 2 | 238 | 0.0918 | 11.7093 | 6.99494496** |
| novel_mir_596 | TGATTGGGAAGAAGACGACGA | 17 | 688 | 0.7804 | 33.8486 | 5.43873858** |
| novel_mir_597 | TAACTAATCGTGACGGTGACGGTGA | 8 | 284 | 0.3672 | 13.9724 | 5.24986998** |
| novel_mir_598 | ATGTTGTAGGAATGGAGGTAGGTA | 66 | 1946 | 3.0298 | 95.7405 | 4.98183487** |
| novel_mir_599 | TAGTGGGAGATTGTTGGGAAAAT | 8 | 232 | 0.3672 | 11.4141 | 4.95810724** |
| novel_mir_600 | AGCAGATACGGATCTTAAT | 23 | 530 | 1.0558 | 26.0753 | 4.62627538** |
| novel_mir_601 | TGGGGTGGGGATGGGGAAAGCATT | 51 | 734 | 2.3412 | 36.1118 | 3.94715024** |
| novel_mir_602 | TGAGAAAGGAGAGATGGTGCA | 1662 | 16002 | 76.296 | 787.2762 | 3.36719054** |
| novel_mir_603 | ATGAGATGATGATGGATA | 41 | 391 | 1.8822 | 19.2367 | 3.35336949** |
| novel_mir_604 | TGAGAGCTTAGATCAGAAGATGAT | 62 | 557 | 2.8462 | 27.4036 | 3.26725648** |
| novel_mir_605 | TTCATGGAGAACTTGAAGT | 76 | 447 | 3.4889 | 21.9918 | 2.65612154** |
| novel_mir_426 | TTTCTCTTATCGTTATCTGTG | 4689 | 21527 | 215.25 | 1059.0985 | 2.29872482** |
| novel_mir_606 | TGATAGTGACATAGATGATGGATG | 505 | 1746 | 23.1826 | 85.9008 | 1.88962919** |
| **Down-regulated miRNAs** | |  |  |  |  |  |
| novel_mir_607 | TTCTCTCAAGTAATTCTGACGGA | 4919 | 0 | 225.8124 | 0.01 | -14.46283864** |
| novel_mir_98 | TCACTACTTTCAATCTCGGTC | 1154 | 0 | 52.9757 | 0.01 | -12.37111497** |
| novel_mir_608 | GAGTGAAAGTGGGAGTAGGTTGTT | 999 | 0 | 45.8603 | 0.01 | -12.16303023** |
| novel_mir_609 | GGATGATCGAAAGTAAAAG | 930 | 0 | 42.6927 | 0.01 | -12.05977358** |
| novel_mir_610 | GGGGAGGGGACAAGGATC | 396 | 0 | 18.1788 | 0.01 | -10.82804129** |
| novel_mir_611 | TGGATAGTAGAATAATGAAGGAGA | 1647 | 3 | 75.6074 | 0.1476 | -9.00069089** |
| novel_mir_612 | GAGGAAGGAGAGATGGAGCAG | 13674 | 65 | 627.7209 | 3.1979 | -7.6168546** |
| novel_mir_614 | TGAGACGAGATGGGATGAG | 792 | 6 | 13.68 | 0.0984 | -7.11919419** |
| novel_mir_613 | TGAAATTTTGGAGGACTT | 298 | 2 | 36.3577 | 0.2952 | -6.9444244** |
| novel_mir_615 | ATGTGGAGAATGAAATTATGAAGA | 225 | 2 | 10.3289 | 0.0984 | -6.71381259** |
| novel_mir_616 | CAGAAAGAAGACGAGTAG | 773 | 17 | 35.4855 | 0.8364 | -5.40689276** |
| novel_mir_617 | ATTTCGATAGTACGAGATTGT | 348 | 11 | 15.9753 | 0.5412 | -4.88353737** |
| novel_mir_618 | TAACTTCAAGTGGAATTCAGCAAA | 264 | 24 | 12.1192 | 1.1808 | -3.35945794** |
| novel_mir_619 | TGGACGGGGTTGATGGGCG | 720 | 84 | 33.0524 | 4.1327 | -2.99959849** |
| novel_mir_620 | TGAACAACTGGAGAAGCAA | 1495 | 265 | 68.6297 | 13.0376 | -2.39615473** |

** indicates a significant difference at *P* < 0.01.
