# Supplementary material for: MicroRNA-mediated responses to long-term magnesium-deficiency in Citrus sinensis roots revealed by Illumina sequencing
Source: BMC Genomics. 2017 Aug 24;18:657. doi: 10.1186/s12864-017-3999-5 (PMC5571589; doi:10.1186/s12864-017-3999-5)
Supplement: Supplementary file 7 — List of target genes for parts of known miRNAs in C. sinensis roots. (DOC 262 kb) [file 12864_2017_3999_MOESM7_ESM.doc]

**Additional file 7** List of target genes for parts of known miRNAs in *C. sinensis* roots.

| miRNA | Assession | Homology | Target genes |
| --- | --- | --- | --- |
| miR1077 | orange1.1g004517m | AT4G02880.2 |  |
| miR1091 | orange1.1g014056m |  |  |
| miR6150 | orange1.1g016242m | AT2G05990.2 | NAD(P)-binding Rossmann-fold superfamily protein |
|  | orange1.1g021851m | AT1G18330.2 | Homeodomain-like superfamily protein |
|  | orange1.1g021861m | AT1G18330.2 | Homeodomain-like superfamily protein |
|  | orange1.1g021875m | AT1G18330.2 | Homeodomain-like superfamily protein |
|  | orange1.1g022044m | AT1G18330.2 | Homeodomain-like superfamily protein |
|  | orange1.1g024702m | AT1G18330.2 | Homeodomain-like superfamily protein |
|  | orange1.1g009434m | AT5G62810.1 | Peroxin 14 |
|  | orange1.1g009455m | AT5G62810.1 | Peroxin 14 |
|  | orange1.1g009573m | AT5G62810.1 | Peroxin 14 |
|  | orange1.1g018459m | AT3G28715.1 | ATPase, V0/A0 complex, subunit C/D |
|  | orange1.1g018709m | AT3G28715.1 | ATPase, V0/A0 complex, subunit C/D |
|  | orange1.1g018928m | AT3G28715.1 | ATPase, V0/A0 complex, subunit C/D |
|  | orange1.1g024409m | AT3G28715.1 | ATPase, V0/A0 complex, subunit C/D |
|  | orange1.1g024398m | AT3G28715.1 | ATPase, V0/A0 complex, subunit C/D |
| miR158 | orange1.1g022993m | AT5G62740.1 | SPFH/Band 7/PHB domain-containing membrane-associated protein family |
|  | orange1.1g023219m | AT1G69840.6 | SPFH/Band 7/PHB domain-containing membrane-associated protein family |
|  | orange1.1g023253m | AT1G69840.7 | SPFH/Band 7/PHB domain-containing membrane-associated protein family |
|  | orange1.1g023247m | AT1G69840.5 | SPFH/Band 7/PHB domain-containing membrane-associated protein family |
|  | orange1.1g023256m | AT1G69840.3 | SPFH/Band 7/PHB domain-containing membrane-associated protein family |
|  | orange1.1g023237m | AT1G69840.6 | SPFH/Band 7/PHB domain-containing membrane-associated protein family |
|  | orange1.1g023977m | AT5G62740.1 | SPFH/Band 7/PHB domain-containing membrane-associated protein family |
|  | orange1.1g024195m | AT1G69840.3 | SPFH/Band 7/PHB domain-containing membrane-associated protein family |
|  | orange1.1g026063m | AT5G62740.1 | SPFH/Band 7/PHB domain-containing membrane-associated protein family |
|  | orange1.1g029372m | AT1G69840.1 | SPFH/Band 7/PHB domain-containing membrane-associated protein family |
| miR2119 | orange1.1g001751m | AT1G07810.1 | ER-type Ca^2+^-ATPase 1 |
|  | orange1.1g003468m | AT1G07810.1 | ER-type Ca^2+^-ATPase 1 |
|  | orange1.1g037409m | AT1G12700.1 | ATP binding;nucleic acid binding;helicases |
|  | orange1.1g011643m | AT1G77360.1 | Tetratricopeptide repeat (TPR)-like superfamily protein |
|  | orange1.1g011652m | AT1G77360.1 | Tetratricopeptide repeat (TPR)-like superfamily protein |
|  | orange1.1g011648m | AT1G77360.1 | Tetratricopeptide repeat (TPR)-like superfamily protein |
|  | orange1.1g000480m | AT3G26850.2 | Histone-lysine N-methyltransferases |
|  | orange1.1g000526m | AT3G26850.2 | Histone-lysine N-methyltransferases |
|  | orange1.1g000528m | AT3G26850.1 | Histone-lysine N-methyltransferases |
|  | orange1.1g000534m | AT3G26850.1 | Histone-lysine N-methyltransferases |
|  | orange1.1g000532m | AT3G26850.2 | Histone-lysine N-methyltransferases |
|  | orange1.1g000531m | AT3G26850.1 | Histone-lysine N-methyltransferases |
|  | orange1.1g000536m | AT3G26850.1 | Histone-lysine N-methyltransferases |
|  | orange1.1g000579m | AT3G26850.1 | Histone-lysine N-methyltransferases |
|  | orange1.1g001647m | AT3G26850.1 | Histone-lysine N-methyltransferases |
| miR2919 | orange1.1g002089m | AT3G14940.1 | Phosphoenolpyruvate carboxylase 3 |
| miR3437 | orange1.1g040557m | AT1G56140.1 | Leucine-rich repeat transmembrane protein kinase |
|  | orange1.1g012537m | AT2G37035.1 |  |
| miR414 | orange1.1g004767m | AT1G17980.1 | Poly(A) polymerase 1 |
|  | orange1.1g005049m | AT1G17980.1 | Poly(A) polymerase 1 |
|  | orange1.1g005391m | AT1G17980.1 | Poly(A) polymerase 1 |
|  | orange1.1g005445m | AT1G17980.1 | Poly(A) polymerase 1 |
|  | orange1.1g006232m | AT1G17980.1 | Poly(A) polymerase 1 |
| miR418 | orange1.1g003146m | AT1G20780.1 | Senescence-associated E3 ubiquitin ligase 1 |
| miR5176 | orange1.1g005789m | AT4G09140.1 | MUTL-homologue 1 |
|  | orange1.1g008397m | AT4G09140.1 | MUTL-homologue 1 |
|  | orange1.1g010846m | AT4G09140.1 | MUTL-homologue 1 |
|  | orange1.1g012406m | AT4G09140.1 | MUTL-homologue 1 |
| miR5635 | orange1.1g013841m | AT4G12730.1 | FASCICLIN-like arabinogalactan 2 |
|  | orange1.1g043249m | AT1G12210.1 | RPS5-like 1 |
| miR5830 | orange1.1g010316m | AT3G05990.1 | Leucine-rich repeat (LRR) family protein |
| miR6278 | orange1.1g005896m | AT3G14470.1 | NB-ARC domain-containing disease resistance protein |
|  | orange1.1g030696m | AT5G17840.1 | DnaJ/Hsp40 cysteine-rich domain superfamily protein |
| miR6427 | orange1.1g035636m | AT5G26250.1 | Major facilitator superfamily protein |
| miR7121 | orange1.1g005267m | AT1G71400.1 | Receptor like protein 12 |
|  | orange1.1g005542m | AT1G71400.1 | Receptor like protein 12 |
|  | orange1.1g008628m | AT1G71400.1 | Receptor like protein 12 |
|  | orange1.1g017310m | AT3G50910.1 |  |
|  | orange1.1g019263m | AT3G50910.1 |  |
|  | orange1.1g012980m | AT5G53390.1 | O-acyltransferase (WSD1-like) family protein |
|  | orange1.1g013532m | AT5G53390.1 | O-acyltransferase (WSD1-like) family protein |
|  | orange1.1g014554m | AT5G53390.1 | O-acyltransferase (WSD1-like) family protein |
|  | orange1.1g021833m | AT5G53390.1 | O-acyltransferase (WSD1-like) family protein |
|  | orange1.1g002167m | AT5G27060.1 | receptor like protein 53 |
|  | orange1.1g027358m | AT5G03080.1 | Phosphatidic acid phosphatase (PAP2) family protein |
|  | orange1.1g027361m | AT5G03080.1 | Phosphatidic acid phosphatase (PAP2) family protein |
|  | orange1.1g027353m | AT5G03080.1 | Phosphatidic acid phosphatase (PAP2) family protein |
| miR7497 | orange1.1g036915m | AT3G19184.1 | AP2/B3-like transcriptional factor family protein |
| miR7508 | orange1.1g025796m | AT1G67080.1 | Abscisic acid (aba)-deficient 4 |
| miR7821 | orange1.1g014348m | AT1G24160.1 |  |
|  | orange1.1g014335m | AT1G24160.1 |  |
|  | orange1.1g010745m | AT1G29760.1 | Putative adipose-regulatory protein (Seipin) |
| miR8130 | orange1.1g034513m |  |  |
| miR6426 | orange1.1g030569m | AT4G27390.1 |  |
| miR1847 | orange1.1g026316m | AT5G35530.1 | Ribosomal protein S3 family protein |
|  | orange1.1g026835m | AT5G35530.1 | Ribosomal protein S3 family protein |
|  | orange1.1g029201m | AT2G31610.1 | Ribosomal protein S3 family protein |
| miR6028 | orange1.1g005923m | AT2G33580.1 | Protein kinase superfamily protein |
|  | orange1.1g034040m | AT5G42990.1 | Ubiquitin-conjugating enzyme 18 |
|  | orange1.1g021729m | AT4G29100.1 | Basic helix-loop-helix (bHLH) DNA-binding superfamily protein |
|  | orange1.1g026539m | AT1G79020.1 | Enhancer of polycomb-like transcription factor protein |
|  | orange1.1g045123m | AT4G35800.1 | RNA polymerase II large subunit |
|  | orange1.1g003175m | AT4G14700.1 | Origin recognition complex 1 |
|  | orange1.1g006076m | AT3G46790.1 | Tetratricopeptide repeat (TPR)-like superfamily protein |
|  | orange1.1g029970m | AT3G49940.1 | LOB domain-containing protein 38 |
|  | orange1.1g028357m | AT2G45850.2 | AT-hook motif nuclear-localized protein 9 |
|  | orange1.1g003199m | AT5G49660.1 | Leucine-rich repeat transmembrane protein kinase family protein |
| miR8030 | orange1.1g034513m |  |  |
| miR4413 | orange1.1g031376m | AT5G02020.1 |  |
|  | orange1.1g031426m | AT5G02020.1 |  |
|  | orange1.1g031413m | AT5G02020.1 |  |
|  | orange1.1g031435m | AT5G02020.1 |  |
|  | orange1.1g031758m | AT5G02020.1 |  |
|  | orange1.1g031751m | AT5G02020.1 |  |
|  | orange1.1g031762m | AT5G02020.1 |  |
|  | orange1.1g032212m | AT5G02020.2 |  |
|  | orange1.1g032186m | AT5G02020.2 |  |
|  | orange1.1g034115m | AT5G02020.1 |  |
| miR6190 | orange1.1g029300m | AT5G64200.1 | Ortholog of human splicing factor SC35 |
|  | orange1.1g029316m | AT5G64200.2 | Ortholog of human splicing factor SC35 |
|  | orange1.1g017284m | AT5G34850.1 | Purple acid phosphatase 26 |
|  | orange1.1g002842m | AT4G01810.1 | Sec23/Sec24 protein transport family protein |
|  | orange1.1g002840m | AT4G01810.1 | Sec23/Sec24 protein transport family protein |
|  | orange1.1g002851m | AT4G01810.1 | Sec23/Sec24 protein transport family protein |
|  | orange1.1g002989m | AT4G01810.1 | Sec23/Sec24 protein transport family protein |
|  | orange1.1g002987m | AT4G01810.1 | Sec23/Sec24 protein transport family protein |
|  | orange1.1g002992m | AT4G01810.1 | Sec23/Sec24 protein transport family protein |
| miR6446 | orange1.1g039034m |  |  |
|  | orange1.1g016909m | AT5G09300.1 | Thiamin diphosphate-binding fold (THDP-binding) superfamily protein |
|  | orange1.1g016926m | AT5G09300.1 | Thiamin diphosphate-binding fold (THDP-binding) superfamily protein |
|  | orange1.1g016903m | AT5G09300.1 | Thiamin diphosphate-binding fold (THDP-binding) superfamily protein |
|  | orange1.1g019322m | AT5G09300.1 | Thiamin diphosphate-binding fold (THDP-binding) superfamily protein |
|  | orange1.1g019498m | AT5G09300.1 | Thiamin diphosphate-binding fold (THDP-binding) superfamily protein |
|  | orange1.1g022717m | AT5G09300.1 | Thiamin diphosphate-binding fold (THDP-binding) superfamily protein |
|  | orange1.1g023827m | AT5G09300.1 | Thiamin diphosphate-binding fold (THDP-binding) superfamily protein |
|  | orange1.1g026778m | AT5G09300.2 | Thiamin diphosphate-binding fold (THDP-binding) superfamily protein |
|  | orange1.1g029082m | AT5G09300.1 | Thiamin diphosphate-binding fold (THDP-binding) superfamily protein |
|  | orange1.1g001557m | AT5G20280.1 | Sucrose phosphate synthase 1F |
|  | orange1.1g002665m | AT5G20280.1 | Sucrose phosphate synthase 1F |
| miR6485 | orange1.1g030767m | AT2G01590.1 | Chlororespiratory reduction 3 |
|  | orange1.1g000763m | AT3G07160.1 | Glucan synthase-like 10 |
|  | orange1.1g023138m | AT4G32030.1 |  |
|  | orange1.1g025058m | AT4G32030.1 |  |
|  | orange1.1g001969m | AT5G20730.2 | Transcriptional factor B3 family protein / auxin-responsive factor AUX/IAA-related |
|  | orange1.1g002397m | AT5G20730.2 | Transcriptional factor B3 family protein / auxin-responsive factor AUX/IAA-related |
|  | orange1.1g002396m | AT5G20730.2 | Transcriptional factor B3 family protein / auxin-responsive factor AUX/IAA-related |
|  | orange1.1g003107m | AT5G20730.2 | Transcriptional factor B3 family protein / auxin-responsive factor AUX/IAA-related |
|  | orange1.1g003369m | AT5G20730.2 | Transcriptional factor B3 family protein / auxin-responsive factor AUX/IAA-related |
|  | orange1.1g011274m | AT3G22810.1 | Plant protein of unknown function (DUF828) with plant pleckstrin homology-like region |
|  | orange1.1g011337m | AT3G22810.1 | Plant protein of unknown function (DUF828) with plant pleckstrin homology-like region |
|  | orange1.1g015144m | AT4G14740.1 | Plant protein of unknown function (DUF828) with plant pleckstrin homology-like region |
|  | orange1.1g015365m | AT4G14740.1 | Plant protein of unknown function (DUF828) with plant pleckstrin homology-like region |
|  | orange1.1g015388m | AT4G14740.1 | Plant protein of unknown function (DUF828) with plant pleckstrin homology-like region |
|  | orange1.1g015357m | AT4G14740.1 | Plant protein of unknown function (DUF828) with plant pleckstrin homology-like region |
|  | orange1.1g031218m | AT1G07400.1 | HSP20-like chaperones superfamily protein |
|  | orange1.1g009779m | AT1G08960.1 | cation exchanger 11 |
|  | orange1.1g029454m | AT5G51160.1 | Ankyrin repeat family protein |
|  | orange1.1g034979m |  |  |
|  | orange1.1g013633m | AT1G28560.1 | snRNA activating complex family protein |
|  | orange1.1g017698m | AT1G28560.1 | snRNA activating complex family protein |
|  | orange1.1g042988m | AT5G62850.1 | Nodulin MtN3 family protein |
|  | orange1.1g007868m | AT1G72650.2 | Myb family transcription factor TRFL6 |
|  | orange1.1g046667m | AT2G38940.1 | Phosphate transporter 1;4 |
|  | orange1.1g001289m | AT1G14610.1 | Valyl-tRNA synthetase / valine--tRNA ligase (VALRS) |
|  | orange1.1g001303m | AT1G14610.1 | Valyl-tRNA synthetase / valine--tRNA ligase (VALRS) |
|  | orange1.1g001708m | AT1G14610.1 | Valyl-tRNA synthetase / valine--tRNA ligase (VALRS) |
|  | orange1.1g001757m | AT1G14610.1 | Valyl-tRNA synthetase / valine--tRNA ligase (VALRS) |
|  | orange1.1g024117m | AT2G47920.1 | Kinase interacting (KIP1-like) family protein |
|  | orange1.1g036588m | AT4G20140.1 | Leucine-rich repeat transmembrane protein kinase |
|  | orange1.1g023040m | AT2G47360.1 |  |
|  | orange1.1g003591m | AT5G05680.1 | Nuclear pore complex protein NUP88 |
|  | orange1.1g033428m | AT5G40240.2 | Nodulin MtN21 /EamA-like transporter family protein |
| miR1044 | orange1.1g001378m | AT1G10170.1 | NF-X-like 1 |
|  | orange1.1g001377m | AT1G10170.1 | NF-X-like 1 |
|  | orange1.1g001376m | AT1G10170.1 | NF-X-like 1 |
|  | orange1.1g047796m | AT2G38380.1 | Peroxidase superfamily protein |
|  | orange1.1g042193m | AT5G03340.1 | ATPase, AAA-type, CDC48 protein |
|  | orange1.1g019546m | AT2G40340.1 | Integrase-type DNA-binding superfamily protein |
| miR5762 | orange1.1g010779m | AT5G06900.1 | Cytochrome P450, family 93, subfamily D, polypeptide 1 |
|  | orange1.1g033819m | AT4G12970.1 | Stomagen |
| miR5198 | orange1.1g002063m | AT1G72180.1 | Leucine-rich receptor-like protein kinase family protein |
|  | orange1.1g002061m | AT1G72180.1 | Leucine-rich receptor-like protein kinase family protein |
|  | orange1.1g003065m | AT1G72180.1 | Leucine-rich receptor-like protein kinase family protein |
|  | orange1.1g003067m | AT1G72180.1 | Leucine-rich receptor-like protein kinase family protein |
|  | orange1.1g003066m | AT1G72180.1 | Leucine-rich receptor-like protein kinase family protein |
| miR394 | orange1.1g000114m | AT1G20960.1 | U5 small nuclear ribonucleoprotein helicase, putative |
|  | orange1.1g000129m | AT1G20960.2 | U5 small nuclear ribonucleoprotein helicase, putative |
|  | orange1.1g000324m | AT1G20960.1 | U5 small nuclear ribonucleoprotein helicase, putative |
|  | orange1.1g000756m | AT1G20960.1 | U5 small nuclear ribonucleoprotein helicase, putative |
| miR5641 | orange1.1g043526m | AT4G29990.1 | Leucine-rich repeat transmembrane protein kinase protein |
| miR3637 | orange1.1g043678m | AT4G03460.1 | Ankyrin repeat family protein |
| miR5029 | orange1.1g012168m | AT5G53450.1 | OBP3-responsive gene 1 |
|  | orange1.1g026587m | AT4G31300.3 | N-terminal nucleophile aminohydrolases (Ntn hydrolases) superfamily protein |
|  | orange1.1g029964m | AT4G31300.3 | N-terminal nucleophile aminohydrolases (Ntn hydrolases) superfamily protein |
|  | orange1.1g030788m | AT4G31300.3 | N-terminal nucleophile aminohydrolases (Ntn hydrolases) superfamily protein |
|  | orange1.1g014625m | AT3G23510.1 | Cyclopropane-fatty-acyl-phospholipid synthase |
|  | orange1.1g018123m | AT3G44160.1 | Outer membrane OMP85 family protein |
| miR5261 | orange1.1g018132m | AT3G56930.1 | DHHC-type zinc finger family protein |
|  | orange1.1g010695m | AT3G12640.1 | RNA binding (RRM/RBD/RNP motifs) family protein |
|  | orange1.1g011967m | AT3G12640.1 | RNA binding (RRM/RBD/RNP motifs) family protein |
|  | orange1.1g031636m | AT1G67620.1 | Lojap-related protein |
|  | orange1.1g033883m | AT1G67620.1 | Lojap-related protein |
|  | orange1.1g035219m |  |  |
|  | orange1.1g004959m | AT5G66850.1 | Mitogen-activated protein kinase kinase kinase 5 |
|  | orange1.1g043928m | AT2G36110.1 | Polynucleotidyl transferase, ribonuclease H-like superfamily protein |
|  | orange1.1g004713m | AT5G54260.1 | DNA repair and meiosis protein (Mre11) |
|  | orange1.1g004738m | AT5G54260.1 | DNA repair and meiosis protein (Mre11) |
|  | orange1.1g004751m | AT5G54260.1 | DNA repair and meiosis protein (Mre11) |
|  | orange1.1g006814m | AT5G54260.1 | DNA repair and meiosis protein (Mre11) |
|  | orange1.1g006878m | AT5G54260.1 | DNA repair and meiosis protein (Mre11) |
|  | orange1.1g013207m | AT5G54260.1 | DNA repair and meiosis protein (Mre11) |
|  | orange1.1g010785m | AT3G26020.2 | Protein phosphatase 2A regulatory B subunit family protein |
|  | orange1.1g015127m | AT3G26020.2 | Protein phosphatase 2A regulatory B subunit family protein |
|  | orange1.1g000012m | AT1G55860.2 | Ubiquitin-protein ligase 1 |
|  | orange1.1g000013m | AT1G55860.2 | Ubiquitin-protein ligase 1 |
|  | orange1.1g029528m | AT5G01520.1 | RING/U-box superfamily protein |
|  | orange1.1g029508m | AT1G22360.1 | UDP-glucosyl transferase 85A2 |
|  | orange1.1g037980m | AT2G36110.1 | Polynucleotidyl transferase, ribonuclease H-like superfamily protein |
| miR2665 | orange1.1g004491m | AT5G66420.2 |  |
|  | orange1.1g044889m | AT5G27650.1 | Tudor/PWWP/MBT superfamily protein |
|  | orange1.1g003139m | AT5G54380.1 | Protein kinase family protein |
|  | orange1.1g042323m | AT3G07080.1 | EamA-like transporter family |
|  | orange1.1g001349m | AT3G06880.2 | Transducin/WD40 repeat-like superfamily protein |
|  | orange1.1g002234m | AT3G06880.2 | Transducin/WD40 repeat-like superfamily protein |
| miR5290 | orange1.1g047996m |  |  |
| miR7708 | orange1.1g023136m | AT1G06890.1 | Nodulin MtN21 /EamA-like transporter family protein |
| miR3438 | orange1.1g000163m | AT1G55325.2 | RNA polymerase II transcription mediators |
| miR6289 | orange1.1g035310m |  |  |
| miR1151 | orange1.1g018149m | AT5G49610.1 | F-box family protein |
|  | orange1.1g018125m | AT5G49610.1 | F-box family protein |
|  | orange1.1g023739m | AT2G41870.1 | Remorin family protein |
|  | orange1.1g027436m | AT2G41870.1 | Remorin family protein |
|  | orange1.1g018817m | AT2G36690.1 | 2-oxoglutarate (2OG) and Fe(II)-dependent oxygenase superfamily protein |
|  | orange1.1g023033m | AT2G36690.1 | 2-oxoglutarate (2OG) and Fe(II)-dependent oxygenase superfamily protein |
|  | orange1.1g026453m | AT1G17020.1 | Senescence-related gene 1 |
|  | orange1.1g020233m | AT2G36690.1 | 2-oxoglutarate (2OG) and Fe(II)-dependent oxygenase superfamily protein |
|  | orange1.1g037473m | AT5G07480.1 | KAR-UP oxidoreductase 1 |
| miR6219 | orange1.1g010903m | AT5G15130.1 | WRKY DNA-binding protein 72 |
| miR780 | orange1.1g044623m | AT5G17230.2 | PHYTOENE SYNTHASE |
|  | orange1.1g030826m | AT2G26560.1 | Phospholipase A 2A |
|  | orange1.1g030813m | AT2G26560.1 | Phospholipase A 2A |
|  | orange1.1g004573m | AT4G27220.1 | NB-ARC domain-containing disease resistance protein |
| miR1222 | orange1.1g037429m | AT4G27220.1 | NB-ARC domain-containing disease resistance protein |
| miR160 | orange1.1g005482m | AT4G30080.1 | Auxin response factor 18 |
|  | orange1.1g004896m | AT2G28350.1 | Auxin response factor 10 |
|  | orange1.1g004898m | AT2G28350.1 | Auxin response factor 10 |
|  | orange1.1g005799m | AT2G28350.1 | Auxin response factor 10 |
|  | orange1.1g005075m | AT4G30080.1 | Auxin response factor 18 |
|  | orange1.1g005296m | AT4G30080.1 | Auxin response factor 18 |
|  | orange1.1g008078 | AT1G77850.1 | Auxin response factor 17 |
|  | orange1.1g008088m | AT1G77850.1 | Auxin response factor 17 |
| miR169 | orange1.1g007017m | AT3G20770.1 | Ethylene insensitive 3 family protein |
|  | orange1.1g037813m | AT1G72100.1 | Late embryogenesis abundant domain-containing protein / LEA domain-containing protein |
