# Supplementary material for: MicroRNA-mediated responses to long-term magnesium-deficiency in Citrus sinensis roots revealed by Illumina sequencing
Source: BMC Genomics. 2017 Aug 24;18:657. doi: 10.1186/s12864-017-3999-5 (PMC5571589; doi:10.1186/s12864-017-3999-5)
Supplement: Supplementary file 8 — List of target genes for parts of novel miRNAs in C. sinensis roots. (DOCX 25 kb) [file 12864_2017_3999_MOESM8_ESM.docx]

**Additional file 8** List of target genes for parts of novel miRNAs in *C. sinensis* roots

| miRNA | Assession | Homology | Target genes |
| --- | --- | --- | --- |
| novel_mir_504 | orange1.1g031376m | AT5G02020.1 |  |
|  | orange1.1g032186m | AT5G02020.2 |  |
| novel_mir_497 | orange1.1g006973m | AT5G08020.1 | RPA70-kDa subunit B |
| novel_mir_592 | orange1.1g047229m | AT2G39210.1 | Major facilitator superfamily protein |
|  | orange1.1g041571m | AT2G35230.1 | VQ motif-containing protein |
| novel_mir_595 | orange1.1g002655m | AT5G63020.1 | Disease resistance protein (CC-NBS-LRR class) family |
|  | orange1.1g043249m | AT1G12210.1 | RPS5-like 1 |
| novel_mir_598 | orange1.1g037514m | AT2G34930.1 | disease resistance family protein / LRR family protein |
|  | orange1.1g038206m | AT1G59720.1 | Tetratricopeptide repeat (TPR)-like superfamily protein |
|  | orange1.1g030720m | AT5G08480.2 | VQ motif-containing protein |
|  | orange1.1g030743m | AT5G08480.2 | VQ motif-containing protein |
|  | orange1.1g040527m | AT2G26030.1 | F-box/RNI-like/FBD-like domains-containing protein |
| novel_mir_600 | orange1.1g010747m | AT4G10770.1 | Oligopeptide transporter 7 |
|  | orange1.1g014451m | AT4G10770.1 | Oligopeptide transporter 7 |
|  | orange1.1g014857m | AT4G10770.1 | Oligopeptide transporter 7 |
|  | orange1.1g000229m | AT1G55540.2 | Nuclear pore complex protein |
|  | orange1.1g000282m | AT1G55540.2 | Nuclear pore complex protein |
|  | orange1.1g000716m | AT1G55540.1 | Nuclear pore complex protein |
|  | orange1.1g000733m | AT1G55540.1 | Nuclear pore complex protein |
|  | orange1.1g000717m | AT4G02510.1 | translocon at the outer envelope membrane of chloroplasts 159 |
| novel_mir_603 | orange1.1g005082m | AT3G51950.2 | Zinc finger (CCCH-type) family protein / RNA recognition motif (RRM)-containing protein |
|  | orange1.1g005069m | AT3G51950.2 | Zinc finger (CCCH-type) family protein / RNA recognition motif (RRM)-containing protein |
|  | orange1.1g005083m | AT3G51950.1 | Zinc finger (CCCH-type) family protein / RNA recognition motif (RRM)-containing protein |
|  | orange1.1g005121m | AT3G51950.1 | Zinc finger (CCCH-type) family protein / RNA recognition motif (RRM)-containing protein |
|  | orange1.1g005337m | AT3G51950.2 | Zinc finger (CCCH-type) family protein / RNA recognition motif (RRM)-containing protein |
|  | orange1.1g005524m | AT3G51950.2 | Zinc finger (CCCH-type) family protein / RNA recognition motif (RRM)-containing protein |
|  | orange1.1g005533m | AT3G51950.1 | Zinc finger (CCCH-type) family protein / RNA recognition motif (RRM)-containing protein |
|  | orange1.1g006992m | AT3G51950.1 | Zinc finger (CCCH-type) family protein / RNA recognition motif (RRM)-containing protein |
|  | orange1.1g007955m | AT3G51950.1 | Zinc finger (CCCH-type) family protein / RNA recognition motif (RRM)-containing protein |
|  | orange1.1g040465m | AT5G17860.1 | Calcium exchanger 7 |
| novel_mir_605 | orange1.1g039493m | AT2G29140.1 | Pumilio 3 |
|  | orange1.1g001921m | AT3G45630.1 | RNA binding (RRM/RBD/RNP motifs) family protein |
|  | orange1.1g001930m | AT3G45630.1 | RNA binding (RRM/RBD/RNP motifs) family protein |
|  | orange1.1g001925m | AT3G45630.1 | RNA binding (RRM/RBD/RNP motifs) family protein |
|  | orange1.1g001933m | AT3G45630.1 | RNA binding (RRM/RBD/RNP motifs) family protein |
|  | orange1.1g001923m | AT3G45630.1 | RNA binding (RRM/RBD/RNP motifs) family protein |
|  | orange1.1g001934m | AT3G45630.1 | RNA binding (RRM/RBD/RNP motifs) family protein |
|  | orange1.1g001929m | AT3G45630.1 | RNA binding (RRM/RBD/RNP motifs) family protein |
|  | orange1.1g002482m | AT3G45630.1 | RNA binding (RRM/RBD/RNP motifs) family protein |
|  | orange1.1g003956m | AT3G45630.1 | RNA binding (RRM/RBD/RNP motifs) family protein |
| novel_mir_609 | orange1.1g041987m | AT1G60050.1 | Nodulin MtN21 /EamA-like transporter family protein |
| novel_mir_610 | orange1.1g043324m | AT1G16380.1 | Cation/hydrogen exchanger family protein |
|  | orange1.1g011754m | AT1G12330.1 |  |
|  | orange1.1g003749m | AT5G04810.1 | Pentatricopeptide (PPR) repeat-containing protein |
|  | orange1.1g004006m | AT5G04810.1 | Pentatricopeptide (PPR) repeat-containing protein |
|  | orange1.1g010223m | AT1G73240.1 |  |
|  | orange1.1g036474m | AT5G57090.1 | Auxin efflux carrier family protein |
|  | orange1.1g016003m | AT5G53750.1 | CBS domain-containing protein |
|  | orange1.1g003353m | AT1G79490.1 | Pentatricopeptide repeat (PPR) superfamily protein |
|  | orange1.1g008218m | AT3G16090.1 | RING/U-box superfamily protein |
|  | orange1.1g010385m | AT3G16090.1 | RING/U-box superfamily protein |
|  | orange1.1g015350m | AT3G16090.1 | RING/U-box superfamily protein |
|  | orange1.1g002510m | AT2G20050.1 | Protein serine/threonine phosphatases;protein kinases;catalytics;cAMP-dependent protein kinase regulators;ATP binding;protein serine/threonine phosphatases |
| novel_mir_612 | orange1.1g026986m | AT2G36895.1 |  |
|  | orange1.1g027133m | AT2G36895.1 |  |
|  | orange1.1g006213m | AT1G49890.1 | Family of unknown function (DUF566) |
|  | orange1.1g009652m | AT1G49890.1 | Family of unknown function (DUF566) |
|  | orange1.1g042284m | AT4G21990.1 | APS reductase 3 |
|  | orange1.1g005441m | AT1G48110.2 | Evolutionarily conserved C-terminal region 7 |
|  | orange1.1g005453m | AT1G48110.1 | Evolutionarily conserved C-terminal region 7 |
|  | orange1.1g005911m | AT1G48110.2 | Evolutionarily conserved C-terminal region 7 |
|  | orange1.1g009381m | AT1G48110.1 | Evolutionarily conserved C-terminal region 7 |
|  | orange1.1g006668m | AT1G15530.1 | Concanavalin A-like lectin protein kinase family protein |
|  | orange1.1g008238m | AT4G05200.1 | Cysteine-rich RLK (RECEPTOR-like protein kinase) 25 |
|  | orange1.1g013018m | AT4G05200.1 | Cysteine-rich RLK (RECEPTOR-like protein kinase) 25 |
|  | orange1.1g004813m | AT5G64320.1 | Pentatricopeptide repeat (PPR) superfamily protein |
| novel_mir_614 | orange1.1g003771m | AT1G75730.1 |  |
|  | orange1.1g003993m | AT1G75730.1 |  |
|  | orange1.1g025613m | AT4G36910.1 | Cystathionine beta-synthase (CBS) family protein |
|  | orange1.1g025984m | AT4G36910.1 | Cystathionine beta-synthase (CBS) family protein |
|  | orange1.1g026495m | AT4G36910.1 | Cystathionine beta-synthase (CBS) family protein |
|  | orange1.1g028550m | AT4G36910.1 | Cystathionine beta-synthase (CBS) family protein |
|  | orange1.1g028558m | AT4G36910.1 | Cystathionine beta-synthase (CBS) family protein |
|  | orange1.1g028828m | AT4G36910.1 | Cystathionine beta-synthase (CBS) family protein |
|  | orange1.1g028824m | AT4G36910.1 | Cystathionine beta-synthase (CBS) family protein |
|  | orange1.1g028787m | AT4G36910.1 | Cystathionine beta-synthase (CBS) family protein |
|  | orange1.1g028794m | AT4G36910.1 | Cystathionine beta-synthase (CBS) family protein |
|  | orange1.1g030127m | AT4G36910.1 | Cystathionine beta-synthase (CBS) family protein |
|  | orange1.1g030753m | AT4G36910.1 | Cystathionine beta-synthase (CBS) family protein |
|  | orange1.1g003508m | AT1G78580.1 | Trehalose-6-phosphate synthase |
| novel_mir_616 | orange1.1g033123m | AT4G25200.1 | Mitochondrion-localized small heat shock protein 23.6 |
|  | orange1.1g002891m | AT1G77800.2 | PHD finger family protein |
|  | orange1.1g015708m | AT1G07160.1 | Protein phosphatase 2C family protein |
|  | orange1.1g021587m | AT5G47740.1 | Adenine nucleotide alpha hydrolases-like superfamily protein |
|  | orange1.1g021843m | AT5G47740.1 | Adenine nucleotide alpha hydrolases-like superfamily protein |
|  | orange1.1g024855m | AT5G47740.1 | Adenine nucleotide alpha hydrolases-like superfamily protein |
|  | orange1.1g026046m | AT5G47740.1 | Adenine nucleotide alpha hydrolases-like superfamily protein |
|  | orange1.1g029098m | AT5G47740.1 | Adenine nucleotide alpha hydrolases-like superfamily protein |
|  | orange1.1g029072m | AT5G47740.1 | Adenine nucleotide alpha hydrolases-like superfamily protein |
|  | orange1.1g010594m | AT5G16890.1 | Exostosin family protein |
| novel_mir_619 | orange1.1g013840m | AT4G03110.1 | RNA-binding protein-defense related 1 |
|  | orange1.1g013926m | AT4G03110.1 | RNA-binding protein-defense related 1 |
|  | orange1.1g014217m | AT4G03110.1 | RNA-binding protein-defense related 1 |
|  | orange1.1g015716m | AT4G03110.2 | RNA-binding protein-defense related 1 |
|  | orange1.1g018554m | AT4G03110.1 | RNA-binding protein-defense related 1 |
|  | orange1.1g020372m | AT4G03110.1 | RNA-binding protein-defense related 1 |
| novel_mir_620 | orange1.1g023301m | AT1G80480.1 | plastid transcriptionally active 17 |
|  | orange1.1g012607m | AT2G24100.1 |  |
|  | orange1.1g012576m | AT2G24100.1 |  |
|  | orange1.1g012584m | AT2G24100.1 |  |
|  | orange1.1g012598m | AT2G24100.1 |  |
|  | orange1.1g012586m | AT2G24100.1 |  |
|  | orange1.1g012662m | AT2G24100.1 |  |
|  | orange1.1g014988m | AT2G24100.1 |  |
|  | orange1.1g017055m | AT2G24100.1 |  |
|  | orange1.1g017073m | AT2G24100.1 |  |
|  | orange1.1g017039m | AT2G24100.1 |  |
|  | orange1.1g019455m | AT2G24100.1 |  |
|  | orange1.1g022873m | AT2G24100.1 |  |
|  | orange1.1g022839m | AT2G24100.1 |  |
|  | orange1.1g014069m | AT5G01960.1 | RING/U-box superfamily protein |
|  | orange1.1g015126m | AT5G01960.1 | RING/U-box superfamily protein |
|  | orange1.1g019297m | AT5G01960.1 | RING/U-box superfamily protein |
|  | orange1.1g004928m | AT2G25930.1 | Hydroxyproline-rich glycoprotein family protein |
|  | orange1.1g013854m | AT2G25930.1 | Hydroxyproline-rich glycoprotein family protein |
|  | orange1.1g016509m | AT2G25930.1 | Hydroxyproline-rich glycoprotein family protein |
|  | orange1.1g017662m | AT2G25930.1 | Hydroxyproline-rich glycoprotein family protein |
|  | orange1.1g017850m | AT2G25930.1 | Hydroxyproline-rich glycoprotein family protein |
|  | orange1.1g017841m | AT2G25930.1 | Hydroxyproline-rich glycoprotein family protein |
|  | orange1.1g011625m | AT1G28050.1 | B-box type zinc finger protein with CCT domain |
|  | orange1.1g014102m | AT1G28050.1 | B-box type zinc finger protein with CCT domain |
|  | orange1.1g021343m | AT1G28050.1 | B-box type zinc finger protein with CCT domain |
|  | orange1.1g015465m | AT1G30580.1 | GTP binding |
|  | orange1.1g016139m | AT1G30580.1 | GTP binding |
|  | orange1.1g017295m | AT1G30580.1 | GTP binding |
|  | orange1.1g018809m | AT1G30580.1 | GTP binding |
|  | orange1.1g024748m | AT1G30580.1 | GTP binding |
|  | orange1.1g027700m | AT5G65430.1 | General regulatory factor 8 |
|  | orange1.1g029855m | AT5G10450.1 | G-box regulating factor 6 |
|  | orange1.1g029870m | AT5G10450.1 | G-box regulating factor 6 |
