# Supplementary material for: MicroRNA-mediated responses to long-term magnesium-deficiency in Citrus sinensis roots revealed by Illumina sequencing
Source: BMC Genomics. 2017 Aug 24;18:657. doi: 10.1186/s12864-017-3999-5 (PMC5571589; doi:10.1186/s12864-017-3999-5)
Supplement: Supplementary file 9 — Stem loop primer sequences for qRT-PCR analysis of miRNAs. (DOCX 17 kb) [file 12864_2017_3999_MOESM9_ESM.docx]

| **Additional file 9** Stem loop primer sequences for qRT-PCR analysis of miRNAs | | | |
| --- | --- | --- | --- |
| miRNA | Sequence | RT-primer (5'→3') | Forward primer (5'→3') |
| miR2919 | AATGGGAGGGGGGGGCAAGAA | GTCGTATCCAGTGCAGGGTCCGAGGTATTCGCACTGGATACGACTTCTTG | GAATTAATGGGAGGGGGGGGCA |
| miR414 | TCATCATCATCATCGTCGTCGTC | GTCGTATCCAGTGCAGGGTCCGAGGTATTCGCACTGGATACGACGACGAC | GACGGTCATCATCATCATCGTCGT |
| miR418 | TTTGTGATGATTGAAATGAGG | GTCGTATCCAGTGCAGGGTCCGAGGTATTCGCACTGGATACGACCCTCAT | GCCGGCTTTGTGATGATTGAAATG |
| miR7121 | TCCTCTTGGGATCGACACTCGT | GTCGTATCCAGTGCAGGGTCCGAGGTATTCGCACTGGATACGACACGAGT | GAGCTCCTCTTGGGATCGACA |
| miR1847 | TGGACTTTGCAGGTTGGGCAC | GTCGTATCCAGTGCAGGGTCCGAGGTATTCGCACTGGATACGACGTGCCC | AGCGTGGACTTTGCAGGTTGG |
| miR6028 | AGGAGATTAAGGACATTAA | GTCGTATCCAGTGCAGGGTCCGAGGTATTCGCACTGGATACGACTTAATG | ACGCGGAGGAGATTAAGGACA |
| miR6190 | CGAGAAAAGGAAAAGACAG | GTCGTATCCAGTGCAGGGTCCGAGGTATTCGCACTGGATACGACCTGTCT | AGCGCGCGAGAAAAGGAAAAG |
| miR6446 | TGTGGGTGCTTGATGATGGA | GTCGTATCCAGTGCAGGGTCCGAGGTATTCGCACTGGATACGACTCCATC | ACGTGTGGGTGCTTGATGATGG |
| miR6485 | AGAATGTAGAAGAGGTAA | GTCGTATCCAGTGCAGGGTCCGAGGTATTCGCACTGGATACGACTTACCT | GAGCGGAGAATGTAGAAGAGG |
| miR1044 | TTGTGGGCATATTTCTTTTA | GTCGTATCCAGTGCAGGGTCCGAGGTATTCGCACTGGATACGACTAAAAG | GCGGCTTGTGGGCATATTTCTT |
| miR5198 | GGGAGAAAGAGAGATTGTTGGGAG | GTCGTATCCAGTGCAGGGTCCGAGGTATTCGCACTGGATACGACCTCCCA | GATGCGGGAGAAAGAGAGATTGTT |
| miR394 | AGGTGGGGATGACGTCAAGT | GTCGTATCCAGTGCAGGGTCCGAGGTATTCGCACTGGATACGACACTTGA | AGAGCAGGTGGGGATGACGTCA |
| miR780 | TTCTTCTGAAGAACTGGCAT | GTCGTATCCAGTGCAGGGTCCGAGGTATTCGCACTGGATACGACATGCCA | AGCGCTTCTTCTGAAGAACTGGC |
| miR1222 | CTGAAAGATCATTGGTGACA | GTCGTATCCAGTGCAGGGTCCGAGGTATTCGCACTGGATACGACTGTCAC | AGCGCGCTGAAAGATCATTGGTG |
| miR6150 | CTTGTTTGATGGTATTTGCT | GTCGTATCCAGTGCAGGGTCCGAGGTATTCGCACTGGATACGACAGCAAA | GCGGCGCTTGTTTGATGGTAT |
| miR158 | TCTTAAATGTAGACAAAGCA | GTCGTATCCAGTGCAGGGTCCGAGGTATTCGCACTGGATACGACTGCTTT | GCCGCTCTTAAATGTAGACAAAGC |
| miR5176 | TGTGATGATGTGGCATTGACCGAT | GTCGTATCCAGTGCAGGGTCCGAGGTATTCGCACTGGATACGACATCGGT | GAGCTGTGATGATGTGGCATTGAC |
| miR6278 | TCATTGTACACAAGCTGAG | GTCGTATCCAGTGCAGGGTCCGAGGTATTCGCACTGGATACGACCTCAGC | AGCGCGTCATTGTACACAAGCT |
| miR7821 | AGATGGGCAAGGGCATTTGCA | GTCGTATCCAGTGCAGGGTCCGAGGTATTCGCACTGGATACGACTGCAAA | AGCAGAGATGGGCAAGGGCA |
| miR5029 | AATGACGAGAGAAACTGCA | GTCGTATCCAGTGCAGGGTCCGAGGTATTCGCACTGGATACGACTGCAGT | GTGCAATGACGAGAGAAACTGCA |
| miR5261 | TGATTTAGATGGCTTTGT | GTCGTATCCAGTGCAGGGTCCGAGGTATTCGCACTGGATACGACACAAAG | GCCGGTGATTTAGATGGCTTTG |
| miR7708 | TGTCATGAACTGAACGAAAGACG | GTCGTATCCAGTGCAGGGTCCGAGGTATTCGCACTGGATACGACCGTCTT | GAGCTGTCATGAACTGAACGAAAG |
| miR3438 | TCGATGCTTCATCTCGGACAC | GTCGTATCCAGTGCAGGGTCCGAGGTATTCGCACTGGATACGACGTGTCC | AGGCTCGATGCTTCATCTCGGA |
| miR1151 | ACTGGTTGTGGACACGGA | GTCGTATCCAGTGCAGGGTCCGAGGTATTCGCACTGGATACGACTCCGTG | AGAGCACTGGTTGTGGACACG |
| miR6219 | ATCAGGGACGAAAGTTGGG | GTCGTATCCAGTGCAGGGTCCGAGGTATTCGCACTGGATACGACCCCAAC | GAGCGCATCAGGGACGAAAGT |
| miR3437 | AAAAATACAAGGACTAAACGGAT | GTCGTATCCAGTGCAGGGTCCGAGGTATTCGCACTGGATACGACATCCGT | GCGCGCGAAAAATACAAGGACTAA |
| miR160 | TGCCTGGCTCCCTGTATGCCA | GTCGTATCCAGTGCAGGGTCCGAGGTATTCGCACTGGATACGACTGGCAT | GATTGCCTGGCTCCCTGTATG |
| Common reverse primer | | CAGTGCAGGGTCCGAGGT (5'→3') |  |
|  |  | Reverse primer (5'→3') | Forward primer (5'→3') |
| Actin |  | GCTTGGAGCAAGTGCTGTGATT | AGAACTATGAACTGCCTGATGGC |
