# Supplementary material for: MicroRNA-mediated responses to long-term magnesium-deficiency in Citrus sinensis roots revealed by Illumina sequencing
Source: BMC Genomics. 2017 Aug 24;18:657. doi: 10.1186/s12864-017-3999-5 (PMC5571589; doi:10.1186/s12864-017-3999-5)
Supplement: Supplementary file 10 — Specific primer pairs used for qRT-PCR expression analysis of selected miRNA target genes. (DOCX 31 kb) [file 12864_2017_3999_MOESM10_ESM.docx]

| **Additional file 10** Specific primer pairs used for qRT-PCR expression analysis of selected miRNA target genes | | | | | |
| --- | --- | --- | --- | --- | --- |
| miRNA | Accession | Homology | Target genes | Forward primer(5'→3') | Reverse primer(5'→3') |
| miR158 | orange1.1g022993m | AT5G62740.1 | SPFH/Band 7/PHB domain-containing membrane-associated protein family | CAGTTTCTGCGGTTTCTACG | ACAAGACAGCATCAGAAAGC |
| miR1222 | orange1.1g037429m | AT4G27220.1 | NB-ARC domain-containing disease resistance protein | CGTCACCACCACGCTTGCTA | TCTGAAACGCTCCCATCACA |
| miR2919 | orange1.1g002089m | AT3G14940.1 | Phosphoenolpyruvate carboxylase 3 | CCCCTTACCCTCACTATCTT | ACTCTTGAACCGTTTCTTTG |
| miR3437 | orange1.1g040557m | AT1G56140.1 | Leucine-rich repeat transmembrane protein kinase | TCTTGTGATAGGGGTTTATT | TAGTTGCCTTCTTCAGTGTC |
| miR7821 | orange1.1g010745m | AT1G29760.1 | Putative adipose-regulatory protein (Seipin) | ACTCTACGACGCCGTTCTAT | TCTTCGCTAACACCACTTCC |
| miR394 | orange1.1g000114m | AT1G20960.1 | U5 small nuclear ribonucleoprotein helicase, putative | ATGGCTTTGATGTTACTGGT | TCTTCACTCTTCCCCTGCTT |
| miR414 | orange1.1g004767m | AT1G17980.1 | Poly(A) polymerase 1 | GGGCAGCGATTGGGCATTAC | TCTGTAACTTCAGGCATCTC |
|  | orange1.1g006232m | AT1G17980.1 | Poly(A) polymerase 1 | TTGTAATGGGGAGCAGTAAC | TTCAAACCTTTTGCACGACT |
| miR418 | orange1.1g003146m | AT1G20780.1 | Senescence-associated E3 ubiquitin ligase 1 | GTTTGGTTATTTCAGGCATT | AATTGCACCATTTTCCTTCA |
| miR6150 | orange1.1g009434m | AT5G62810.1 | Peroxin 14 | CTCCGCCTTCTCCCAGTTCC | ATCGCCTATAAATAACTGGA |
|  | orange1.1g009573m | AT5G62810.1 | Peroxin 14 | CCGCCTTCTCCCAGTTCCAC | CACGGCGAAAAGCCTCATCT |
|  | orange1.1g018459m | AT3G28715.1 | ATPase, V0/A0 complex, subunit C/D | ATCACATCACAACGAACAAA | GGAAGGTTCGTTTTGGAGGT |
| miR6278 | orange1.1g005896m | AT3G14470.1 | NB-ARC domain-containing disease resistance protein | TGATCTGGTGTCAAGGTCAA | CATAGGAAGAATGGCGAACC |
|  | orange1.1g030696m | AT5G17840.1 | DnaJ/Hsp40 cysteine-rich domain superfamily protein | TTCATCTATTGTTCTGCCCATT | TTCTGCGTCTTTGCTCATTT |
| miR1847 | orange1.1g026316m | AT5G35530.1 | Ribosomal protein S3 family protein | GTGCCAAGGGATGTGAGTTT | GCCCTTGCTTGCCCTTTTGA |
|  | orange1.1g026835m | AT5G35530.1 | Ribosomal protein S3 family protein | GCTTCTTGGAGGTCTCGCTGTT | GCCCTTGCTTGCCCTTTTGA |
|  | orange1.1g029201m | AT2G31610.1 | Ribosomal protein S3 family protein | AGGGATGTGAGGTTATTGTG | GTCGATGTATTCATTCACAGGTTG |
| miR6028 | orange1.1g005923m  orange1.1g034040m  orange1.1g021729m  orange1.1g026539m | AT2G33580.1  AT5G42990.1  AT5G29100.1  AT1G79020.1 | Protein kinase superfamily protein  Ubiquitin-conjugating enzyme 18  Basic helix-loop-helix (bHLH) DNA-binding superfamily protein  Enhancer of polycomb-like transcription factor protein | ATAGCGTTTGTTAAGTGTCC  TGCTTCCTTTTAGTTTTGAT  ATATGGGGACAACTGAAAAT  TAATGAAAGACTGGAAGAAA | TGTTTGTTGCCCTTGTATTG  ATGCAAATTGATTAGACATG  GCTATACCTGGTTAGGAGCT  TGTTTGGGCTCTAACCTACT |
|  | orange1.1g045123m | AT4G35800.1 | RNA polymerase II large subunit | CTTGCTGACGAGGAAGATGA | TGACCAAGGAGACTAAGGAC |
|  | orange1.1g003175m | AT4G14700.1 | Origin recognition complex 1 | TTGGAAAAGAAGATGACCGT | TTGGAATTTGGCTTAGTTGG |
|  | orange1.1g006076m | AT3G46790.1 | Tetratricopeptide repeat (TPR)-like superfamily protein | CTCAGTTTGTTGCTGCGTGTC | ATAAGGAGTTGTGGTGGGTG |
|  | orange1.1g029970m | AT3G49940.1 | LOB domain-containing protein 38 | TCAAAGCATTCAATGGATCG | GCTCACTGGGTTCACTGTTC |
|  | orange1.1g028357m | AT2G45850.2 | AT hook motif DNA-binding family protein | CTTGGACATTTAGCTTCTCA | TCTCAACATTCAGCCGTATA |
| miR5176 | orange1.1g005789m | AT4G09140.1 | MUTL-homologue 1 | GTCAAAGGCACGCAGATAAT | TAGCAACCGACACTCCATAT |
|  | orange1.1g008397m | AT4G09140.1 | MUTL-homologue 1 | CGCAGATAATGGTGGAGAAT | CAAGCCTTGACGATGTAGCA |
|  | orange1.1g010846m | AT4G09140.1 | MUTL-homologue 1 | GTCAAAGGCACGCAGATAAT | TAGCAACCGACACTCCATAT |
|  | orange1.1g012406m | AT4G09140.1 | MUTL-homologue 1 | CGCAGATAATGGTGGAGAAT | GAATCAAGCCTTGACGATGT |
| miR7121 | orange1.1g005267m | AT1G71400.1 | Receptor like protein 12 | TGATGAGGCACCAGAACCAA | ATGCACCAACAAACTTCTTCTACC |
|  | orange1.1g005542m | AT1G71400.1 | Receptor like protein 12 | GGGTTACGCATCTGGAGTGG | ATGCACCAACAAACTTCTTCTACC |
|  | orange1.1g008628m | AT1G71400.1 | Receptor like protein 12 | CTTCACGCTGGTTTGATTGG | TACCTGCTCTGCCTCGTCTG |
|  | orange1.1g012980m | AT5G53390.1 | O-acyltransferase (WSD1-like) family protein | TGCTTAGTATCGTGTTCGTT | GATGATACAACCCCAAAGAG |
|  | orange1.1g013532m | AT5G53390.1 | O-acyltransferase (WSD1-like) family protein | TGCTTAGTATCGTGTTCGTT | GATGATACAACCCCAAAGAG |
|  | orange1.1g002167m | AT5G27060.1 | Receptor like protein 53 | CCTATTCTGGTAACTCGGGTCT | TACTTGCTCTGCCTCGTCTG |
|  | orange1.1g027358m | AT5G03080.1 | Phosphatidic acid phosphatase (PAP2) family protein | TACCGAAAGGGCGATCAGCT | TACTGGGAGTGACTGGAGGG |
|  | orange1.1g027353m | AT5G03080.1 | Phosphatidic acid phosphatase (PAP2) family protein | TACCGAAAGGGCGATCAGCT | GAATTGGGAGACTAAGAGGC |
| miR6190 | orange1.1g029300m | AT5G64200.1 | Ortholog of human splicing factor SC35 | GGGACAAGGATTACAGGAGG | GGGGAGGCACTTCAAGATGA |
|  | orange1.1g017284m | AT5G34850.1 | Purple acid phosphatase 26 | CCAAAAGCACCAATCCACTA | TGTCTTCTCCCTGTCCACCT |
|  | orange1.1g002842m | AT4G01810.1 | Sec23/Sec24 protein transport family protein | CAGAACCAAGCTAAGCAATG | TATCGGTGGGGACAAAATGA |
| miR6446 | orange1.1g016909m | AT5G09300.1 | Thiamin diphosphate-binding fold (THDP-binding) superfamily protein | GCCCAGTTGATGAGATTGAA | AGGGATAGTCTTGTGGGTGC |
|  | orange1.1g023827m | AT5G09300.1 | Thiamin diphosphate-binding fold (THDP-binding) superfamily protein | TCAGACTTGTTTACCGATGT | AGTGATGAAGCGTGCTATTA |
|  | orange1.1g001557m | AT5G20280.1 | Sucrose phosphate synthase 1F | TGAGGCAGCAGGGAAGGACA | CAGAAGCAGCCCAACGAACC |
|  | orange1.1g002665m | AT5G20280.1 | Sucrose phosphate synthase 1F | TCTACTGGTTTCGGGAGGTT | TGATTGTCTTCGGTGCTTGA |
| miR6485 | orange1.1g001969m | AT5G20730.2 | Transcriptional factor B3 family protein / auxin-responsive factor AUX/IAA-related | CACCACCAGCAACAGCATCA | GCAAGCCGCAGTAAAAGAGG |
|  | orange1.1g011274m | AT3G22810.1 | Plant protein of unknown function (DUF828) with plant pleckstrin homology-like region | AAACAACTCTTCTACATTCCCTT | CTTCTTGTATGACGCTGCTC |
|  | orange1.1g031218m | AT1G07400.1 | HSP20-like chaperones superfamily protein | CATTCCGCACCAGAAAGCAA | ACACCGCAGATGGAAAAGAA |
|  | orange1.1g009779m | AT1G08960.1 | Cation exchanger 11 | TCAATAACCACCCATTCATC | ATAGCCACAAAGCCAACAAC |
|  | orange1.1g029454m | AT5G51160.1 | Ankyrin repeat family protein | TAATAACAGTCAATCCGACC | AAGACGCTGAAGTATGAAAA |
|  | orange1.1g013633m | AT1G28560.1 | SnRNA activating complex family protein | CTCACTTCAAGGCTGTAGAC | GAATAACAGTAGTCGCAAAA |
|  | orange1.1g017698m | AT1G28560.1 | SnRNA activating complex family protein | GGTGAGTTACAACAAAAGCAA | AGCCCGACTATGTACGTCTT |
|  | orange1.1g042988m | AT5G62850.1 | Nodulin MtN3 family protein | CGGCAATAGTGAGACAAAAG | GGAGGAAAAGATTACGAAGA |
|  | orange1.1g007868m | AT1G72650.2 | TRF-like 6 | CAAACACCTCCAGATAGTCG | CATTACCACAGAAGGAAACC |
|  | orange1.1g046667m | AT2G38940.1 | Phosphate transporter 1;4 | GGTTCGGCATTGGTGGTGAC | GGGCGGTTGAAGCAATAGGA |
|  | orange1.1g001289m | AT1G14610.1 | Valyl-tRNA synthetase / valine--tRNA ligase (VALRS) | AATCTTACTCTTGTCGCCTCT | CTTCTCCTTAGCCTTTTCCT |
|  | orange1.1g001303m | AT1G14610.1 | Valyl-tRNA synthetase / valine--tRNA ligase (VALRS) | AATCTTACTCTTGTCGCCTCT | CAGTTCCTTCTCCTTAGCCT |
|  | orange1.1g001757m | AT1G14610.1 | Valyl-tRNA synthetase / valine--tRNA ligase (VALRS) | AATCTTACTCTTGTCGCCTCT | GTTCCTTCTCCTTAGCCTTT |
|  | orange1.1g024117m | AT2G47920.1 | Kinase interacting (KIP1-like) family protein | TTGGAAGAAGGGGAAGTCAT | TTTTGGTTGTTGCTTGGGTG |
|  | orange1.1g036588m | AT4G20140.1 | Leucine-rich repeat transmembrane protein kinase | TTCCTTACGCTGGACATCTA | GGAAAACATTTGTAGGGAGT |
|  | orange1.1g003591m | AT5G05680.1 | Nuclear pore complex protein-related | CCTTGCTTGCTTCTCCCTTTA | CGCTGGGTTGAGTATTTGGT |
| miR1044 | orange1.1g001378m | AT1G10170.1 | NF-X-like 1 | CAGAGCATCAATCCGAATAC | AAACAACTCGAACAAGACCA |
|  | orange1.1g001377m | AT1G10170.1 | NF-X-like 1 | CAGAGCATCAATCCGAATAC | AAATCCAGGTCCTTTGGTAG |
|  | orange1.1g047796m | AT2G38380.1 | Peroxidase superfamily protein | TTTCAGGGGCAGATTATACGA | CACCTTGTGGGCATAGTTCC |
|  | orange1.1g042193m | AT5G03340.1 | ATPase, AAA-type, CDC48 protein | ATATTACTGAGATATGTCAGCGTG | TGGTATTTTCGGATGTCTGC |
|  | orange1.1g019546m | AT2G40340.1 | Integrase-type DNA-binding superfamily protein | GGTCTAAGAGGGCTGGGATA | GCGGTAGCTGGCTTTAACAA |
| miR5198 | orange1.1g002063m | AT1G72180.1 | Leucine-rich receptor-like protein kinase family protein | GTTCTTTCTCCCTCCAATCT | GACCTTAGAAACAGCACCAA |
| miR5029 | orange1.1g012168m | AT5G53450.1 | OBP3-responsive gene 1 | ATATTTATCGCCAGCCTCAG | AGTTCAAGGTTCAAAAGTGA |
|  | orange1.1g026587m | AT4G31300.3 | N-terminal nucleophile aminohydrolases (Ntn hydrolases) superfamily protein | GGAAAGAGGGAATGACCAAG | TGTTCATCGGCTCAGGACTA |
|  | orange1.1g029964m | AT4G31300.3 | N-terminal nucleophile aminohydrolases (Ntn hydrolases) superfamily protein | GTCTCCGACTATGTTCGCTAC | CCTTGGTCATTCCCTCTTTC |
|  | orange1.1g030788m | AT4G31300.3 | N-terminal nucleophile aminohydrolases (Ntn hydrolases) superfamily protein | GGAAAGAGGGAATGACCAAG | TGTTCATCGGCTCAGGACTA |
|  | orange1.1g014625m | AT3G23510.1 | Cyclopropane-fatty-acyl-phospholipid synthase | AAAGAAGAGGGGTTGGTGGT | TTCGCTCAGATCATAATGACGAGA |
|  | orange1.1g018123m | AT3G44160.1 | Outer membrane OMP85 family protein | AGCGGGAGTAACCACGACAG | TCCAACAGCACCCTCACCAT |
| miR5261 | orange1.1g018132m | AT3G56930.1 | DHHC-type zinc finger family protein | GGTGCCCAGAAATTCAAAGC | CACGAGGAGGACGATAAAGC |
|  | orange1.1g010695m | AT3G12640.1 | RNA binding (RRM/RBD/RNP motifs) family protein | TGATGCTCCCCGACGGTTAC | TCCGACGACGTTCAGCCACT |
|  | orange1.1g011967m | AT3G12640.1 | RNA binding (RRM/RBD/RNP motifs) family protein | TGATGCTCCCCGACGGTTAC | TCCGACGACGTTCAGCCACT |
|  | orange1.1g031636m | AT1G67620.1 | Lojap-related protein | CAGTGTTCAAGGGCAGGATA | CTGTTGCTGATTTAGATGGC |
|  | orange1.1g033883m | AT1G67620.1 | Lojap-related protein | AAAGAGGGAATGACCAAGGA | CTCATGCCACAATGGAAGTG |
|  | orange1.1g004959m | AT5G66850.1 | Mitogen-activated protein kinase kinase kinase 5 | CTATCGCCAGCGTGTTTTCT | CATCCCACCAACACTCCTTT |
|  | orange1.1g043928m | AT2G36110.1 | Polynucleotidyl transferase, ribonuclease H-like superfamily protein | AGTTCAATCACAGATGCACCAA | CCTAGCAGCAAAATGCCCTA |
|  | orange1.1g004713m | AT5G54260.1 | DNA repair and meiosis protein (Mre11) | GGACAACAGCCCAGGGAGGA | GGCAATGGCGACGAAGAATC |
|  | orange1.1g010785m | AT3G26020.2 | Protein phosphatase 2A regulatory B subunit family protein | CCTCTACGTCCTCTTCACAT | TCACTCCTACATTAGCAACA |
|  | orange1.1g000012m | AT1G55860.2 | Ubiquitin-protein ligase 1 | AACCGTTTGAAGGGGTAAAT | ATATGGAAGAACAGGGGCAT |
|  | orange1.1g000013m | AT1G55860.2 | Ubiquitin-protein ligase 1 | CTGTTTGGTCTTCGCAGTAG | AACTCCCAGATGACAAAGCT |
|  | orange1.1g029528m | AT5G01520.1 | RING/U-box superfamily protein | TGAATTACTTTACCCCGTCA | TACCATTTCAGTTCCCTACC |
|  | orange1.1g029508m | AT1G22360.1 | UDP-glucosyl transferase 85A2 | GCTCATGCAGTATGCGTTCC | AAAATCGGGCAGACCCTTTA |
|  | orange1.1g037980m | AT2G36110.1 | Polynucleotidyl transferase, ribonuclease H-like superfamily protein | AAAGTTCAACCACAGATGCACCAA | ATCCCCGCAAAGCAAATCGT |
| miR3438 | orange1.1g000163m | AT1G55325.2 | RNA polymerase II transcription mediators | TGAATACTTGGAGTGGCAGAA | ATGGACTAGGTGAGGACGGT |
| miR1151 | orange1.1g018149m | AT5G49610.1 | F-box family protein | AGGCGTTTGTCGTGACTGGA | GATGGTTCGCTGGTGGATGA |
|  | orange1.1g018125m | AT5G49610.1 | F-box family protein | AGGCGTTTGTCGTGACTGGA | AAATGATGGTTCGCTGGTGG |
|  | orange1.1g023739m | AT2G41870.1 | Remorin family protein | TGGGAAACAAGCAGCCATCG | CCCCGGACCCAAATCTAACG |
|  | orange1.1g027436m | AT2G41870.1 | Remorin family protein | TGGGAAACAAGCAGCCATCG | AAGAACCAAAGCGTTAAATT |
|  | orange1.1g023033m | AT2G36690.1 | 2-oxoglutarate (2OG) and Fe(II)-dependent oxygenase superfamily protein | TCAATGCTCCTCAAAACCCT | GCAGCCCCATCACTACTTCT |
|  | orange1.1g026453m | AT1G17020.1 | Senescence-related gene 1 | AAGTTGCCTCTTCCATTCAG | GAGTACGGAGCCATCAGAAT |
|  | orange1.1g020233m | AT2G36690.1 | 2-oxoglutarate (2OG) and Fe(II)-dependent oxygenase superfamily protein | AGTTGGATTCAGTGAGATTT | TTGACATTGACCCACTTTCC |
|  | orange1.1g037473m | AT5G07480.1 | KAR-UP oxidoreductase 1 | ATGGATGAAGGAATGCAAGT | GGGTAGCCCTATGAACAACG |
| miR6219 | orange1.1g010903m | AT5G15130.1 | WRKY DNA-binding protein 72 | CAACCTCCACGGATTGAACT | GAGAAAGTGGCTGGTGCTGT |
| miR7708 | orange1.1g023136m | AT1G06890.1 | Nodulin MtN21 /EamA-like transporter family protein | GCAAGAGGATAATGGTGTCT | TATGTTTTCAAAGCAATGAG |
| miR780 | orange1.1g044623m | AT5G17230.2 | PHYTOENE SYNTHASE | GAATCGACTTGCCTGAAATA | AGATCACGAGTAACCCCACT |
|  | orange1.1g004573m  orange1.1g030826m | AT4G27220.1  AT2G26560.1 | NB-ARC domain-containing disease resistance protein  Phospholipase A 2A | CCAACACTAAAAGCCTCCTC  GCAGAAGGAAAGAAAATAAC | AATCAAACCACTTCCCTAAA  GAAACGTCGTACACCATAAT |
| miR160 | orange1.1g005482m  orange1.1g004896m  orange1.1g005075m  orange1.1g008078m | AT4G30080.1  AT2G28350.1  AT4G30080.1  AT1G77850.1 | Auxin response factor 18  Auxin response factor 10  Auxin response factor 18  Auxin response factor 17 | TAACCAACTTCCAACACCATCAT  TCACCAGCAAGAAAGAAGTT  GAAGTCAAGACTGCCACAACCA  TCTTTCCAGTGAACGGTTTAGG | TGAAAGCCCAGTGGAAACAG  CTGGATAGAAACAGCCCTGA  TTGTGGGTTTAGCAGCACGATC  GACATCTGATGATTATTCTCGCTTA |
